# Diaryltin Dihydrides and Aryltin Trihydrides with Intriguing Stability

**DOI:** 10.3390/molecules25051076

**Published:** 2020-02-27

**Authors:** Beate G. Steller, Berenike Doler, Roland C. Fischer

**Affiliations:** Graz University of Technology, Stremayrgasse 9/V, 8010 Graz, Austria; b.doler@student.tugraz.at (B.D.); roland.fischer@tugraz.at (R.C.F.)

**Keywords:** main group chemistry, tin hydrides, organotin chemistry, NMR spectroscopy, X-ray crystallography

## Abstract

In the last few decades, organotin hydrides have proven their potential as building blocks for a great variety of organometallic compounds. In this context, organotin hydrides with sterically shielding aryl substituents have attracted special interest, as these ligands can kinetically stabilize metastable products. The selective synthesis of aryltin halide compounds Ar*_2_SnCl_2_ and Ar*SnI_3_ featuring the highly sterically encumbered aryl ligand Ar* (*^i^*^Pr^Ar* = 2,6-(Ph_2_CH)_2_-4-*i*PrC_6_H_2_; ^Me^Ar* = 2,6-(Ph_2_CH)_2_-4-MeC_6_H_2_) is presented. These aryltin halides were converted into corresponding aryltin hydrides Ar*_2_SnH_2_ and Ar*SnH_3_, which exhibit a surprisingly high thermal stability and oxygen tolerance.

## 1. Introduction

**Figure 1:**
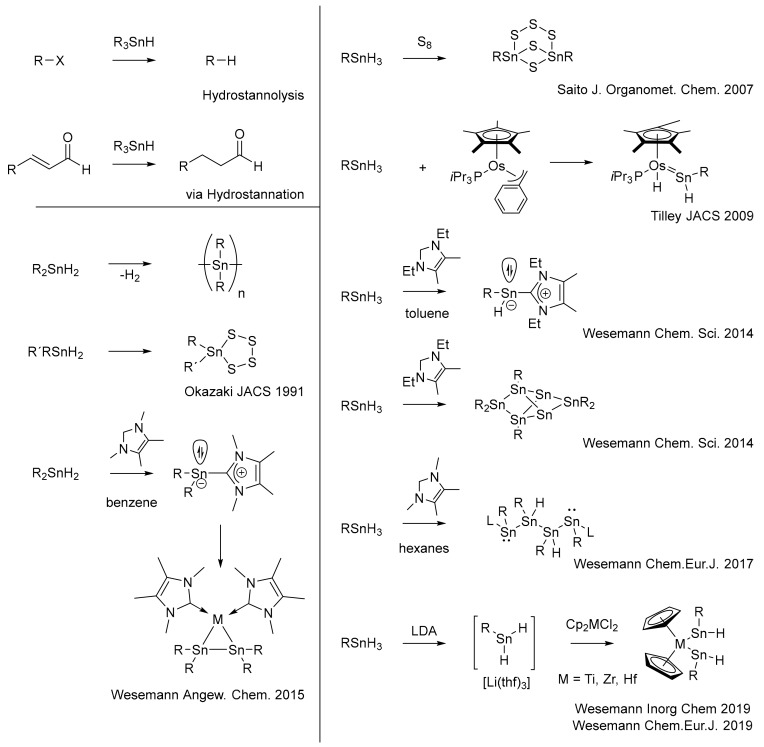
Organotin hydrides and their applications and reactivities.

Compared to their carbon analogue, the element–hydrogen bond of heavier group 14 elements exhibits a far different stability and chemical behavior. [1] Thereby, the tin–hydrogen bond represents a fascinating border in group 14, as they show a wider range of possible reaction mechanisms than the corresponding germanium or silicon bond but are more readily accessible than lead hydrides [2,3]. While organotin hydrides R_n_SnH_4-n_ are stable against moisture, they are usually thermolabile and readily react with oxygen to form the corresponding hydroxides or oxides. Their tolerance to oxygen and heat increases with increasing number of organic substituents and is additionally enhanced by organic substituents, which offer some steric protection [4]. Despite, or precisely because of, their labile nature, they have found wide application in organic synthetic chemistry, e.g., in hydrostannolysis and hydrostannation, as well as in organometallic chemistry as prominent starting materials for the synthesis of transition metal and main group compounds [5]. In the last few decades, (catalytic) dehydrogenative coupling of diorganotin diyhdrides has attracted wide interest in the preparation of oligo- and polystannanes [6,7,8]. More recent synthetic efforts have shown their intriguing reactivity and their potential as starting materials for corresponding low oxidation compounds, polyhedral cages and clusters or mixed-metal compounds (Figure 1).

The reactivity of tin hydrides was exploited in the reaction of an unsymmetrically substituted diaryltin dihydride R’RSnH_2_ (R´ = Tbb = 2,6-[(Me_3_Si)_2_HC]_2_-4-*t*BuC_6_H_2_, R = Mes = 2,4,6-Me_3_C_6_H_2_) with elemental sulfur to give access to a tetrathiastannolane [9]. Later ArSnH_3_ (Ar = 2,6-Tripp_2_C_6_H_3_; Tripp = 2,4,6-*i*Pr_3_C_6_H_2_) was converted with elemental sulfur to give a bicyclic tin/sulfur cage at elevated temperatures [10]. Tilley et al. obtained an Os = Sn double bond by the reaction of an TrippSnH_3_ and Cp*(*i*Pr_3_P)(H)Os(CH_2_Ph) [11]. Similarly, a Ru = Sn double bond was accessed from Cp*Ru(IXy-H)N_2_ (IXy = 1,3-bis(2,6-dimethylphenyl)imidazol-2-ylidene) using TrippSnH_3_ as well [12]. The group of Wesemann performed reactivity studies of N-heterocyclic carbenes (NHCs) with diaryltin diyhdrides and aryltin trihydrides. Products in these conversions do not only depend on the organic substituents on tin and the NHC, but also on stoichiometric ratios of tin hydride to applied NHC and the solvent. In the course of this work, access to NHC-stabilized low valent tin(II) compounds was granted starting from Tripp_2_SnH_2_ to give Tripp_2_Sn(NHC) and from RSnH_3_ to give RSnH(NHC) (R = Tripp, 2,6-Mes_2_C_6_H_3_, 2,6-Tripp_2_C_6_H_3_, (Me_3_Si)_2_CH) [13]. The low-valent species R_2_Sn(NHC) turned out to be a convenient synthon for Pt, Pd and Ni containing distannametallacyclopropanes [14]. Moreover, neutral and charged metalloid clusters as well as NHC-stabilized Sn chains in variable length (2–4 tin atoms in a row) with two stannyl-stannylene sites have been obtained from dehydrogenative coupling of RSnH_3_ (R = (Me_3_Si)_2_CH) [13,15]. Lately, reaction of a sterically encumbered ArSnH_3_ (Ar = 2,6-TrippC_6_H_3_) with [Ph_3_C][Al(OC{CF_3_}_3_)_4_] gave an organodihydrostannylium salt [ArSnH_2_][Al(OC{CF_3_}_3_)_4_], which readily eliminates H_2_ when warming up to room temperature and gives the low valent organotin cation [ArSn]^+^ [16]. Corresponding dihydridostannate anions were obtained by deprotonation of the same aryltin trihydride using lithium diisopropylamide (LDA). This organodihydridostannate reacts as a nucleophile with low valent group 14 electrophiles [17] and with metallocene dichlorides of Ti, Zr and Hf to give corresponding metallocene bis(hydridoorgano-stannylene) complexes [18].

The first successfully isolated organotin hydride, Me_3_SnH, was accessed by treatment of the corresponding organotin sodium compound with ammonium chloride in liquid ammonia [19]. Still, after these initial investigations, tin hydrides were nearly neglected due to their challenging and specific synthesis and instability, until Finholt and co-workers applied LiAlH_4_ to generate SnH_4_, MeSnH_3_, Me_2_SnH_2_ and Me_3_SnH from the corresponding chlorides next to other group 14 element hydride compounds [20]. In a similar fashion, a range of organotin hydrides were accessed utilizing DIBAL-H as the hydride transfer reagent shortly afterwards [21]. Nevertheless, organotin hydrides are still usually accessed via the straightforward hydride transfer of the corresponding tin halide using LiAlH_4_. More rarely, other metal hydrides like NaBH_4_, LiH, (MeSiHO)_n_, Et_3_SiH, R_3_SnH are utilized^4^. Therefore, once the organotin halides are in hand, the corresponding hydrides are readily available. 

Herein, we report the synthesis of diaryltin dihalides and aryltin trihalides and corresponding hydrides featuring a recently introduced bulky ligand backbone Ar* (*^i^*^Pr^Ar* = 2,6-(Ph_2_CH)_2_-4-*i*PrC_6_H_2_; ^Me^Ar* = 2,6-(Ph_2_CH)_2_-4-MeC_6_H_2_) starting from the corresponding arly iodine Ar*I [22]. From the direct salt metathesis of in-situ prepared aryllithium solutions and SnCl_4_, we were able to isolate solely the diaryltin dichloride Ar*_2_SnCl_2_ after recrystallization in good yields. In contrast, analytically pure aryltin triiodide Ar*SnI_3_ was formed in conversions of the same aryllithium species and SnCl_2_ (in a stoichiometric ratio 1:1.1) and subsequent oxidative addition with I_2_. Isolated aryltin halides were converted into the corresponding hydride species using LiAlH_4_ for tin dihydrides and DIBAL-H for tin trihydride species. All isolated compounds were fully characterized by heteronuclear NMR and IR spectroscopy as well as single crystal X-ray diffraction.

## 2. Results

### 2.1. Synthesis and Spectroscopic Data

Due to their sterically demanding carbon backbone, anilines Ar*NH_2_ (Ar* = 2,6-(Ph_2_CH)_2_-4-R-C_6_H_2_, R = e.g., Me, *i*Pr) have been applied as sterically shielding amide ligands for low oxidation state group 14 compounds [23,24,25]. Just recently, corresponding Ar*I was isolated and applied in the synthesis of sterically encumbered primary phosphanes [22]. Nevertheless, activation of the rather acidic C-H bond in the CHPh_2_ moiety is likely to happen [26]. Possible modification of this ligand backbone and hence substitution of the rather acidic C-H in the CHPh_2_ moiety was just recently displayed [27]. In order to rule out potential metalation of the rather benzylic C-H bond by lithium organyls, the targeted synthesis of Ar*Li was first conducted. Treatment of solutions of *^i^*^Pr^Ar*I (**1**) or ^Me^Ar*I (**2**) in toluene with stoichiometric amounts of *n*BuLi led to precipitation of *^i^*^Pr^Ar*Li (**3**) and ^Me^Ar*Li (**4**), respectively, from these solutions. Recrystallization of these powders from Et_2_O gave crystals suitable for X-ray crystallography. NMR data as well as solid state structures corroborated lithiation of Ar*I by metal–halogen exchange rather than metalation of the C-H bond. 

**Figure 2:**
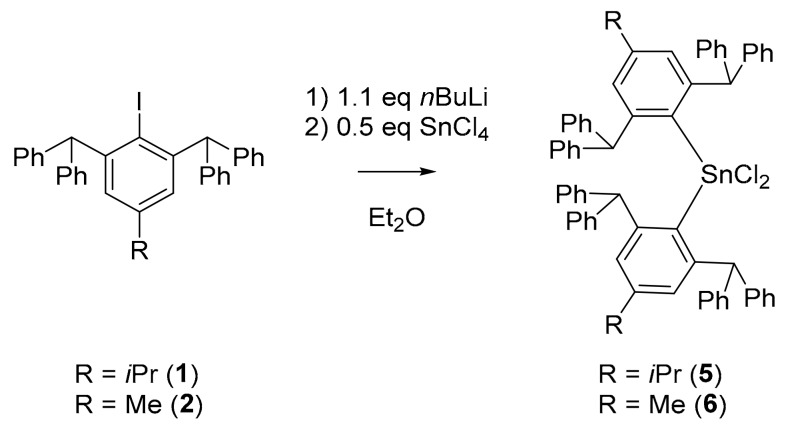
Synthesis of *^i^*^Pr^Ar*_2_SnCl_2_ (**5**) and ^Me^Ar*_2_SnCl_2_ (**6**) from *^i^*^Pr^Ar*I (**1**) and ^Me^Ar*I (**2**), respectively.

Therefore, synthesis of desired aryltin dichlorides **5** and **6** turned out the be straightforward. Following known procedures [28], the aryl iodides **1** and **2** were individually treated with a slight excess of *n*BuLi (1.15 eq) in Et_2_O at low temperatures, followed by subsequent addition of SnCl_4_ (0.5 eq) at low temperatures. Mixtures of halide species Ar_2_SnCl_2_, Ar_2_SnICl and Ar_2_SnI_2_ were expected in crude products [28]. Nevertheless, NMR investigations of the crude product after extraction of an aliquot with CDCl_3_ revealed the formation of mainly one tin species (see Supplementary Materials; p S2, Figures S1 and S2). In agreement with literature values, the main products were first identified to be Ar*_2_SnCl_2_ **5** and **6**, which was later confirmed by X-ray crystallography (Figure 2). In contrast to Molloy et al. [28], who observed the sole formation of the corresponding distannane BrAr_2_SnSnAr_2_Br when using the less sterically hindered carbanion 2,4,6-iPr_3_C_6_H_2_Li with SnBr_4_ in THF, we were not able to identify significant amounts of distannane in crude products with the highly sterically crowded Ar*Li.

**Figure 3:**
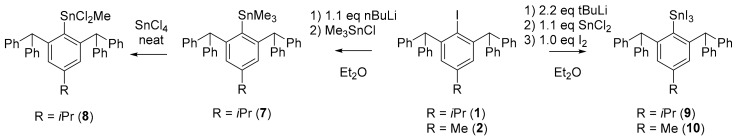
Preparation of arlytin compounds **7** and **8** as well as aryltin triioides **9** and **10**.

Synthesis of the corresponding aryltin trihalides by simply adjusting the stoichiometric ratio of Ar*Li and SnCl_4_ was unsuccessful. Interestingly, reactions with a Ar*Li:SnCl_4_ ratio of 0.9:1 and slow addition of the aryllithium species to SnCl_4_ in Et_2_O at low temperature yielded again Ar*_2_SnCl_2_ as the main product alongside unreacted SnCl_4_. Similar results were also observed when changing the solvent to THF or toluene. As an alternative synthetic method [29,30], the reaction of *^i^*^Pr^Ar*SnMe_3_ (**7**), obtained from conversions of *^i^*^Pr^Ar*Li with Me_3_SnCl, with an excess of tin tetrachloride was investigated (Figure 3). Yet, even after prolonged reaction times and high excess of SnCl_4_, only the dichloride *^i^*^Pr^Ar*SnCl_2_Me (**8**) was isolated from these approaches. Also, addition of catalytic amounts of AlCl_3_ did not lead to the transfer of the remaining methyl group and formation of the desired *^i^*^Pr^ArSnCl_3_. Inspired by the synthesis of an ArSnI_2_Cl (Ar = 2,6-(Me_2_NCH_2_)_2_C_6_H_3_) from ArSnCl and I_2_ [31], stoichiometric amounts of Ar*SnX (X = Cl, I), prepared in situ from Ar*Li and SnCl_2_ (1:1), and I_2_ were converted in order to yield an aryltin trihalide. The fading purple color after addition of I_2_ indicated an already ongoing reaction. Finally, analytically pure aryltin triiodide species **9** and **10** were obtained (68 and 53%) from these reactions after recrystallization from toluene/*n*-heptane and DCM/*n*-heptane, respectively. Interestingly, in these approaches, again no mixtures of halide species Ar*SnX_3_ (X = Cl or I) were observed in crude products, while a second tin species present in crude products with a highly lowfield-shifted resonance was assigned to be Ar*_2_SnI_2_ (for ^iPr^Ar*: δ = −498 ppm; for ^Me^Ar*: δ = −496 ppm) in agreement with literature values [32] (see Supplementary Materials, p S3, Figure S3). Conversion of an aliquot of such a crude product gained in the synthesis of **9** with LiAlH_4_ and subsequent NMR investigation of reaction solutions revealed the formation of small amounts of *^i^*^Pr^Ar*_2_SnH_2_ (**11**), as the expected product from the corresponding tin halide present in the mixture, next to the main product *^i^*^Pr^Ar*SnH_3_ (**13**) (see Supplementary Materials, p S3, Figure S4). These products were identified based on NMR data similar to literature values and later corroborated by the synthesis of **11** and **13**. Formation of Ar*_2_SnI_2_ was almost prevented when using a small excess of SnCl_2_ in the first step of this synthesis (see Supplementary Materials, p S4, Figure S5).

**Figure 4:**
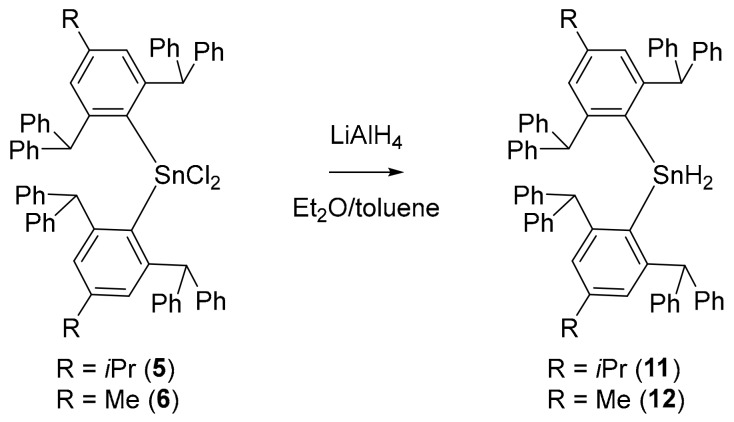
Hydrogenation of diaryltin dichlorides **5** and **6** utilizing LiAlH_4_ leads to corresponding diaryltin dihydrides **11** and **12**, respectively.

Diaryltin dihydrides **11** and **12** were accessed via the widely applied hydride transfer with LiAlH_4_ of the corresponding chlorides **5** and **6** and subsequent aqueous work-up with degassed dilute H_2_SO_4_. Reactions were carried out in a solvent mixture of Et_2_O/toluene due to low solubility of **5** and **6** in Et_2_O alone. (Figure 4) After recrystallization from toluene/*n-*pentane, **11** and **12** were isolated in fair yields (50% and 53% respectively).

**Figure 5:**
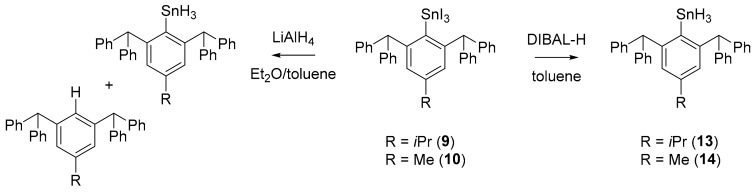
Hydrogenation of aryltin triiodies **9** and **10** with LiAlH_4_ leads to a mixture of the corresponding hydride and hydrolyzed ligand. Using a softer hydride transfer reagent like DIBAL-H gives access to **13** and **14**.

When applying the same synthetic protocol in conversions of **9** and **10** to corresponding aryltin trihydrides, even during short reaction times the formation of a grey solid—presumably elemental tin—was observed. From these reactions only grey, nearly black, crude products were isolated with the desired aryltin trihydrides **13** and **14** in only up to 70% purity. Hydrolyzed ligand Ar*H was identified as the main byproduct by ^1^H NMR. In reactions with LiAlH_4_ neither the use of substoichiometric amounts of LiAlH_4_ nor omitting the common aqueous work-up could improve these results. Yet, utilization of DIBAL-H instead of LiAlH_4_ as a softer hydride transfer agent gave satisfying results. After removal of all volatiles under reduced pressure and extraction of the resulting solid with *n*-pentane to remove byproducts and excess of DIBAL-H, **13** and **14** were isolated in satisfying yields (50% and 56% respectively). (Figure 5) In contrast to corresponding halide compounds, aryltin hydrides are sensitive to oxygen and easily react with traces to give corresponding hydroxide and oxide compounds. Yet, their tolerance against oxygen and temperature can be significantly increased by the steric demand of the substituting ligands^4^. Isolated diaryltin dihydrides **11** and **12** can be handled for a short period in air and possess an extraordinary thermal stability with melting points up to 200 °C. Likewise, isolated trihydrides **13** and **14** featuring this sterically crowded ligand Ar* possess astonishingly increased decomposition temperatures of up to 100 °C, while the parent PhSnH_3_ slowly decomposes even at low temperatures [21]. 

In agreement with literature values [32], ^119^Sn NMR shifts of isolated diaryltin dichlorides [−65.96 (**5**), −64.65 (**6**) ppm] are shifted ca. 40 ppm upfield compared to sterically less shielded diphenyltin dichloride (δ = −26.4 ppm). Yet, substitution of an aryl ligand by a methyl group results in a roughly 80 ppm downfield-shifted signal, as exemplified by resonances of Ph_2_SnCl_2_ [33] (−26.4 ppm) and PhSnMeCl_2_ [34] (55.53 ppm) and here again observed for **5** (−65.96 ppm) and **8** (15.07 ppm). Nevertheless, the highly upfield-shifted resonances of corresponding diaryltin diiodides (−498.22 and −496.98, respectively; found in crude products of Ar*SnI_3_) demonstrate a much stronger dependence on the nature of halide substituents, which is in full agreement with literature [32]. Even higher shifted resonances are observed for aryltin triiodides **9** and **10** (−937.27 and −939.57 ppm, respectively). Again, this agrees with NMR shifts for PhSnI_3_ [35] (−699.9 ppm) and Ar^CN^SnI_3_ [36] (−944.38 ppm).

^119^Sn NMR resonances and coupling constants of isolated tin hydrides are in full agreement with literature data. Resonances of shifts of diaryltin dihydrides **11** (−331.30 ppm) and **12** (−331.51 ppm) are shifted by ca. 100 ppm upfield compared to parent Ph_2_SnH_2_. Therefore, these values agree with resonances for species with less sterically demanding *ortho*-alkyl groups of the aryl substituents, e.g., Tripp_2_SnH_2_ [13] (Tripp = 2,4,6-*i*Pr_3_C_6_H_2_; −351.2 ppm). Substitution of an aryl group by a hydride leads to an upfield-shifted resonance for **13** (−406.06 ppm) and **14** (−406.67 ppm). These values are consistent with ^119^Sn NMR shifts found for TrippSnH_3_ (−416 ppm) and (2,6-Tripp_2_C_6_H_3_)SnH_3_ (−384.7 ppm). An summary of spectroscopic data is provided in Table 1. 

### 2.2. Solid State Structures

**Figure 6:**
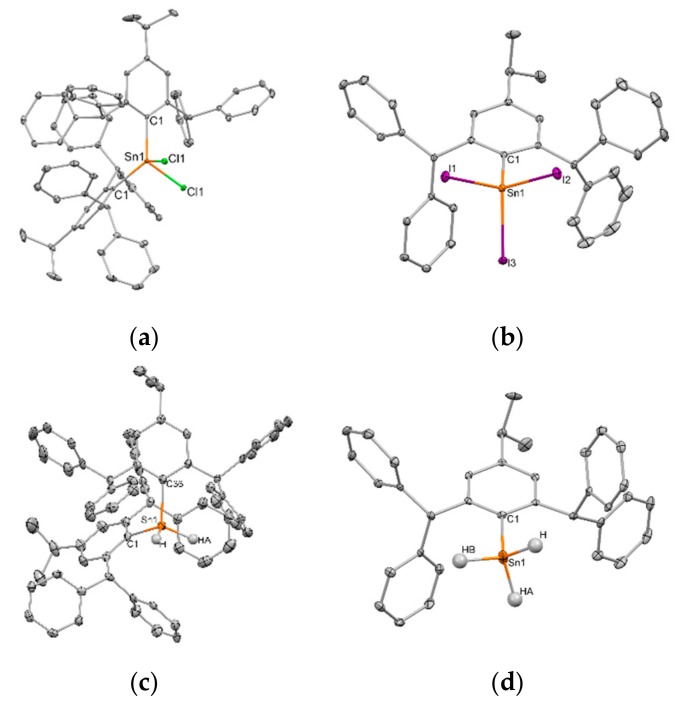
Solid state structures of (**a**) **5**, (**b**) **9** and their corresponding tin hydride compounds (**c**) **11**, (**d**) **13**. All nonhydrogen atoms shown as 30% shaded ellipsoids. Hydrogen atoms except for those bonded to Sn are omitted for clarity. Selected bond lengths (Å) and angles (°) are displayed in Table 2 and Table 3.

All isolated arlytin halides and hydrides have been structurally characterized by X-ray crystallography. An overview of selected bond lengths and angles of isolated aryltin halides **5**, **6, 8, 9** as well as **10** is provided in Table 2. Irrespective of the substituent or number of aryl substituents, the tin atom is found in a distorted tetrahedral environment (Figure 6). The C-Sn-C angles of diaryltin dichlorides **5** (125.77(7)°) and **6** (119.5(2)°) are widened and deviate significantly from the ideal tetrahedral geometry. Therefore, Cl-Sn-Cl angles (94.49(3)° for **5** and 95.82(5) ° for **6**) are sharper than in less sterically crowded Ph_2_SnCl_2_ [37] (101.7(1)°) or Mes_2_SnCl_2_ [38] (Mes = 2,4,6-Me_3_C_6_H_2_; 100.29(7)°), but are in agreement with diaryltin dichlorides featuring aryl groups with bulky *ortho*-substituents, e.g., Tripp_2_SnCl_2_ [39] (120.4(2)°) and Mes*_2_SnCl_2_ [40] (Mes* = 2,4,6-*t*Bu_3_C_6_H_2_; 95.5(1)°). A similar acute C-Sn-C angle is observed in **8** (92.47(7)°) despite the reduced sterical demand of the methyl substituent compared to a second *^i^*^Pr^Ar* substituent. The Sn-C^Ar^ bond length of **8** (2.140(6) and 2.141(7) Å, two independent molecules in the asymmetric unit) is slightly elongated compared to Ph_2_SnCl_2_ (2.112(5) Å). Likewise, Sn-C bonds of **5** (2.1501(5) Å) and **6** (2.155(5) and 2.159(6) Å) are elongated compared to diaryltin dichlorides with no or less sterically demanding *ortho*-alkyl groups of the aryl subsitutent, e.g., Ph_2_SnCl_2_ (2.112(5) Å) and Mes_2_SnCl_2_ (2.117(2) Å), but agree with values of sterically encumbered Mes*_2_SnCl_2_. Similar Sn-C bond lengths are found in aryltin triiodides **9** (2.161(2) Å) and **10** (2.158(3) Å). Notably, **9** and **10** represent, next to recently reported Ar^CN^SnI_3_ (Ar^CN^ = 2-(Me_2_NCH_2_)C_6_H_4_), the only structurally authenticated aryltin triiodides, so far [36]. In general, the number of organotin triiodides is limited to a total number of thirteen including various structures of alkyltin triiodides MeSnI_3_ and EtSnI_3_ coordinated by different Lewis bases [41]. The Sn-C bond values for **9** and **10** are elongated compared to corresponding bonds in Ar^CN^SnI_3_ (2.136(4) Å) and (2,6-Mes_2_C_6_H_3_)SnCl_3_ [42] (2.128(5) Å), but show similar values as in (2,6-Tripp_2_C_6_H_3_)SnCl_3_ [43] (2.154(5) Å). Average Sn-I bond lengths of **9** and **10** are shorter than in the molecular structure of Ar^CN^SnI_3_, a 5-fold coordinated tin species additionally coordinated by the amino functionality on the attached ligand. Average values for X-Sn-X angles in **9** (97.31(1) to 107.66(1)°) and **10** (95.665(12) to 105.743(11)°) are wider as in Ar^CN^SnI_3_ (92.693(12) to 110.641(13)°) and aryltin trichlorides (2,6-Mes_2_C_6_H_3_)SnCl_3_ (97.48(8) to 102.95(9)°) and (2,6-Tripp_2_C_6_H_3_)SnCl_3_ (98.12(6) to 102.96(7)°). Presumably due to the steric demand of the ligand, there is a total absence of any intermolecular interaction between X∙∙∙Sn in **5**, **6, 8, 9** as well as **10**.

Due to their high reactivity towards oxygen, the number of aryltin hydride compounds, which were structurally authenticated, is low, including only the structures of Ph_2_SnH_2_ [44] (crystallized from an in-situ technique in a capillary), Mes_2_SnH_2_ [45], Dep_2_SnH_2_ (Dep = 2, 6-Et_2_C_6_H_2_), Dipp_2_SnH_2_ (Dipp = 2, 6-*i*Pr_2_C_6_H_2_) as well as Tripp_2_SnH_2_ [32] and in the case of aryltin trihydrides is limited to the structures of TrippSnH_3_ [13] and (2,6-Tripp_2_C_6_H_3_)SnH_3_ [46]. Similar to all isolated aryltin halides, corresponding hydride compounds are found in a distorted tetrahedral environment (Figure 6). Selected bond lengths of **11** and **12** are summarized in Table 3. The Sn-C bonds in tin hydrides **11** (2.187(3) and 2.171(3) Å) and **12** (12.188(2) and 2.186(2) Å) are elongated compared to corresponding distances in diaryltin dichlorides **5** and **6**. Yet, such a trend is not observed for **13** (2.1538(2) Å) and **14** (2.167(3) Å) and their corresponding aryltin triiodides **9** (2.161(2) Å) and **10** (1.58(3) Å). The C-Sn-C angles of **11** (105.9(1)°) and **12** (109.49(8)°) are more acute by approximately 20° and 10°, respectively, than in corresponding chlorides. All values found for isolated diaryltin dihydrides correspond to compounds with no or less sterically demanding *ortho*-alkyl groups of the aryl substituents, e.g., Ph_2_SnH_2_ and Mes_2_SnH_2_. Yet, no trend between steric demand of the aryl moiety and H-Sn-H as well as C-Sn-C angles is observed.

## 3. Materials and Methods

All manipulations involving air or moisture sensitive compounds were either performed under a nitrogen atmosphere using standard Schlenk tube techniques or were carried out in a nitrogen flushed Glovebox UNILAB supplied by M. Braun. ^1^H (300.22 MHz), ^13^C (75.5 MHz), ^7^Li (116.67 MHz) as well as ^119^Sn (111.92 MHz) spectra were recorded on a Varian Mercury 300 MHz spectrometer from Varian. Spectra were referenced to solvent residual signals or with an external reference. Dried and deoxygenated solvents were obtained from an Innovative Technology solvent drying system. All other chemicals from commercial sources were used as purchased from chemical suppliers. *^i^*^Pr^Ar*NH_2_ and ^Me^Ar*NH_2_ (*^i^*^Pr^Ar* = 2,6-(Ph_2_CH)_2_-4-*i*Pr-C_6_H_2_; ^Me^Ar* = 2,6-(Ph_2_CH)_2_-4-Me-C_6_H_2_) were synthesized following procedures described in the literature [47]. Corresponding Ar*I compounds were isolated from diazotization of the aniline Ar*NH_2_ [22]. Elemental analysis was performed with an Elementar Vario MICRO cube. All IR measurements were measured fast under ambient conditions on an ALPHA-P device from Bruker in transmission mode. GC-MS measurements were carried out on an Agilent Technologies 7890A GC system coupled to an Agilent Technologies 5975 C VLMSD mass spectrometer using a HP5 column (30 m × 0.250 mm × 0.025 μm) and a carrier helium gas flow of 0.92726 mL/min. A ‘hot needle’ manual injection method at an injector temperature of 250 °C was performed. The MS conditions included positive EI ionization at ionization energy of 70 eV and a full scan mode (50–500 m/z). Melting points were determined by threefold determination with a Stuart SMP50 automatic melting point instrument.

For single crystal X-ray diffractometry, all suitable crystals were covered with a layer of silicone oil. A single crystal was selected, mounted on a glass rod on a copper pin, and placed in the cold N_2_ stream provided by an Oxford Cryosystems cryometer (T = 100 K). XRD data collection was performed on a Bruker APEX II diffractometer with use of Mo Kα radiation (λ = 0.71073 Å) from an IµS microsource and a CCD area detector. Empirical absorption corrections were applied using SADABS [48,49]. The structures were solved with use of either direct methods or the Patterson option in SHELXS. Structure refinement was carried out using SHELXL [50,51]. CIF files were edited, validated and formatted with the program OLEX2 [52]. The space group assignments and structural solutions were evaluated using PLATON [53,54]. All nonhydrogen atoms were refined anisotropically. Hydrogen atoms next to the heavy atom Sn were located on the Fourier difference map in solid state structures of **11** and **12**. However, we were not able to locate hydrogens connected to Sn atoms in the Fourier difference map for **13** and **14**. Instead, hydrogen atoms were placed using a riding model for **13** as well as **14**. All other hydrogen atoms were placed in calculated positions corresponding to standard bond lengths and angles using riding models.

### 3.1. Synthesis 

#### 3.1.1. ^iPr^Ar*I (**1**)

*^i^*^Pr^Ar*I was isolated from diazotization of the aniline *^i^*^Pr^Ar*NH_2_. [22] The crude product was recrystallized from acetone to give the product as a colorless solid.

Yield: 6.05 g (48%), colorless solid. m.p. 170-175 °C. Anal. Calcd. For C_35_H_31_I: C, 72.66; H, 5.40. Found: C, 72.66; H, 4.93. ^1^H NMR (300.22 MHz, CDCl_3_) δ 7.33-7.24 (m, 12 H; *p*/*m*-H^Ar^(Ph), overlay with solvent peak), 7.10–7.08 (d, 8 H; *o*-H^Ar^(Ph)), 6.69 (s, 2 H; *o*-H^Ar^), 6.04 (s, 2 H; 2×C*H*Ph_2_), 2.66 (septet, ^3^J_H,H_ = 6.9 Hz, 1 H; C*H*(CH_3_)_2_), 1.01 (d, ^3^J_H,H_ = 6.9 Hz, 6 H; CH(C*H*_3_)_2_) ppm. ^13^C NMR (75.5 MHz, CDCl_3_) δ 147.85 (C^Ar^), 147.16 (C^Ar^), 143.45 (C^Ar^(Ph)), 129.99 (C^Ar^(Ph)), 128.37 (C^Ar^(Ph)), 127.89 (C^Ar^), 126.46 (C^Ar^(Ph)), 108.26 (C^Ar^-I), 62.21 (*C*HPh_2_), 33.45 (*C*H(CH_3_)_2_), 23.76 (CH(*C*H_3_)_2_) ppm.

GC-MS (EI, 70 eV, toluene) t_R_ = 23.689 min, m/z: 578.2 (M^+^), 535.1 (M^+^-CH(CH_3_)_2_), 451.3 (M^+^-I).

#### 3.1.2. ^Me^Ar*I (**2**)

^Me^Ar*I was isolated from diazotization of the aniline *^i^*^Pr^Ar*NH_2_. Spectroscopic data are in accordance with literature values. [22]

#### 3.1.3. General Procedure for Ar*Li

In a Schlenk flask, aryl iodine was dissolved in toluene and cooled to −40 °C. At −40 °C, 1.1 eq of a *n*BuLi solution (1.7 M in hexanes) was added. After complete suspension, the solution was stirred at low temperatures for 1 h, while a colorless solid precipitated. The suspension was filtered, and the solid product was dried in vacuo. Crystals suitable for X-ray crystallography were obtained by recrystallization from Et_2_O at −30 °C.

##### *^i^*^Pr^Ar*Li (**3**)

Here, 8.00 g **1** (13.8 mmol, 1.0 eq) and 8.8 mL *n*BuLi solution (1.7 M in hexanes, 15.2 mmol, 1.0 eq) in 150 mL were reacted according to the general procedure to yield a yellowish solid. The filtrate was concentrated and cooled to −30 °C to give a second crop.

Yield: 4.99 g (79%), yellowish powder. m.p. > 99 °C (decomposition). ^1^H NMR (300.22 MHz, C_6_D_6_) δ 7.22–6.88 (m, 20 H; 20× *o*/*m*/*p*-H^Ar^(Ph), overlay with solvent peak), 6.82 (s, 2 H; *m*-H^Ar^), 6.05 (s, 2 H; 2×C*H*Ph_2_), 2.57 (sept, ^3^J_H,H_ = 6.9 Hz, 1 H; C*H*(CH_3_)_2_), 1.05 (d, ^3^J_H,H_ = 6.9 Hz, 6 H; CH(C*H*_3_)_2_) ppm. ^13^C NMR (75.5 MHz, C_6_D_6_) δ 157.65 (C^Ar^), 146.06 (C^Ar^(Ph)), 129.93 (C^Ar^(Ph)), 129.86 (C^Ar^), 129.10 (C^Ar^(Ph)), 126.81 (C^Ar^(Ph)), 126.50 (C^Ar^), 123.28 (C^Ar^), 63.02 (*C*HPh_2_), 34.34 (*C*H(CH_3_)_2_), 24.34 (CH(*C*H_3_)_2_) ppm. ^7^Li NMR (116.67 MHz, C_6_D_6_) δ 1.77 ppm.

##### ^Me^Ar*Li (**4**)

Here, 2.97 g **2** (5.4 mmol, 1.0 eq) and 3.5 mL *n*BuLi solution (1.7 M in hexanes, 5.9 mmol, 1.1 eq) in 50 mL were reacted according to the general procedure to yield a yellowish powder.

Yield: 1.75 g (75%), yellowish powder. m.p. >94 °C (decomposition). ^1^H NMR (300.22 MHz, C_6_D_6_) δ 7.21–6.88 (m, 20 H; *o*/*m*/*p*-H^Ar^(Ph), overlay with solvent peak), 6.78 (s, 2 H; *m*-H^Ar^), 6.08 (s, 2 H; 2×C*H*Ph_2_), 2.02 (s, 3 H; CH_3_) ppm. ^13^C NMR (75.5 MHz, C_6_D_6_) δ 158.02 (C^Ar^), 145.91 (C^Ar^(Ph)), 129.93 (C^Ar^(Ph)), 129.87 (C^Ar^), 129.13 (C^Ar^(Ph)), 126.84 (C^Ar^(Ph)), 126.50 (C^Ar^), 125.94 (C^Ar^), 62.93 (*C*HPh_2_), 21.77 (*C*H_3_) ppm. ^7^Li NMR (116.67 MHz, C_6_D_6_) δ 1.84 ppm.

#### 3.1.4. General Procedure for Ar*_2_SnCl_2_

A solution of *n*BuLi solution (1.7 M in hexanes) was added to a suspension of aryl iodine suspended in Et_2_O at −50 °C. After full conversion was detected by GC-MS, the pale yellow solution was again cooled to −50 °C, SnCl_4_ was added using a syringe. The reaction was allowed to warm up to rt overnight and the solvent was removed under reduced pressure. The resulting solid was extracted twice with DCM and the solvent was again removed under reduced pressure to yield an off-white solid. The crude product was examined by ^119^Sn NMR. For purification, the crude product was recrystallized from a mixture of DCM/*n*-pentane or DCM/toluene. 

##### *^i^*^Pr^Ar*_2_SnCl_2_ (**5**)

Here, 10.58 g **1** (18.3 mmol, 2.0 eq) in 150 mL, 12.1 mL *n*BuLi solution (1.7 M in hexanes, 20.6 mmol, 2.3 mmol) and 1.05 mL SnCl_4_ (2.34 g, 9.0 mmol, 1.0 eq) were reacted according to the general procedure. The crude product was recrystallized from DCM/n-pentane. Crystals suitable for X-ray diffraction were obtained from recrystallization in DCM/*n*-heptane at −30 °C.

Yield: 6.26 g (63%), colorless solid. m.p. 247–251 °C. Anal. Calcd. for C_70_H_62_Cl_2_Sn: C, 76.70; H, 5.49. Found: C, 75.97; H, 5.54. ^1^H NMR (300.22 MHz, CDCl_3_) δ 7.10–7.02 (m, 28 H; *p*/*m*-H^Ar^(Ph) and *m*-H^Ar^), 6.71 (d, 16 H, *o*-H^Ar^(Ph)), 6.05 (s, 4 H; 4×C*H*Ph_2_), 2.76 (sept, ^3^J_H,H_ = 6.8 Hz, 2 H; 2×C*H*(CH_3_)_2_), 1.09 (d, ^3^J_H,H_ = 6.8 Hz, 12 H; 2×CH(C*H*_3_)_2_) ppm. ^13^C NMR (75.5 MHz, CDCl_3_) δ 151.49 (C^Ar^), 149.35 (C^Ar^), 143.54 (C^Ar^(Ph)), 142.78 (C^Ar^), 129.87 (C^Ar^(Ph)), 129.21 (C^Ar^), 128.04 (C^Ar^(Ph)), 126.41 (C^Ar^), 58.11 (*C*HPh_2_), 33.91 (C*H*(CH_3_)_2_) 23.74 (CH(CH_3_)_2_) ppm. ^119^Sn NMR (111.92 MHz, CDCl_3_) δ −65.96 ppm.

##### ^Me^Ar*_2_SnCl_2_ (**6**)

Here, 2.70 g **2** (4.90 mmol, 2.0 eq) in 50 mL, 3.20 mL *n*BuLi solution (1.7 M in hexanes, 5.44 mmol, 2.3 mmol) and 0.28 mL SnCl_4_ (0.62 g, 2.40 mmol, 1.0 eq) were reacted according to the general procedure. The crude product was recrystallized from DCM/toluene. Crystals suitable for X-ray diffraction were obtained from recrystallization in DCM/*n*-heptane at rt.

Yield: 0.90 g (36%), colorless solid. m.p. 253–255 °C. Anal. Calcd. for C_66_H_54_Cl_2_Sn: C, 76.46; H, 5.25. Found: C, 77.29; H, 5.65. ^1^H NMR (300.22 MHz, CDCl_3_) δ 7.12–7.01 (m, 24 H; *p*/*m*-H^Ar^(Ph)), 6.91 (s, ^4^J_H,Sn_ = 36.7 Hz, 4 H; *m*-H^Ar^), 6.70 (d, 16 H; *o*-H^Ar^(Ph)), 6.04 (s, 4 H; 4×C*H*Ph_2_), 2.23 (s, 6 H; 2×C*H*_3_) ppm. ^13^C NMR (75.5 MHz, CDCl_3_) δ 149.33 (C^Ar^), 143.34 (C^Ar^(Ph)), 142.54 (C^Ar^), 140.89 (C^Ar^), 131.75 (C^Ar^), 129.87 (C^Ar^(Ph)), 128.06 (C^Ar^(Ph)), 126.42 (C^Ar^(Ph)), 58.09 (*C*HPh_2_) 21.88 (*C*H_3_) ppm. ^119^Sn NMR (111.92 MHz, CDCl_3_) δ −64.65 ppm.

#### 3.1.5. ^iPr^Ar*SnMe_3_ (**7**)

At −50 °C, 1.00 mL of a *n*BuLi solution (1.7 M in hexanes, 1.7 mmol, 1.1 eq) was added to a suspension of 0.91 g **1** (1.6 mmol, 1.0 eq) in 50 mL Et_2_O. After full conversion was detected by GC-MS, 315 mg Me_3_SnCl (1.6 mmol, 1.0 eq) were added. The reaction was allowed to warm up to rt, upon which a yellow solid was formed. For workup, the solvent was removed under reduced pressure and the gained solid was extracted with DCM (2 × 15 mL). The solvent was again removed under reduced pressure to give the pure product, which was used without further purification. Crystals suitable for single crystal X-ray diffraction were obtained by slow solvent evaporation from a solution in DCM.

Yield: 0.740 g (76%), yellow solid. m.p. 170–175 °C. ^1^H NMR (300.22 MHz, CDCl_3_) δ 7.31–7.19 (m, 12 H; *m*/*p*-H^Ar^(Ph), overlay with solvent peak), 7.02 (d, ^3^J_H,H =_ 7.0 Hz, 8 H; *o*-H^Ar^(Ph)), 6.69 (s, ^4^J_H,H_ = 16 Hz, 2 H; *m*-H^Ar^), 5.92 (s, 2 H; 2×C*H*Ph_2_), 2.64 (sept, ^3^J_H,H_ = 6.9 Hz, 1 H; C*H*(CH_3_)_2_), 1.00 (d, ^3^J_H,H_ = 6.9 Hz, 6 H; CH(C*H*_3_)_2_), 0.02 (s, ^2^J_H,Sn_ = 52 Hz, 9 H; Sn(C*H*_3_)_3_) ppm. ^13^C NMR (75.5 MHz, CDCl_3_) δ 150.85 (^2^J_C,Sn_ = 31 Hz, C^Ar^), 148.62 (^4^J_C,Sn_ = 9 Hz, C^Ar^), 144.93 (C^Ar^(Ph)), 142.15 (C^Ar^), 130.08 (C^Ar^(Ph)), 128.19 (C^Ar^(Ph)), 126.71 (C^Ar^), 126.32 (C^Ar^(Ph)), 57.85 (^3^J_C,Sn_ = 30 Hz*, C*HPh_2_), 33.73 (*C*H(CH_3_)_2_), 23.77 (CH(*C*H_3_)_2_), -4.17 (^1^J_C,117Sn_ = 326 Hz, ^1^J_C,119Sn_ = 341 Hz; Sn(*C*H_3_)_3_) ppm. ^119^Sn NMR (111.92 MHz, CDCl_3_) δ −56.80 ppm.

#### 3.1.6. ^iPr^Ar*SnCl_2_Me (**8**)

In a 50 mL Schlenk flask 620 mg **7** (1.0 mmol, 1.0 eq) was mixed neat with 0.15 mL SnCl_4_ (335 mg, 1.3 mmol, 1.3 eq) and slowly heated up to 130 °C. The reaction was kept at 130 °C for 14 h. For workup the solid was extracted with DCM (6 mL), filtered and the solvent was removed under reduced pressure. The crude product was recrystallized from DCM/*n*-pentane to give the compound in quantitative amounts. Crystals suitable for X-ray crystallography were obtained by recrystallization from DCM/*n-*heptane at rt. 

Yield: 660 mg (quant.), colorless solid. m.p. 169–171 °C. ^1^H NMR (300.22 MHz, CDCl_3_) δ 7.33–7.22 (m, 12 H; *m*/*p-*H^Ar^(Ph), overlay with solvent peak), 7.04 (d, ^3^J = 6.9 Hz, 8 H; *o*-H^Ar^(Ph)), 6.76 (s, ^4^J = 10.5 Hz, 2 H; *m*-H^Ar^), 6.63 (s, ^4^J = 36.8 Hz, 2 H; 2×C*H*Ph_2_), 2.76–2.54 (sept, ^3^J_H,H_ = 6.8 Hz, 1 H; C*H*(CH_3_)_2_), 0.97 (d, ^3^J_H,H_ = 6.8 Hz, 6 H; CH(C*H*_3_)_2_), -0.41 (s, ^2^J_H,Sn_ = 74.5 Hz, 3 H; SnCl_2_(C*H*_3_)) ppm. ^13^C NMR (75.5 MHz, CDCl_3_) δ 151.55 (C^Ar^), 151.28 (C^Ar^), 143.28 (C^Ar^(Ph)), 130.83 (C^Ar^(Ph)), 130.27 (C^Ar^), 128.68 (C^Ar^(Ph)), 127.48 (C^Ar^), 127.19 (C^Ar^(Ph)), 55.74 (^3^J_C,Sn_ = 39.4 Hz; *C*HPh_2_), 33.93 (*C*H(CH_3_)_2_), 23.58 (CH(CH_3_)_2_), 6.20 (SnCl_2_(*C*H_3_)) ppm. ^119^Sn NMR (111.92 MHz, CDCl_3_) δ 15.07 ppm.

#### 3.1.7. General Procedure for Ar*SnI_3_

A solution of *t*BuLi (1.7 M in hexanes) was added to a suspension of aryl iodine suspended in Et_2_O at −50 °C. After full conversion was detected by GC-MS, the pale yellow solution was again cooled to −50 °C and added in portions to a suspension of SnCl_2_ in Et_2_O also cooled to −50 °C. After complete addition, the reaction was allowed to warm up to rt overnight. At 0 °C, I_2_ was added to the suspension upon which the suspension turned brownish. The reaction was allowed to warm up to rt and the color faded slowly to an intensive yellow. Volatiles were removed under reduced pressure and the resulting solid was extracted twice with DCM. The solvent was again removed under reduced pressure to yield an off-white solid. The crude product was examined by ^119^Sn NMR. For purification, the crude product was recrystallized from a mixture of toluene/*n*-pentane or toluene.

##### *^i^*^Pr^Ar*SnI_3_ (**9**)

Here, 5.00 g **1** (8.64 mmol, 1.0 eq), 11.2 mL *t*BuLi solution (1.7 M in hexanes, 19.0 mmol, 2.2 eq), 1.80 g SnCl_2_ (9.49 mmol, 1.1 eq) and 2.41 g I_2_ (9.49 mmol, 1.1 eq) were reacted according to the general procedure. The crude product was recrystallized from toluene/*n*-pentane. Crystals suitable for X-ray diffraction were obtained from recrystallization in toluene/*n*-heptane at −30 °C.

Yield: 5.05 g (68%), yellow crystals. m.p. 181–183 °C. Anal. Calcd. for C_35_H_31_I_3_Sn: C, 44.20; H, 3.29. Found: C, 43.37; H, 3.12. ^1^H NMR (300.22 MHz, CDCl_3_) δ 7.32–7.24 (m, 12 H; *p*/*m*-H^Ar^(Ph), overlay with solvent peak), 7.05 (d, ^3^J_H,H_ = 6.6 Hz, 8 H; *o-*H^Ar^(Ph)), 6.93 (s, ^4^J_H,Sn_ = 14.7 Hz, 2 H; *m*-H^Ar^), 6.69 (s, ^4^J_H,Sn_ = 52.8 Hz, 2 H; 2×C*H*Ph_2_), 2.73-2.64 (m, 1 H; C*H*(CH_3_)_2_), 0.99 (d, ^3^J_H,H_ = 6.9 Hz, 6 H; CH(C*H*_3_)_2_) ppm. ^13^C NMR (75.5 MHz, CDCl_3_) δ 152.33 (C^Ar^), 149.94 (^2^J_C,Sn_ = 66.7 Hz; C^Ar^), 143.05 (C^Ar^(Ph)), 130.29 (C^Ar^(Ph)), 129.10 (C^Ar^), 129.01 (C^Ar^), 128.56 (C^Ar^(Ph)), 127.01 (C^Ar^(Ph)), 55.45 (^3^J_C,Sn_ = 54.1 Hz; *C*HPh_2_), 33.81 (*C*H(CH_3_)_2_), 23.56 (CH(*C*H_3_)_2_) ppm. ^119^Sn NMR (111.92 MHz, CDCl_3_) δ −937.27 ppm.

##### ^Me^Ar*SnI_3_ (**10**)

Here, 4.08 g **2** (7.40 mmol, 1.0 eq), 9.6 mL *t*BuLi solution (1.7 M in hexanes, 16.3 mmol, 2.2 eq), 1.55 g SnCl_2_ (8.16 mmol, 1.1 eq) and 2.07 g I_2_ (8.16 mmol, 1.1 eq) were reacted according to the general procedure. The crude product was recrystallized from hot toluene. Crystals suitable for X-ray diffraction were obtained from recrystallization in DCM/*n*-heptane at −30 °C.

Yield: 3.68 g (53%), yellow crystals. m.p. 251–255 °C. Anal. Calcd. for C_33_H_27_I_3_Sn: C, 42.94; H, 2.95. Found: C, 43.29; H, 2.83. ^1^H NMR (300.22 MHz, CDCl_3_) δ 7.34–7.24 (m, 12 H; *m*/*p*-H^Ar^(Ph), overlay with solvent peak), 7.08 (d, ^3^J_H,H_ = 7.6 Hz, 8 H; *o*-H^Ar^(Ph)), 6.93 (s, 2 H; *m*-H^Ar^), 6.67 (s, 2 H; 2×C*H*Ph_2_), 2.21 (s, 3 H; CH_3_) ppm. ^13^C NMR (75.5 MHz, CDCl_3_) δ 149.80 (C^Ar^), 142.96 (C^Ar^(Ph)), 141.84 (C^Ar^), 131.52 (C^Ar^), 130.31 (C^Ar^(Ph)), 128.81 (C^Ar^), 128.58 (C^Ar^(Ph)), 127.03 (C^Ar^(Ph)), 55.34 (*C*HPh_2_), 21.78 (*C*H_3_) ppm. ^119^Sn NMR (111.92 MHz, CDCl_3_) δ −939.57 ppm.

#### 3.1.8. General Procedure for Ar*_2_SnH_2_

In a Schlenk flask, LiAlH_4_ was suspended in a mixture of Et_2_O/toluene (4/1) and cooled down to 0 °C. At 0 °C, diaryltin dichloride was added. After 2 h of stirring at 0 °C, the reaction mixture was transferred via a cannula onto degassed H_2_SO_4_ (0.5 M, cooled to 0 °C), while anaerobic conditions were maintained. After separation of the layers, the organic phase was washed once with degassed, saturated potassium tartrate solution (75 mL) to remove aluminum salts, dried over Na_2_SO_4_ and filtered. The solvent was removed to give colorless crude reaction products.

##### *^i^*^Pr^Ar*_2_SnH_2_ (**11**)

Here, 82 mg LiAlH_4_ (2.16 mmol, 1.2 eq) and 2.00 g **5** (1.83 mmol, 1.0 eq) were reacted according to the general procedure. The colorless crude product was recrystallized from toluene/n-pentane. Crystals suitable for X-ray crystallography were also obtained from a mixture of toluene/n-pentane at −30 °C.

Yield: 930 mg (50%), colorless crystals. m.p. 231–237 °C (decomposition at T > 250 °C). ^1^H NMR (300.22 MHz, C_6_D_6_) δ 7.11–6.96 (m, 44 H; *o*/*m*/*p*-H^Ar^(Ph) and *m*-H^Ar^), 6.28 (s, 4 H; 4×C*H*Ph_2_), 5.55 (s, ^1^J_H,117Sn_ = 1942 Hz, ^1^J_H,119Sn_ = 2033 Hz, 2 H; SnH), 2.51 (sept, ^3^J_H,H_ = 6.8 Hz, 2 H; 2×C*H*(CH_3_)_2_), 0.99 (d, ^3^J_H,H_ = 6.8 Hz, 12 H; 2×CH(C*H*_3_)_2_) ppm. ^13^C NMR (75.5 MHz, C_6_D_6_) δ 151.67 (C^Ar^), 149.84 (C^Ar^), 144.85 (C^Ar^(Ph)), 142.37 (C^Ar^), 130.47 (C^Ar^(Ph)), 128.51 (C^Ar^(Ph)), 127.45 (C^Ar^), 126.61 (C^Ar^(Ph)), 60.11 (*C*HPh_2_), 34.21 (*C*H(CH_3_)_2_), 23.98 (CH(*C*H_3_)_2_) ppm. ^119^Sn NMR (111.92 MHz, C_6_D_6_) δ −331.30 (^1^J_Sn,1H_ = 2033 Hz) ppm. ATR-FTIR 1885 (s; ν_s_ SnH) cm^−^^1^.

##### ^Me^Ar*_2_SnH_2_ (**12**)

Here, 88 mg LiAlH_4_ (2.32 mmol, 0.8 eq) and 3.00 g **6** (2.89 mmol, 1.0 eq) were reacted according to the general procedure. The colorless crude product was recrystallized from toluene/n-pentane or toluene/Et_2_O. Crystals suitable for X-ray crystallography were obtained from toluene at −30 °C.

Yield: 1.50 g (54%), colorless crystals. m.p. 207–211 °C (decomposition at T > 215 °C). ^1^H NMR (300.22 MHz, C_6_D_6_) δ 7.07–6.95 (m, 44 H; *o*/*m*/*p*-H^Ar^(Ph) and *m*-H^Ar^), 6.28 (s, 4 H; 4×C*H*Ph_2_), 5.60 (s, ^1^J_H,117Sn_ = 1930 Hz, ^1^J_H,119Sn_ = 2019 Hz, 2 H; Sn*H*), 1.87 (s, 6 H; 2×C*H*_3_) ppm. ^13^C NMR (75.5 MHz, C_6_D_6_) δ 151.78 (C^Ar^), 144.70 (C^Ar^(Ph)), 141.40 (C^Ar^), 139.08 (C^Ar^), 130.45 (C^Ar^(Ph)), 130.16 (C^Ar^), 128.56 (C^Ar^(Ph)), 126.61 (C^Ar^(Ph)), 60.18 (*C*HPh_2_), 21.44 (*C*H_3_) ppm. ^119^Sn NMR (111.92 MHz, C_6_D_6_) δ −331.51 (^1^J_Sn,1H_ = 2019 Hz) ppm. ATR-FTIR 1886 (s; ν_s_ SnH) cm^−^^1^.

#### 3.1.9. General Procedure for Ar*SnH_3_

In a round-bottom Schlenk flask, aryltin triiodide was dissolved in toluene to give a yellow solution and cooled to 0 °C. At 0 °C, a solution of DIBAL-H (1.0 M in toluene) was added, upon which the solution turned slowly colorless. After 1 h of stirring at 0 °C, the solvent was removed under reduced pressure to give a colorless oil. The oil was extracted three times with *n*-pentane or Et_2_O, while being cooled to 0 °C, and the remaining colorless product was dried in vacuo.

##### iPrAr*SnH_3_ (**13**)

Here, 2.00 g **9** (2.10 mmol, 1.0 eq) and 6.9 mL DIBAL-H solution (1.0 M in toluene, 6.90 mmol, 3.3 eq) were reacted according to the general procedure. The *n*-pentane was concentrated to give a second crop. Crystals suitable for X-ray crystallography were obtained by recrystallization from *n*-pentane at −30 °C.

Yield: 650 mg (54%), colorless solid. m.p. > 105 °C (decomposition). ^1^H NMR (300.22 MHz, C_6_D_6_) δ 7.11–6.98 (m, 22 H; *o*/*m*/*p*-H^Ar^(Ph) and *m*-H^Ar^), 5.99 (s, 2 H; 2×C*H*Ph_2_), 4.82 (s, ^1^J_H,117Sn_ = 1843 Hz, ^1^J_H,119Sn_ = 1930 Hz, 3 H; Sn*H*), 2.44 (sept, ^3^J_H,H_ = 6.8 Hz, 1 H; C*H*(CH_3_)_2_), 0.92 (d, ^3^J_H,H_ = 6.8 Hz, 6 H; CH(C*H*_3_)_2_) ppm. ^13^C NMR (75.5 MHz, C_6_D_6_) δ 152.15 (C^Ar^), 149.63 (C^Ar^), 144.39 (C^Ar^(Ph)), 137.00 (C^Ar^), 130.64 (C^Ar^(Ph)), 128.69 (C^Ar^(Ph)), 126.94 (C^Ar^), 126.73 (C^Ar^(Ph)), 60.60 (*C*HPh_2_), 34.12 (*C*H(CH_3_)_2_), 23.86 (CH(*C*H_3_)_2_) ppm. ^119^Sn NMR (111.92 MHz, C_6_D_6_) δ −407.06 (^1^J_Sn,1H_ = 1930 Hz) ppm. ATR-FTIR 1848 (s; ν_s_ SnH) cm^−^^1^.

##### ^Me^Ar*SnH_3_ (**14**)

Here, 2.00 g **10** (2.10 mmol, 1.0 eq) and 6.7 mL DIBAL-H solution (1.0 M in toluene, 6.70 mmol, 3.1 eq) were reacted according to the general procedure. The Et_2_O was concentrated to give a second crop. Crystals suitable for X-ray crystallography were obtained by recrystallization from toluene at rt.

Yield: 667 mg (56%), colorless crystals. m.p. > 110 °C (decomposition). ^1^H NMR (300.22 MHz, C_6_D_6_) δ 7.10–6.99 (m, 20 H; *o*/*m*/*p*-H^Ar^(Ph)), 6.92 (s, ^4^J_H,Sn_ = 19.8 Hz, 2 H; *m*-H^Ar^) 5.98 (s, 2 H; 2×C*H*Ph_2_), 4.83 (s, s, ^1^J_H,117Sn_ = 1845 Hz, ^1^J_H,119Sn_ = 1931 Hz, 3 H; Sn*H*), 1.82 (s, 3 H; 2×C*H*_3_) ppm. ^13^C NMR (75.5 MHz, C_6_D_6_)k δ 151.74 (C^Ar^), 143.88 (C^Ar^(Ph)), 138.49 (C^Ar^), 136.09 (C^Ar^), 130.21 (C^Ar^(Ph)), 129.10 (C^Ar^), 128.29(C^Ar^(Ph)), 126.31 (C^Ar^(Ph)), 60.09 (*C*HPh_2_), 20.95 (*C*H_3_) ppm. ^119^Sn NMR (111.92 MHz, C_6_D_6_) δ −406.67 (^1^J_Sn,1H_ = 1931 Hz) ppm. ATR-FTIR 1854 (s; ν_s_ SnH) cm^−^^1^.

## 4. Conclusions

The sterically encumbered tin halides Ar*_2_SnCl_2_ and Ar*SnI_3_ as well as corresponding tin hydrides Ar*_2_SnH_2_ and Ar*SnH_3_ featuring the bulky ligand backbone Ar* (*^i^*^Pr^Ar* = 2,6-(CHPh_2_)_2_-4-*i*PrC_6_H_2_; ^Me^Ar* = 2,6-(CHPh_2_)_2_-4-MeC_6_H_2_) were prepared and characterized by multinuclear (^1^H, ^13^C, ^119^Sn) NMR as well as IR spectroscopy. Additionally, solid state structures of all reported compounds were authenticated by single crystal X-ray diffraction. Isolated aryltin trihydrides possess a surprisingly increased thermal stability and oxygen tolerance compared to less sterically encumbered organotin hydrides. Due to their less labile but still functional nature, isolated aryltin hydrides are likely to undergo follow-up chemistry. Their intriguing reactivities are currently examined.

## Figures and Tables

**Table 1:** Sn NMR shifts (ppm) of aryltin compounds and ^119^Sn NMR shifts (ppm) and coupling constants (Hz) of isolated aryltin hydrides.

	^119^Sn NMR (ppm)	^119^Sn NMR (ppm)	^119^Sn NMR (ppm)	^1^J (^1^H, ^117/119^Sn) (Hz)
	**X = Cl**	**X = I**	**X = H**	
^iPr^Ar*_2_SnX_2_	−65.96	−498.22 *	−331.30	1942/2033
^Me^Ar*_2_SnX_2_	−64.65	−496.98 *	−331.51	1930/2019
^iPr^Ar*SnMe_3_	−56.80	-	-	-
^iPr^Ar*SnMeX_2_	+15.07	-	-	-
^iPr^Ar*SnX_3_	-	−937.27	−407.06	1843/1930
^Me^Ar*SnX_3_	-	−939.57	−406.67	1845/1931

Signals marked with * were only found in crude products.

**Table 2:** Selected bond lengths (Å) and angles (°) of aryltin compounds **5**, **6**, **7**, **8**, **9** as well as **10** (X = Cl, I).

	Sn-C (Å)	Sn-X (Å)	C-Sn-C (°)	X-Sn-X (°)
^iPr^Ar*_2_SnCl_2_ (**5**)	2.1501(15)	2.3781(5)	125.77(7)	94.49(3)
^Me^Ar*_2_SnCl_2_ (**6**)	2.155(5), 2.159(6)	2.402(2), 2.344(2)	119.5(2)	95.82(5)
^iPr^Ar*SnMe_3_ (**7**)	2.189(1)	-	113.04(6) 111.75(6) 114.81(6)	-
^iPr^Ar*SnMeCl_2_ (**8**)	2.141(7) 2.140(6)	2.373(2), 2.375(2), 2.369(2) 2.381(2)	131.2(3) 130.0(3)	92.47(7)
^iPr^Ar*SnI_3_ (**9**)	2.161(2)	2.7130(4), 2.6964(4), 2.6721(4)	-	97.31(1), 106.04(1), 107.66(1)
^Me^Ar*SnI_3_ (**10**)	2.158(3)	2.6994(4), 2.6752(4)	-	105.743(11), 95.665(12)

**Table 3:** Selected bond lengths (Å) and angles (°) of diaryltin dihydride compounds **11** and **12**.

	Sn-C (Å)	Sn-H (Å)	C-Sn-C (°)	H-Sn-H (°)
*^i^*^Pr^Ar*_2_SnH_2_ (**11**)	2.187(3), 2.171(3)	1.71(3), 1.70(4)	105.9(1)	109(2)
^Me^Ar*_2_SnH_2_ (**12**)	2.188(2), 2.186(2)	1.79(2), 1.80(3)	109.49(8)	100.7(9)

## Supplementary Materials

The following are available online. ^119^Sn NMR of crude products in the synthesis of **5**, **6**, **9** and **10** (Figure S1-S5). NMR spectra (^1^H, ^13^C and if applicable ^7^Li and ^119^Sn) of all unknown isolated compounds (**1**, **3**-**14**) (Figure S6-S47). ATR-IR of isolated tin hydrides **11**, **12**, **13** and **14** (Figure S48-S51) and Raman spectra of aryltin iodides **9** and **10** (Figure S53-S54). Tabulated crystallographic data and ORTEP plots for compounds **1**, **3**-**14**. CCDC 1983429 (for **1**), 1983430 (for **3**), 1983431 (for **4**), 1983432 (for **7**), 1983433 (for **6**), 1983434 (for **11**), 1983435 (for **8**), 1983436 (for **5**), 1983437 (for **14**), 1983438 (for **13**), 1983439 (for **12**), 1983440 (for **10**) and 1983441 (for **9**) contain the supplementary crystallographic data for this paper. These data can be obtained free of charge from The Cambridge Crystallographic Data Centre.
