# Supplementary material for: Diaryltin Dihydrides and Aryltin Trihydrides with Intriguing Stability
Source: Molecules. 2020 Feb 27;25(5):1076. doi: 10.3390/molecules25051076 (PMC7179233; doi:10.3390/molecules25051076)
Supplement: Supplementary file 1 [file molecules-25-01076-s001.pdf]

molecules

Supporting Experimental Information

Diaryltin Dihydrides and Aryltin Trihydrides with Intriguing Stability

Beate G. Steller *, Berenike Doler and Roland C. Fischer

Graz University of Technology, Stremayrgasse 9/V, 8010 Graz, Austria; b.doler@student.tugraz.at(B.D.); roland.fischer@tugraz.at(R.C.F.)
* Correspondence: beate.steller@tugraz.at

Table of Contents

1 NMR spectra 2
2 ATR-IR spectra 26
3 Raman Spectra 28
4 Crystal Structures and crystallographic tables 29

1 NMR spectra

1.1 NMR spectra of crude products

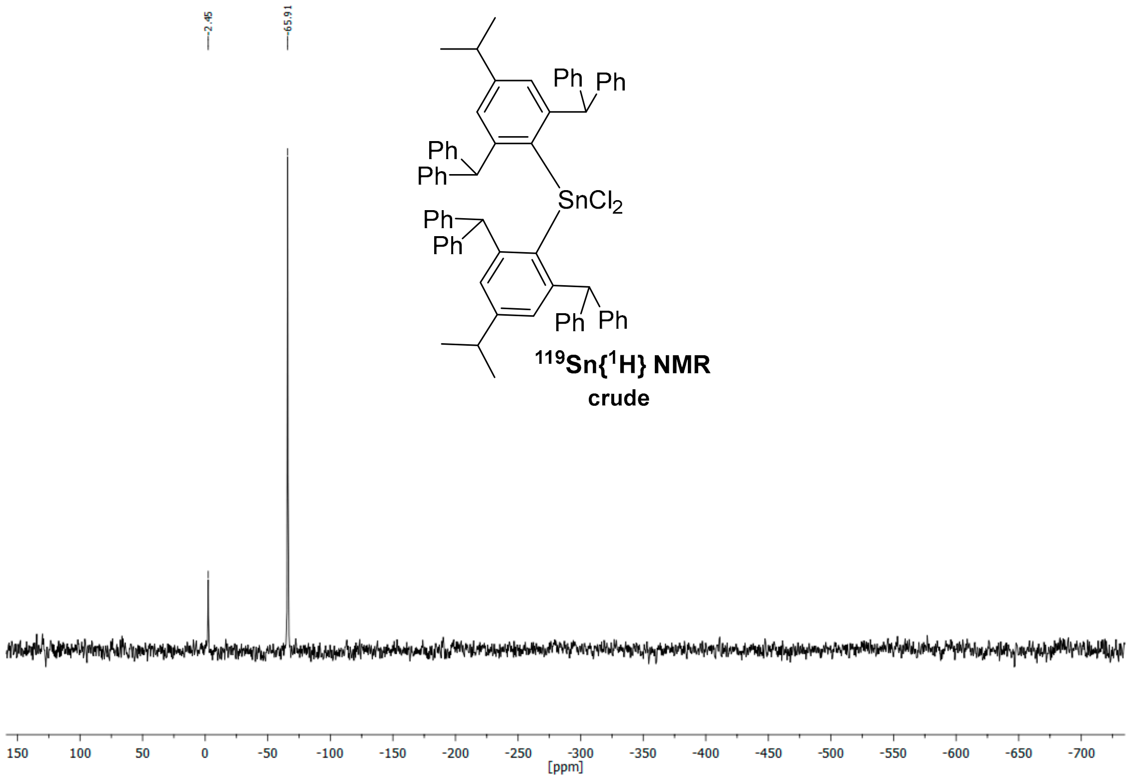

Figure S 1 $^{119}Sn\{^{1}H\}$ NMR of crude product $^{iPr}Ar^{*}_{2}SnCl_2$.

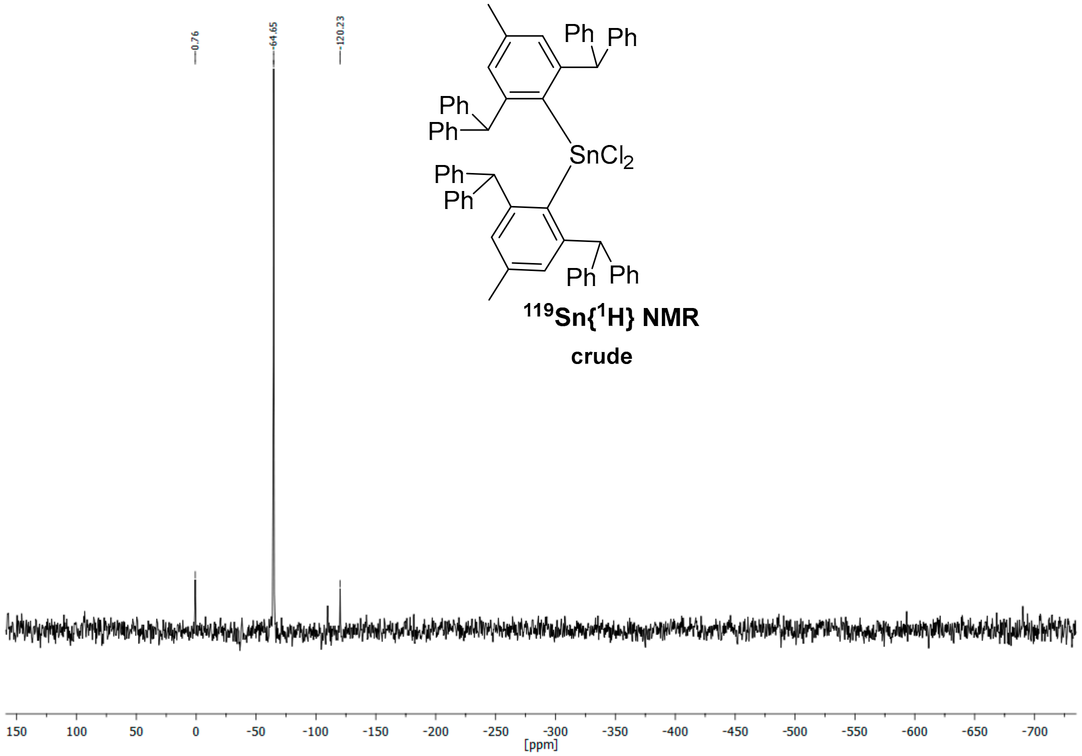

Figure S 2 $^{119}Sn\{^{1}H\}$ NMR of crude product $^{Me}Ar^{*}_{2}SnCl_2$.

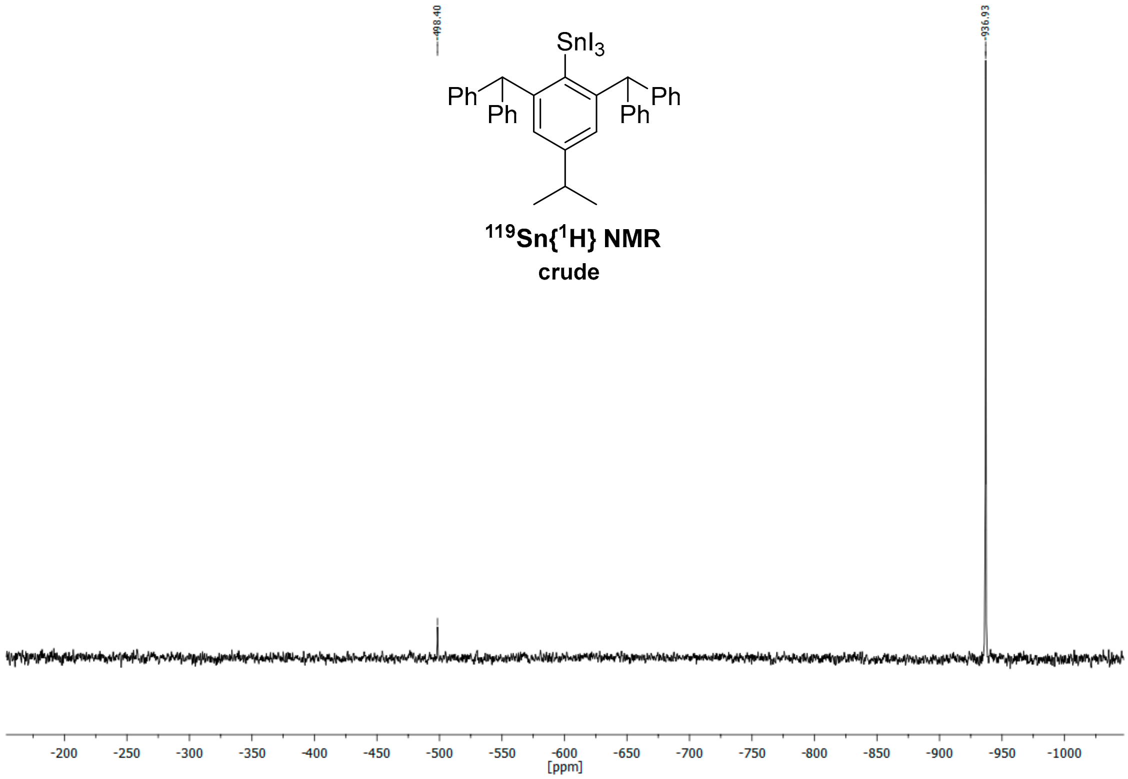

Figure S 3 $^{119}Sn\{^1H\}$ NMR of crude product $^{iPr}Ar^*SnI_3$.

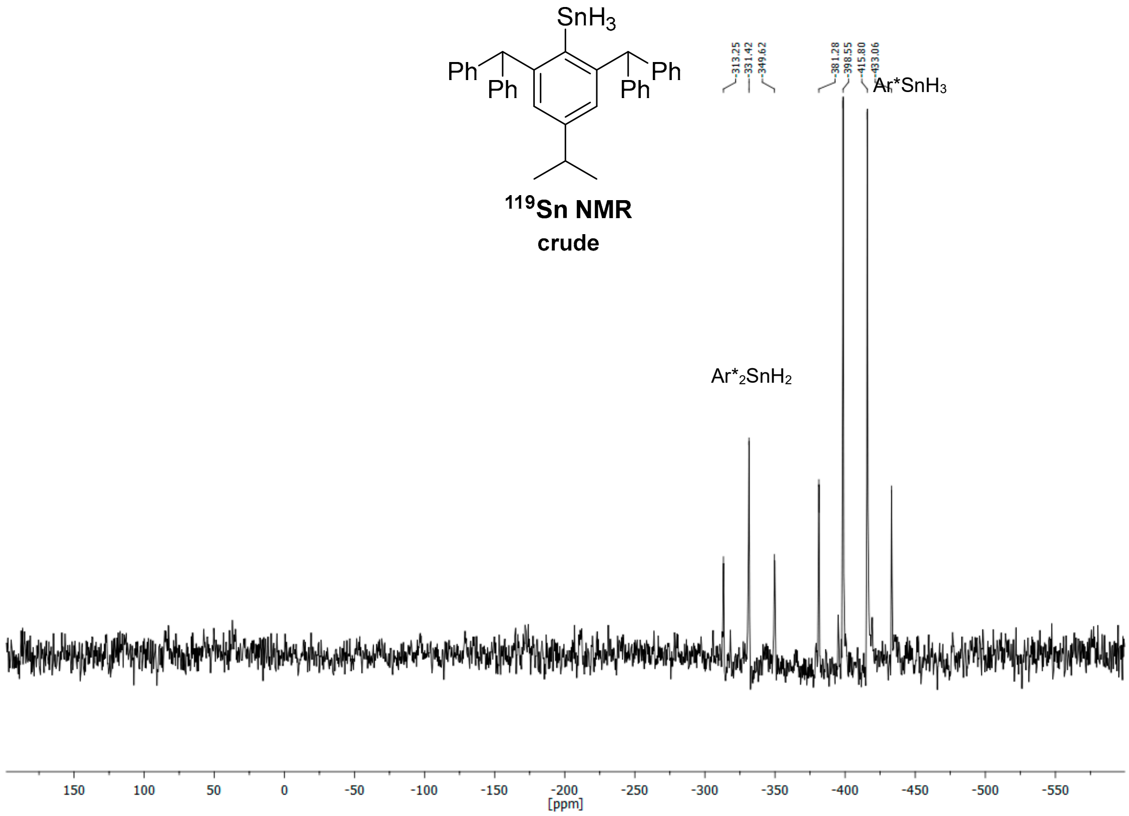

Figure S 4 ^{119}Sn NMR of crude product $^{iPr}Ar^*SnI_3$ after hydration with $LiAlH_4$.

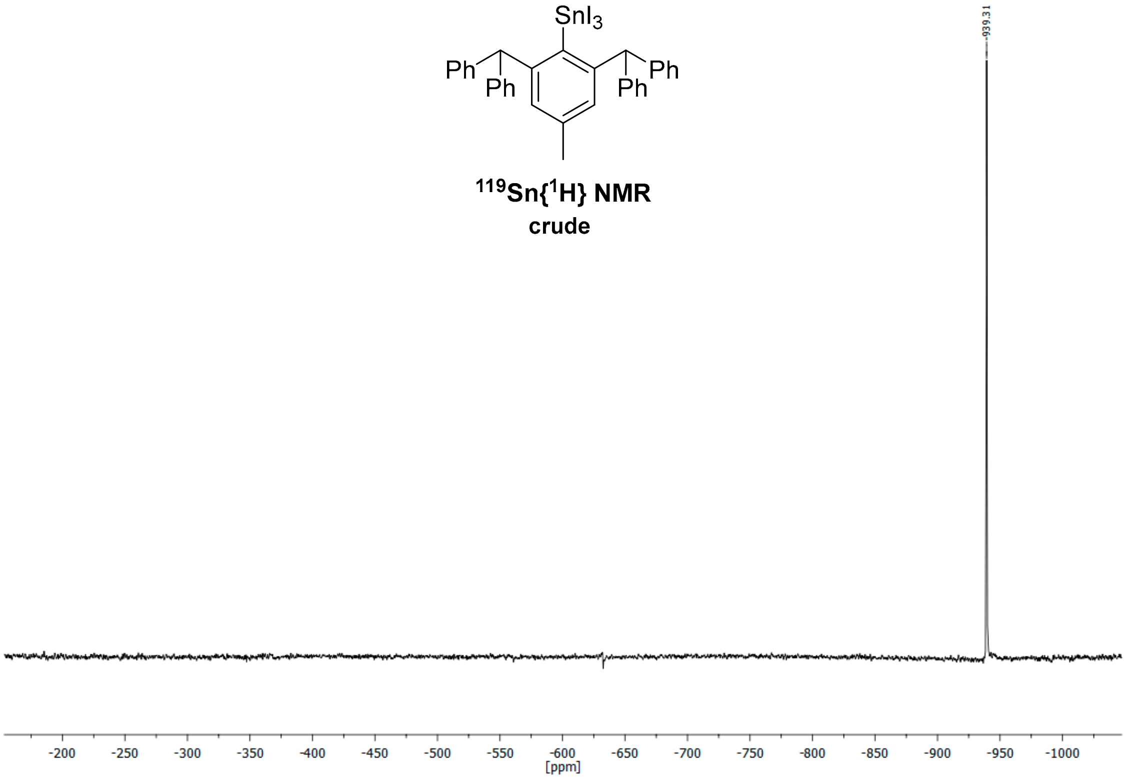

Figure S 5 $^{119}Sn\{^1H\}$ NMR of crude product $^{Me}Ar^*SnI_3$.

1.2 NMR spectra of isolated compounds

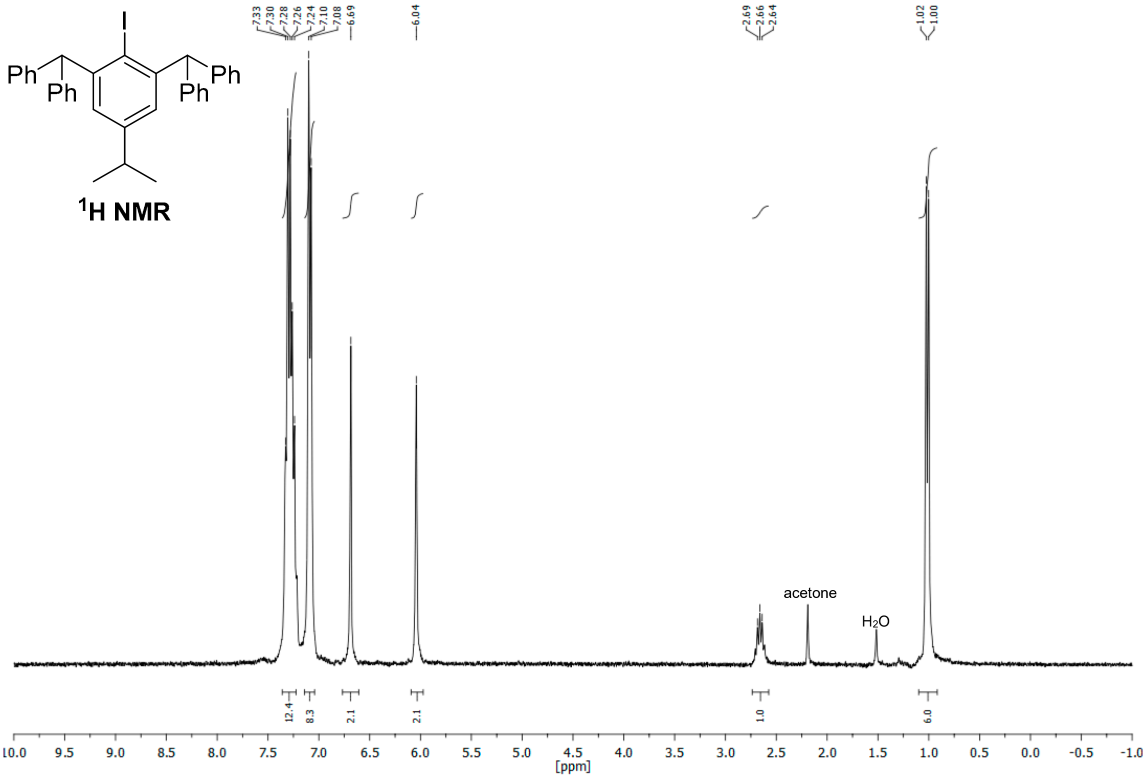

Figure S 6 ^{1}H NMR of $^{i\mathrm{Pr}}$Ar*I (**1**).

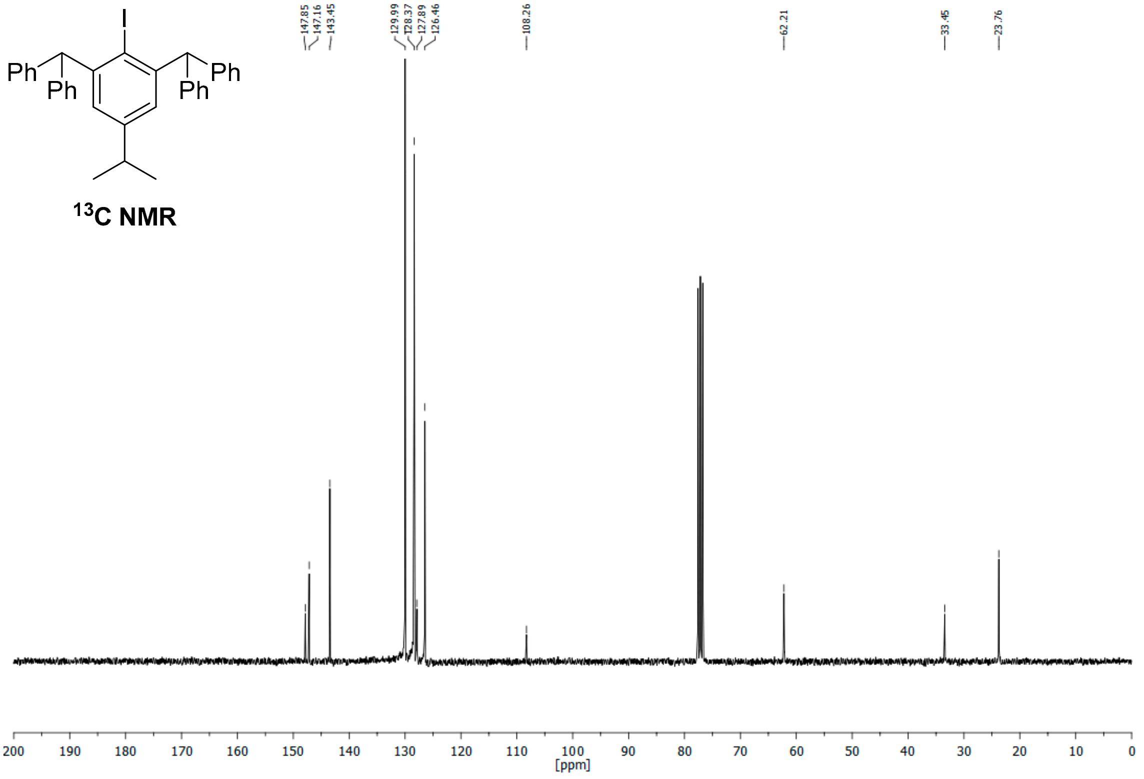

Figure S 7 ^{13}C NMR of $^{i\mathrm{Pr}}$Ar*I (**1**).

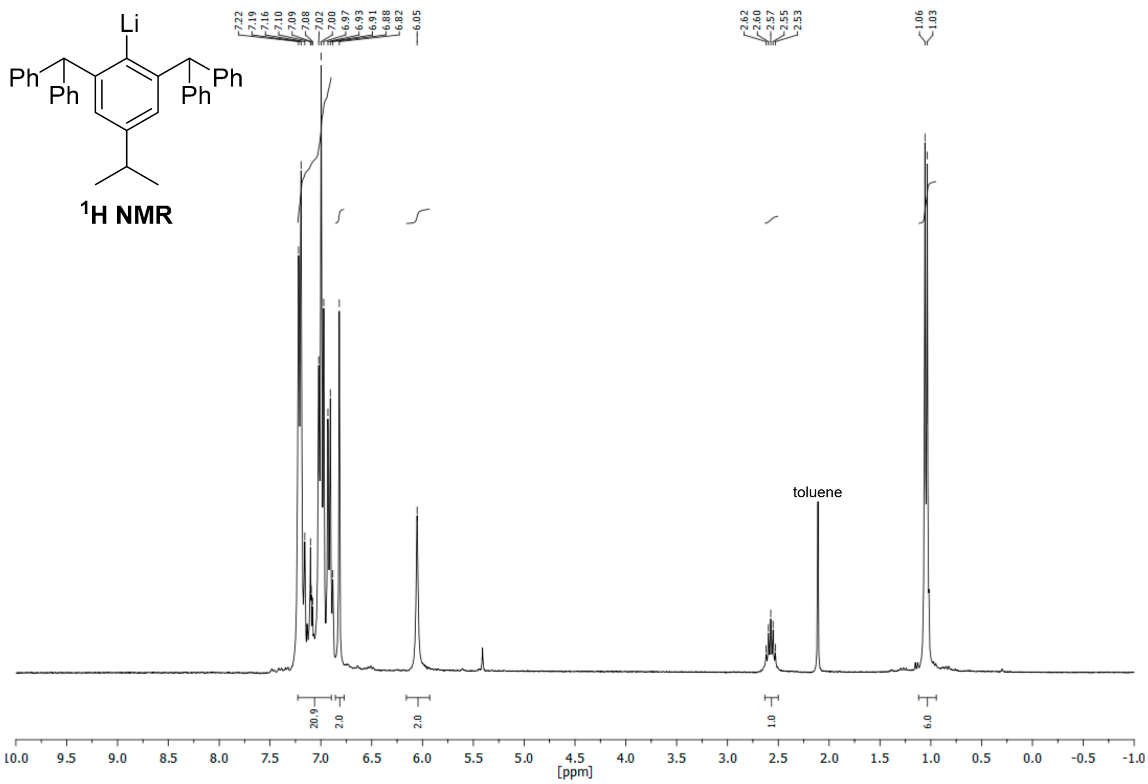

Figure S 8 ^{1}H NMR of ^{iPr}Ar*Li (**3**).

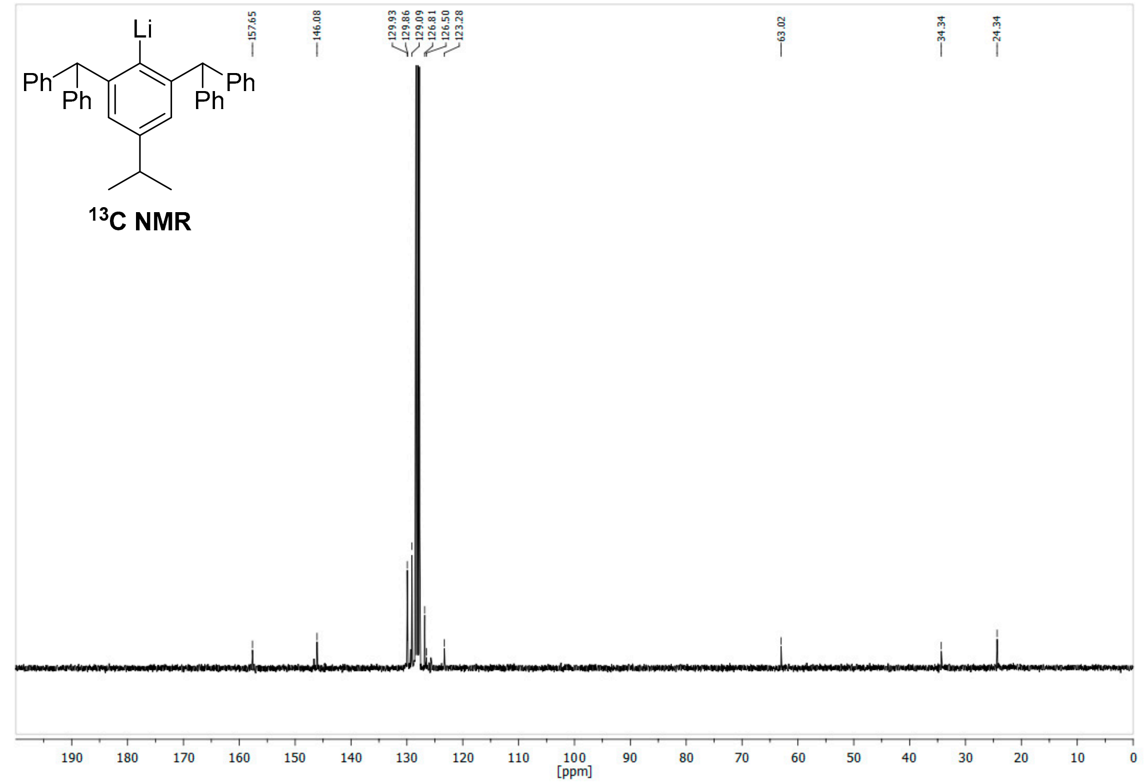

Figure S 9 ^{13}C NMR of ^{iPr}Ar*Li (**3**).

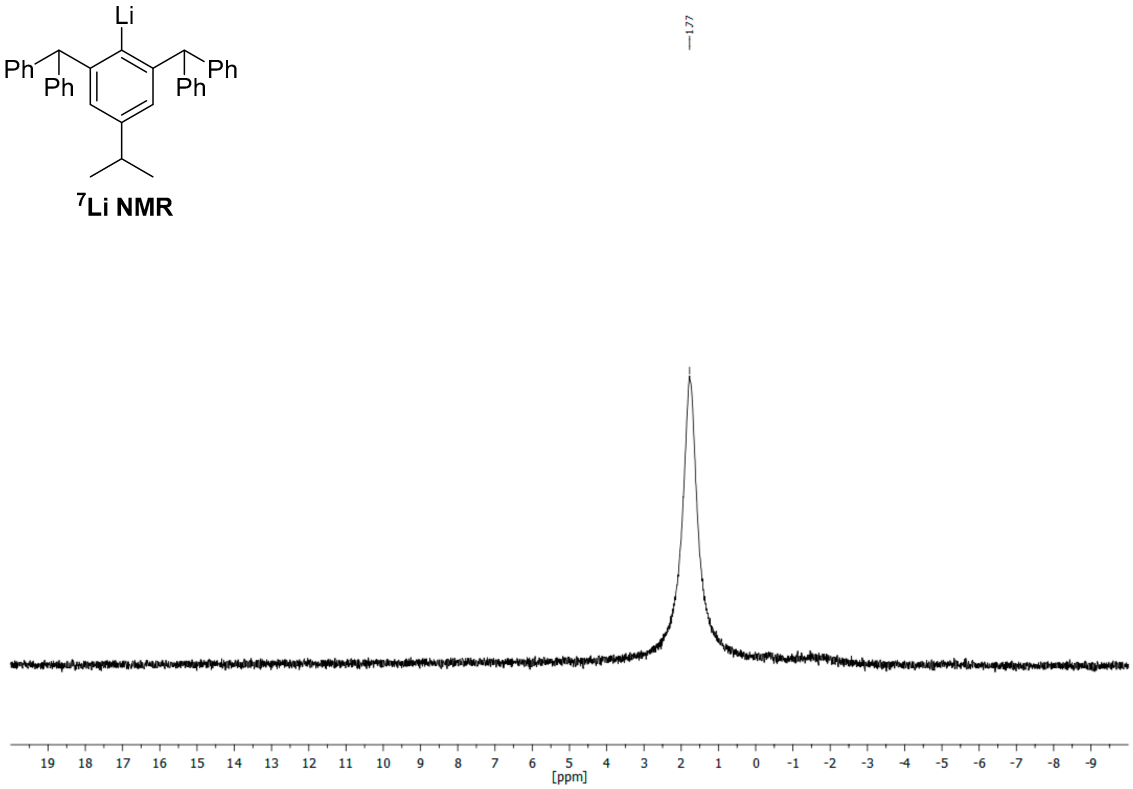

Figure S 10 ^{7}Li NMR of ^{iPr}Ar*Li (**3**).

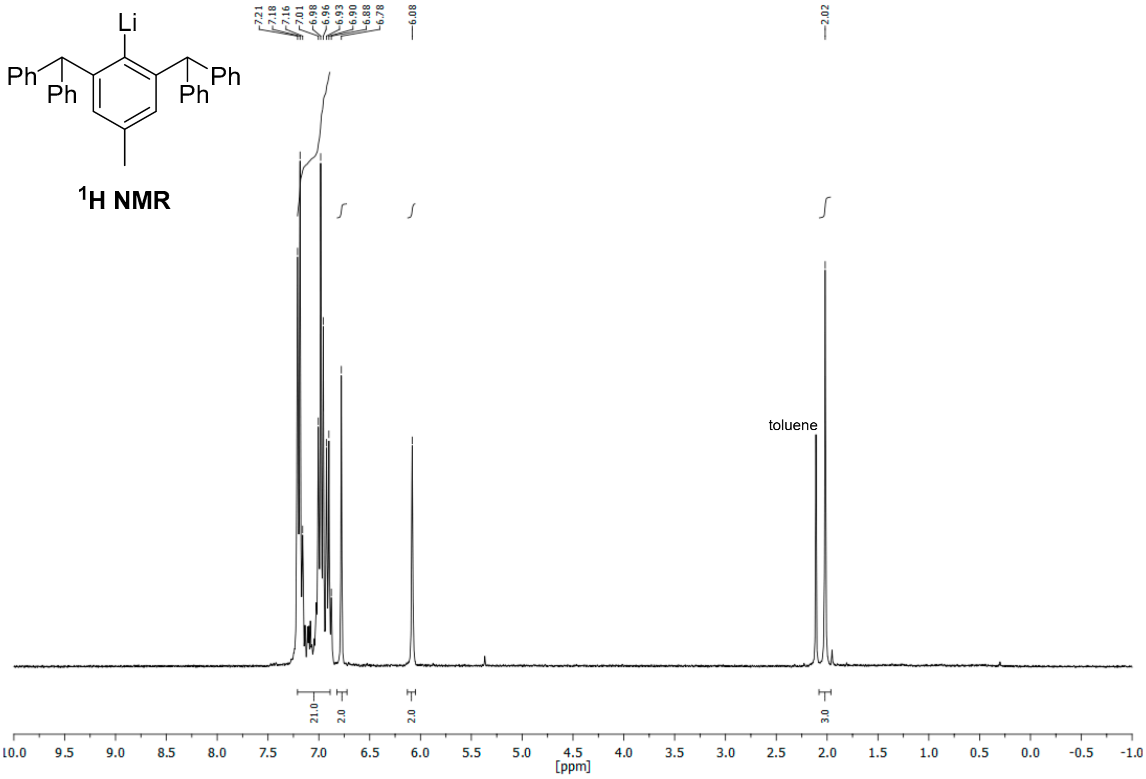

Figure S 11 ^{1}H NMR of ^{Me}Ar*Li (**4**).

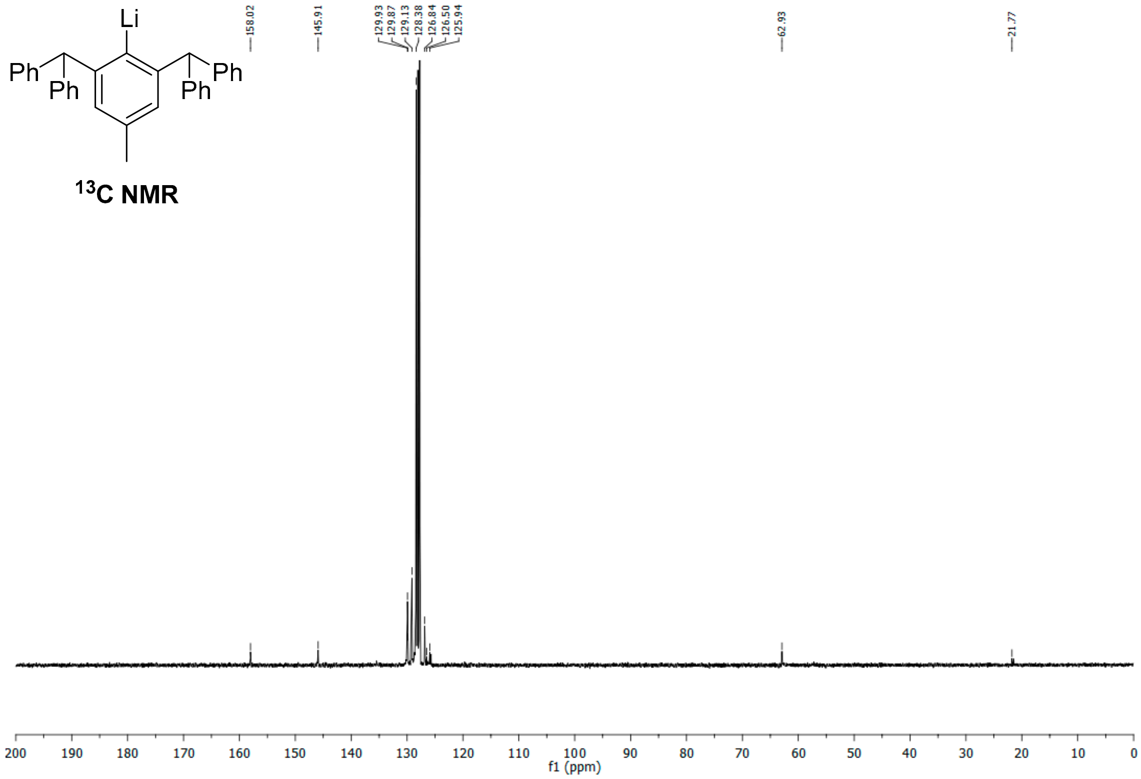

Figure S 12 ^{13}C NMR of ^{Me}Ar*Li (**4**).

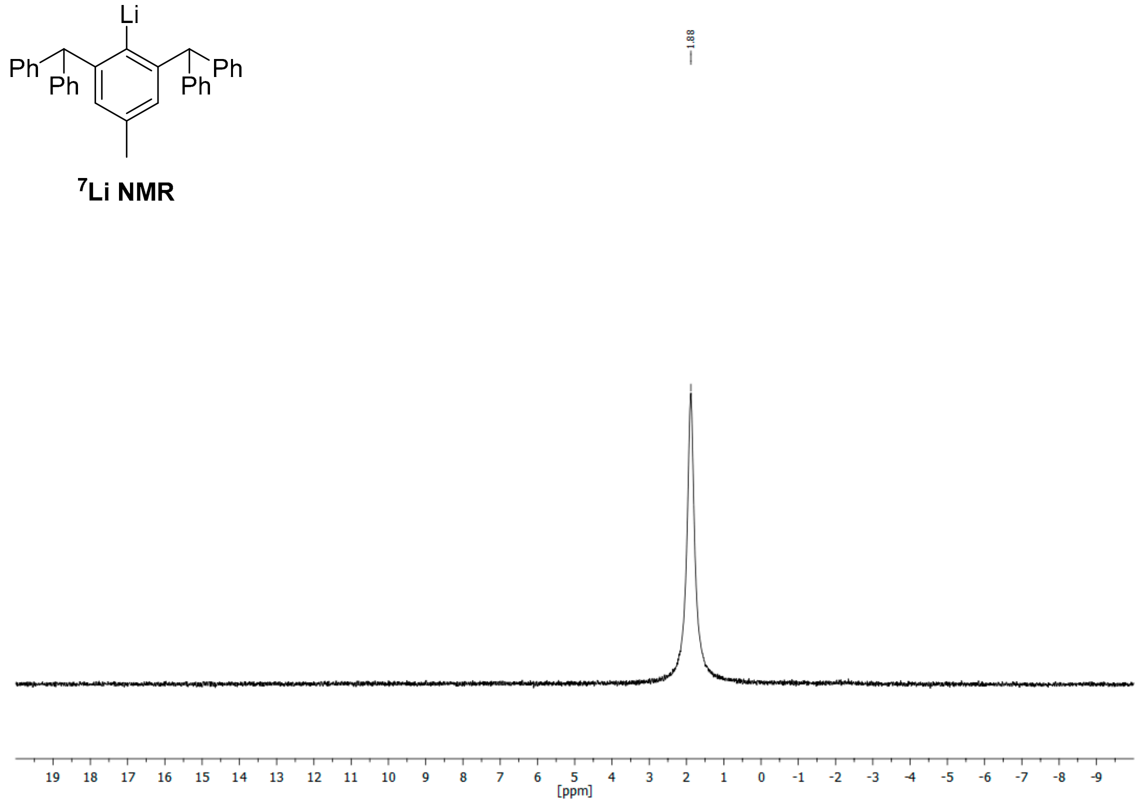

Figure S 13 ^{7}Li NMR of ^{Me}Ar*Li (**4**).

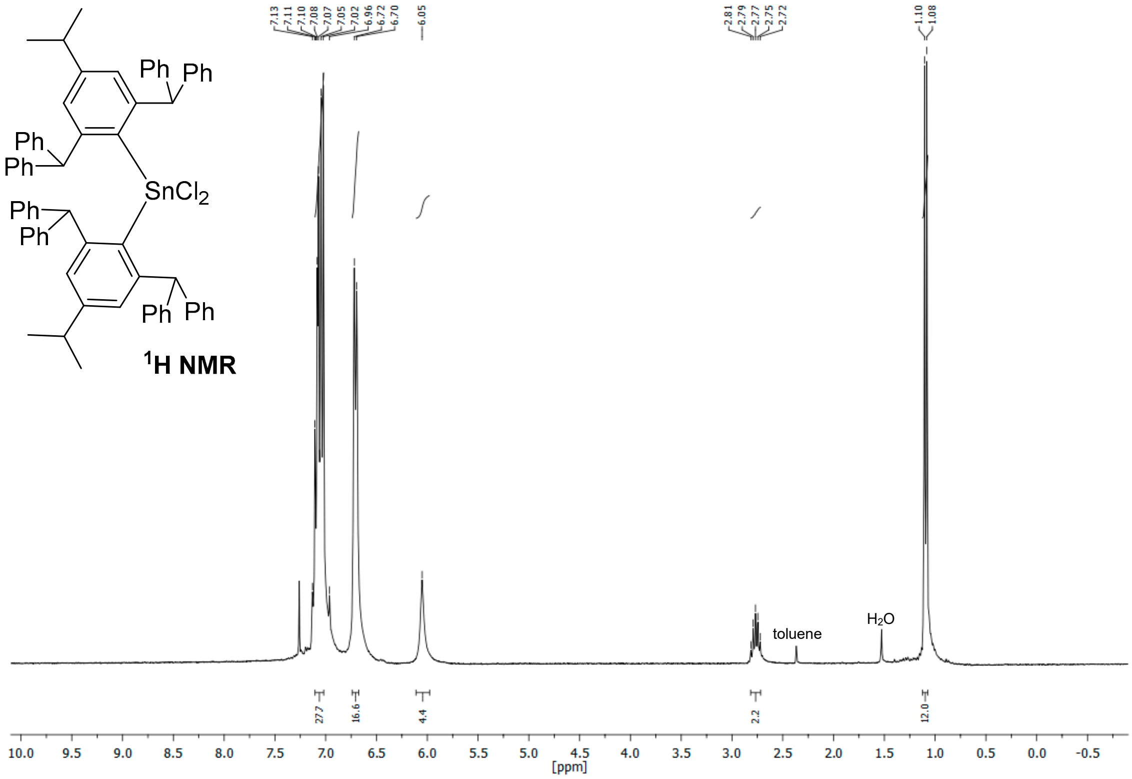

Figure S 14 ^{1}H NMR of ^{iPr}Ar*$_2$SnCl$_2$ (**5**).

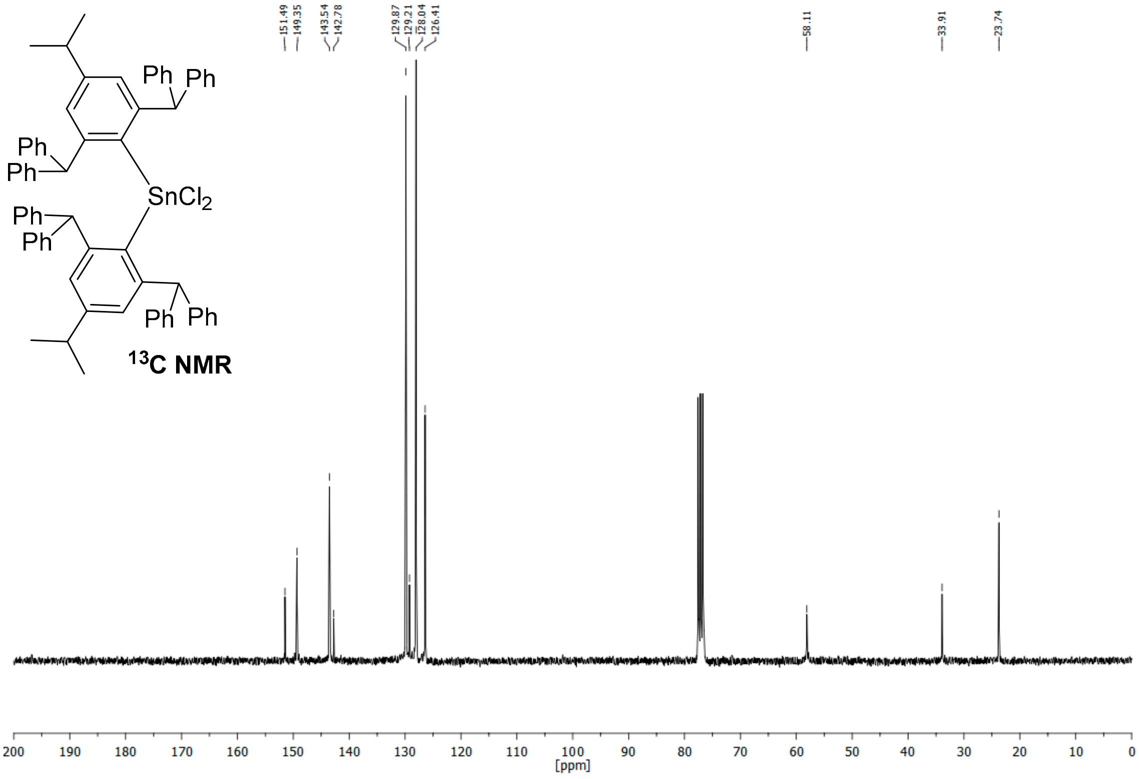

Figure S 15 ^{13}C NMR of ^{iPr}Ar*$_2$SnCl$_2$ (**5**).

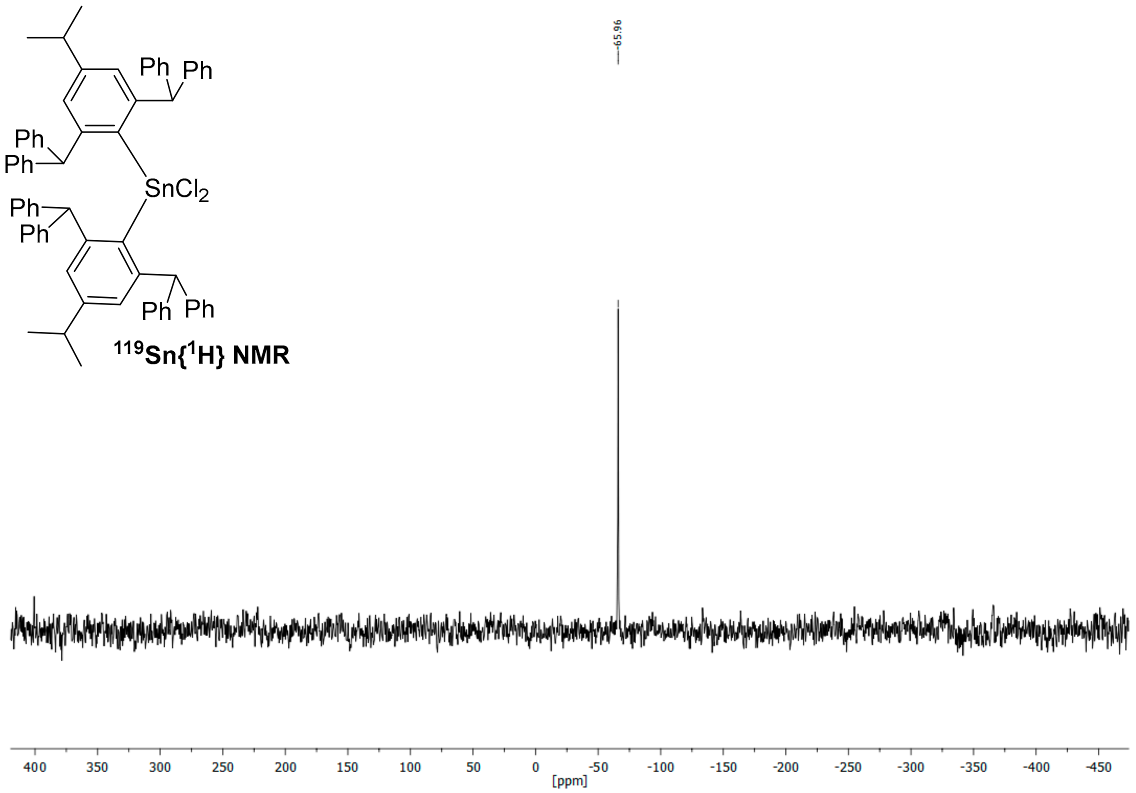

Figure S 16 ^{119}Sn NMR of ^{iPr}Ar*$_2$SnCl$_2$ (**5**).

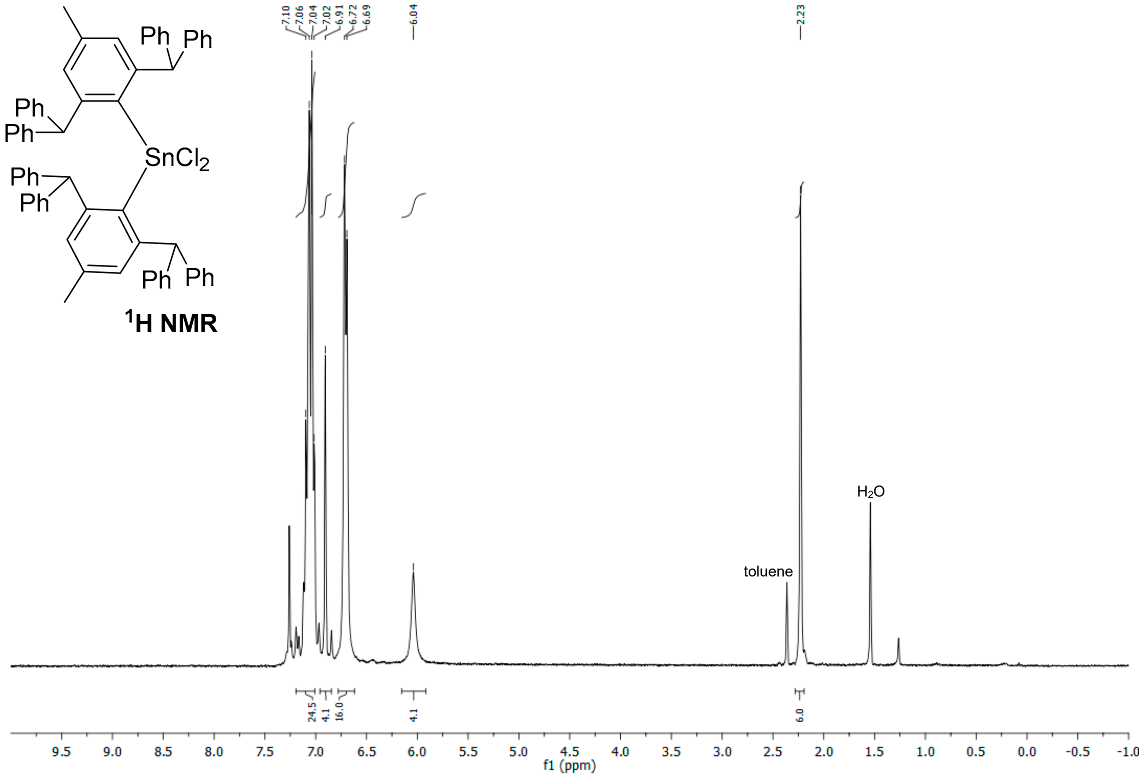

Figure S 17 ^{1}H NMR of ^{Me}Ar*$_2$SnCl$_2$ (**6**).

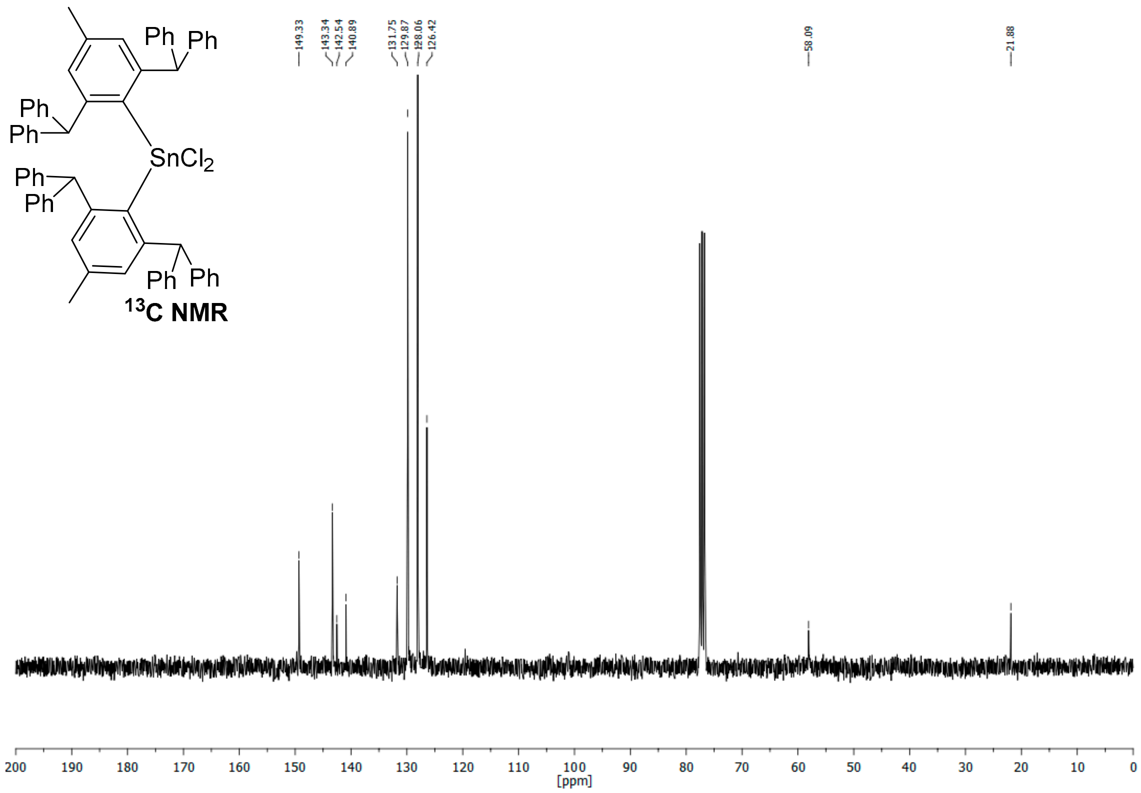

Figure S 18 ^{13}C NMR of $^{Me}Ar^*{}_2SnCl_2$ (**6**).

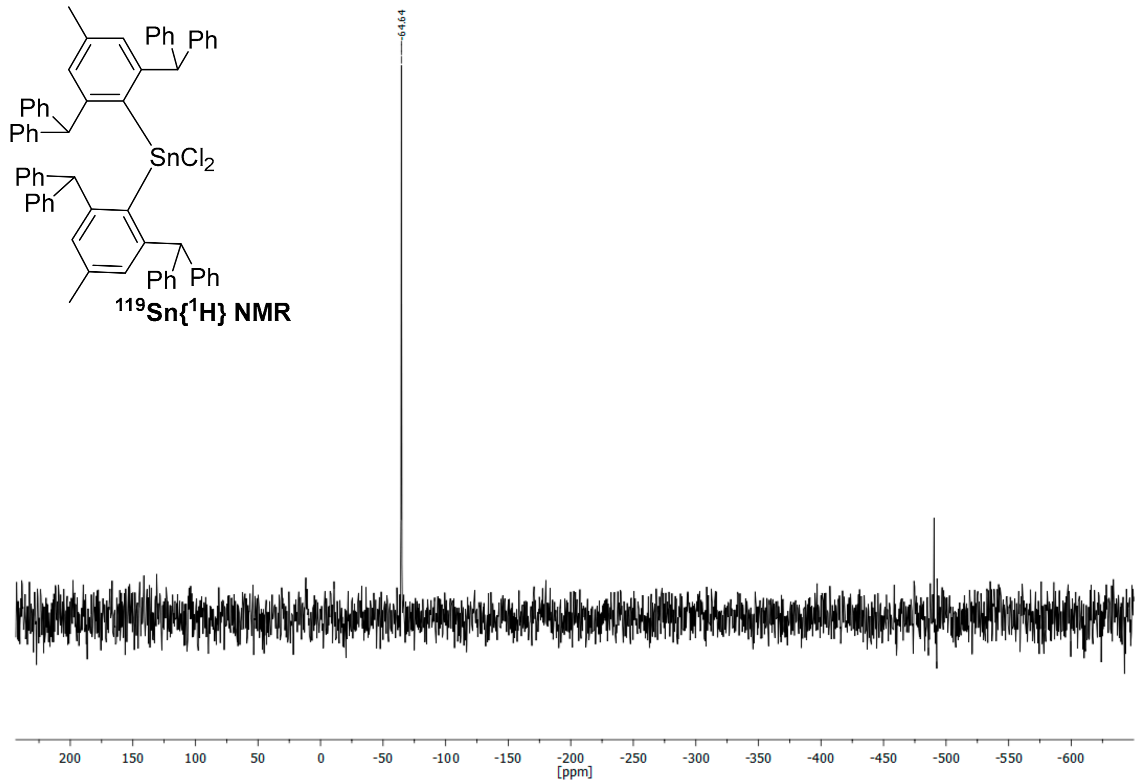

Figure S 19 ^{119}Sn{^{1}H} NMR of $^{Me}Ar^*{}_2SnCl_2$(**6**).

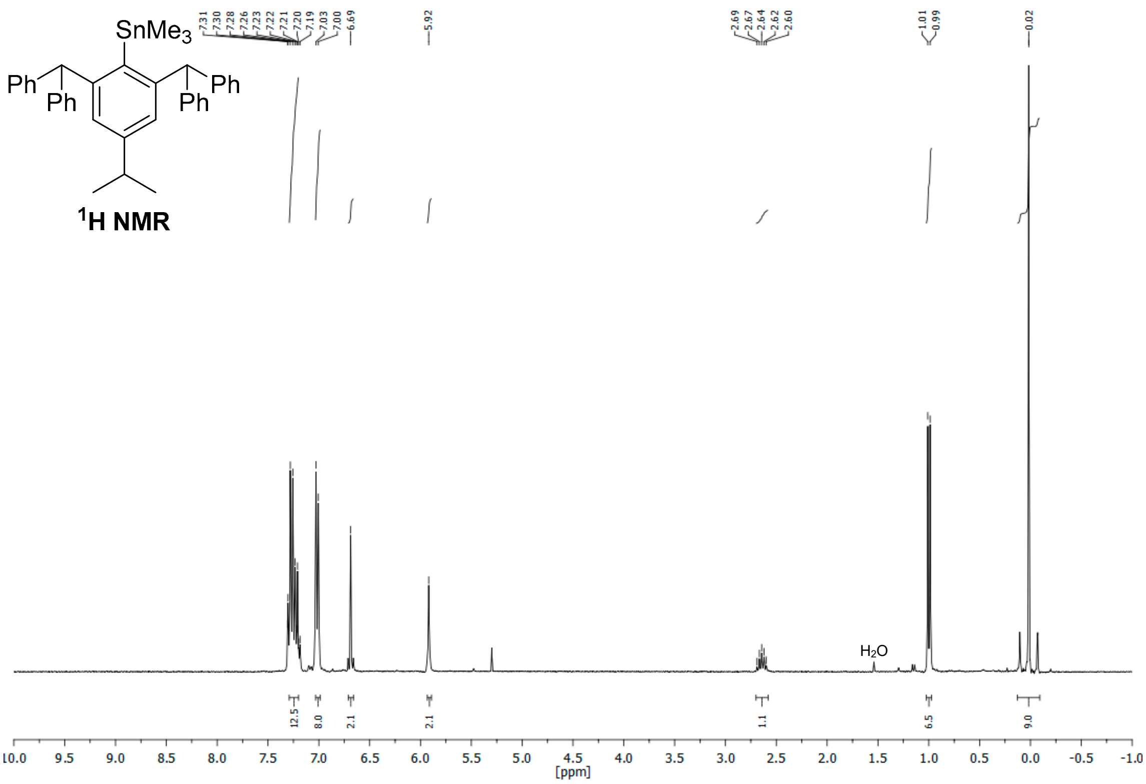

Figure S 20 ^{1}H NMR of ^{iPr}Ar*SnMe3 (**7**).

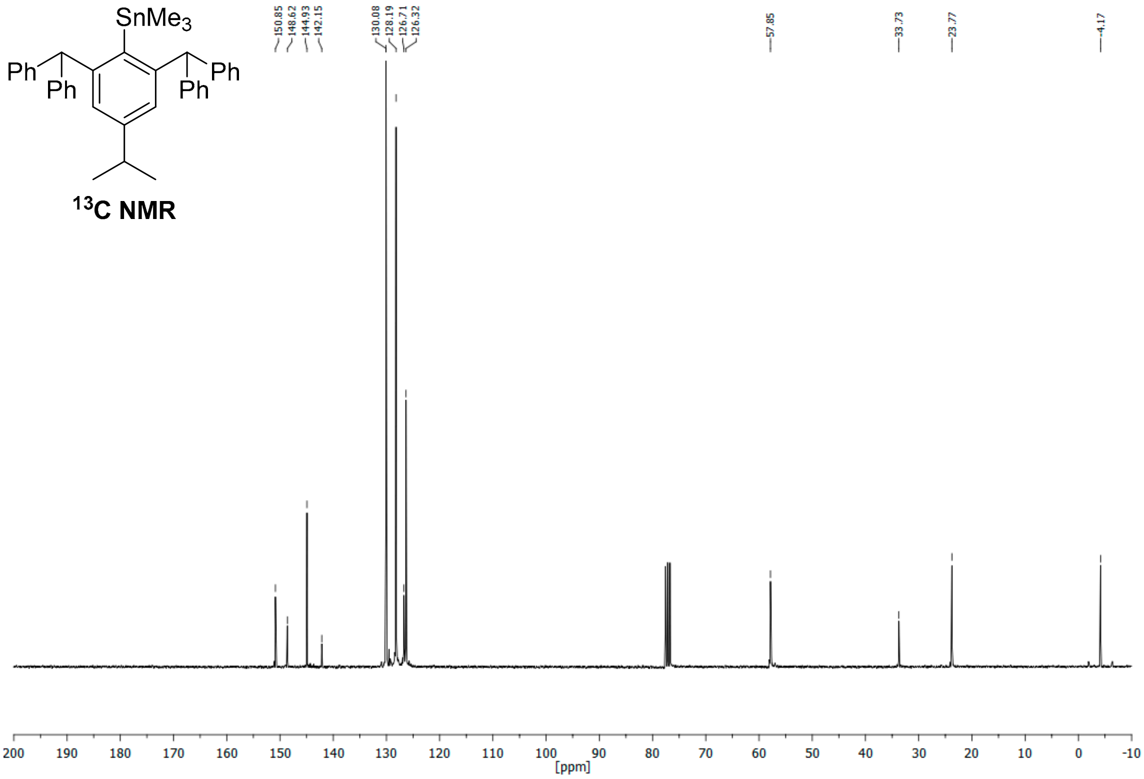

Figure S 21 ^{13}C NMR of ^{iPr}Ar*SnMe3 (**7**).

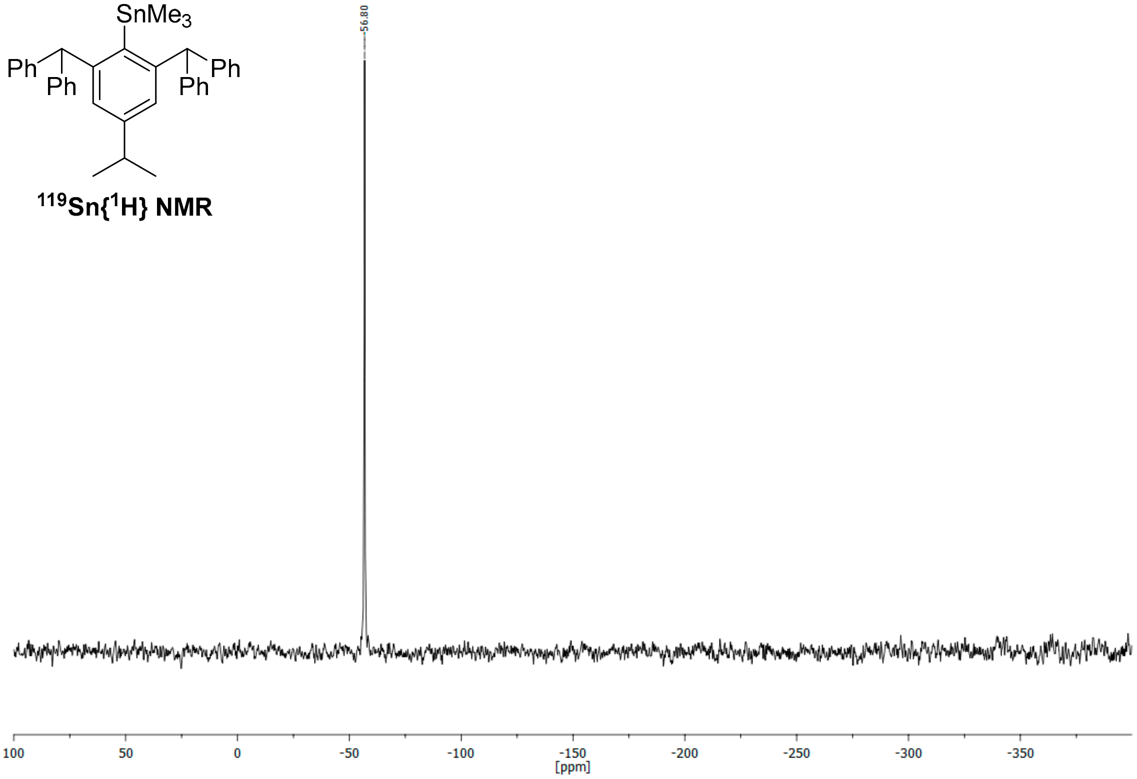

Figure S 22 ^{119}Sn NMR of ^{iPr}Ar*SnMe$_3$ (**7**).

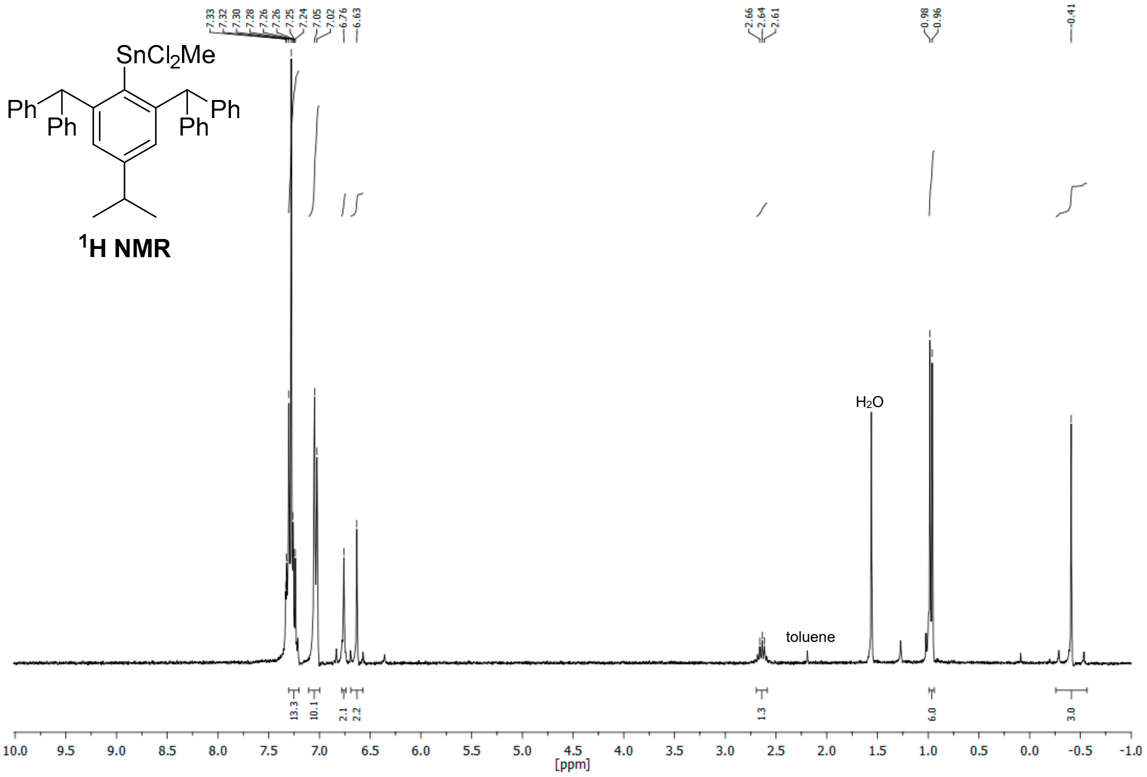

Figure S 23 ^{1}H NMR of ^{iPr}Ar*SnCl$_2$Me (**8**).

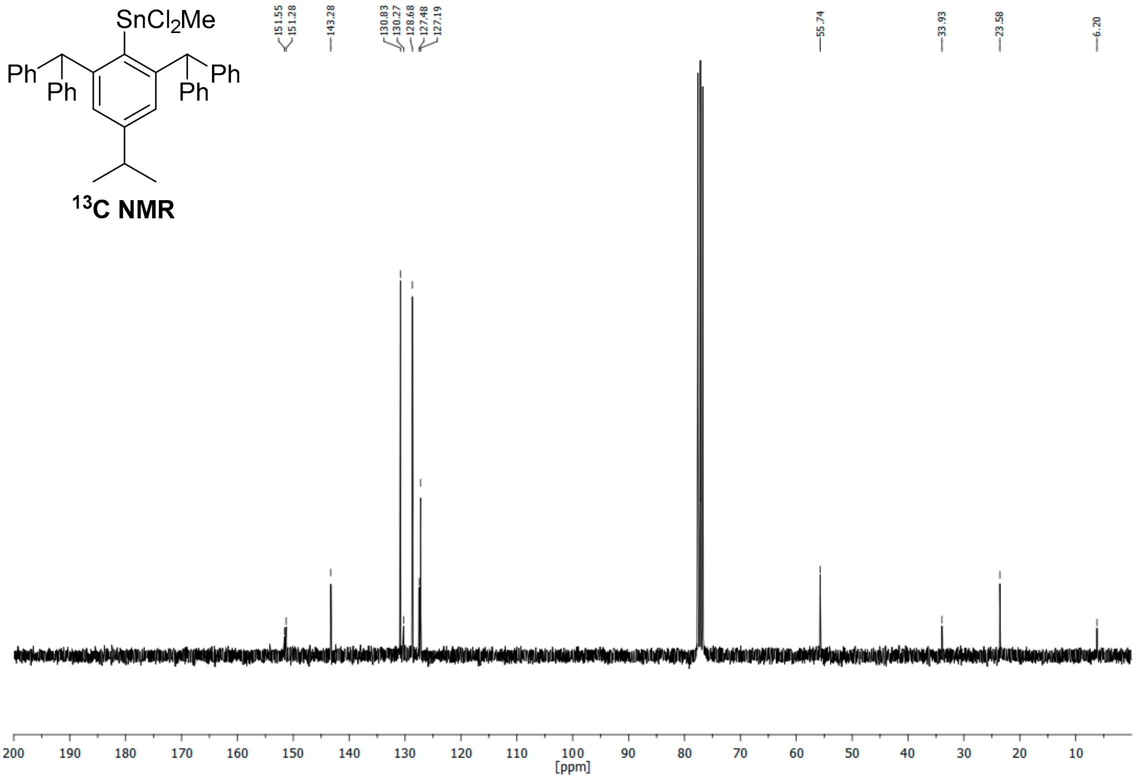

Figure S 24 ^{13}C NMR of ^{iPr}Ar*$SnCl_2Me$ (**8**).

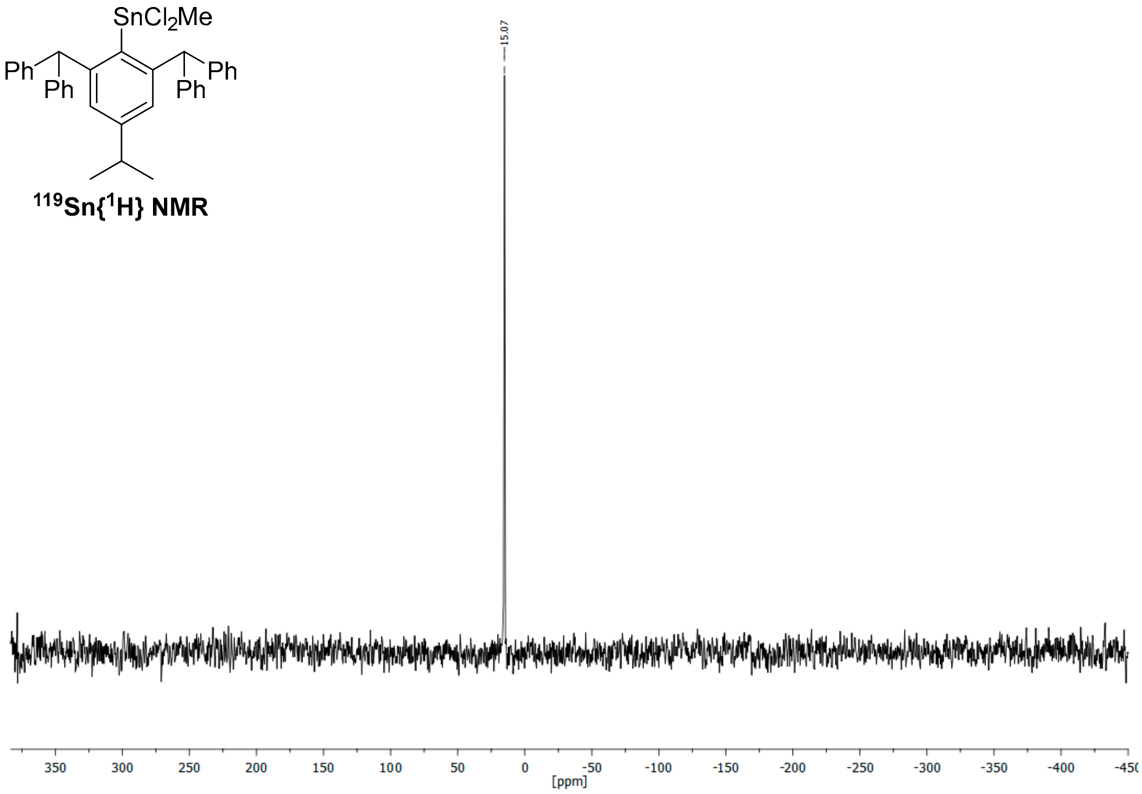

Figure S 25 ^{119}Sn NMR of ^{iPr}Ar*$SnCl_2Me$ (**8**).

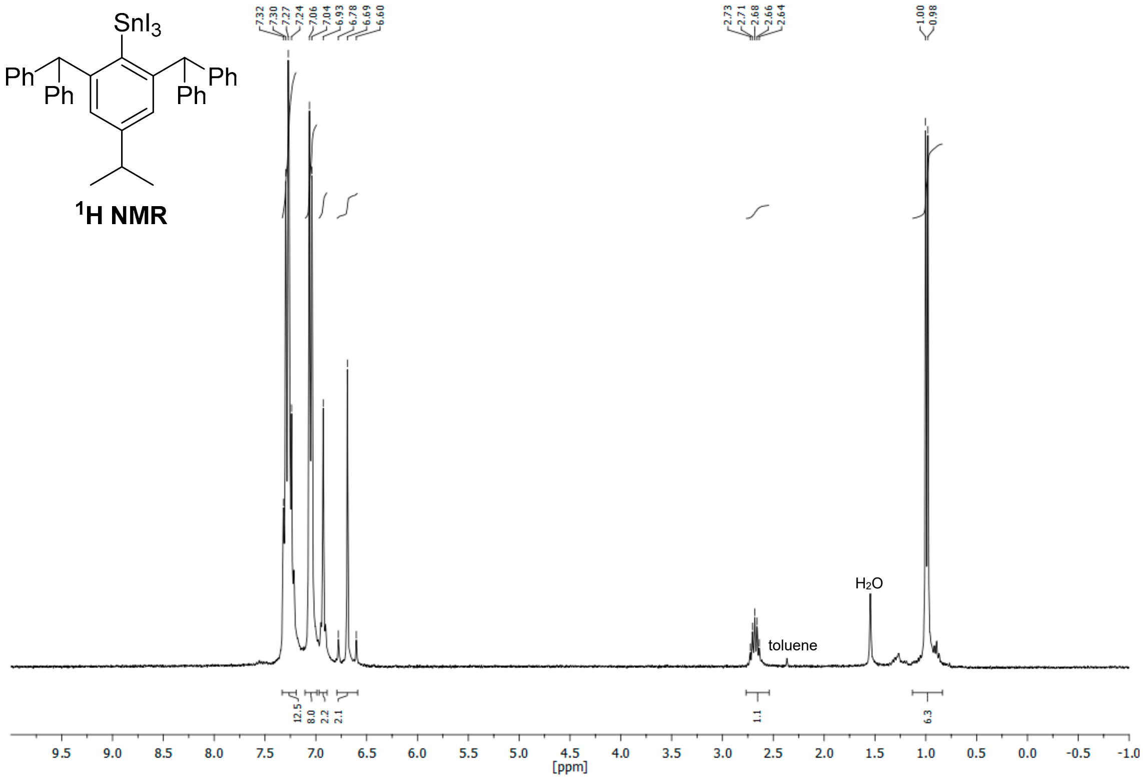

Figure S 26 ^{1}H NMR of ^{iPr}Ar*SnI$_3$ (**9**).

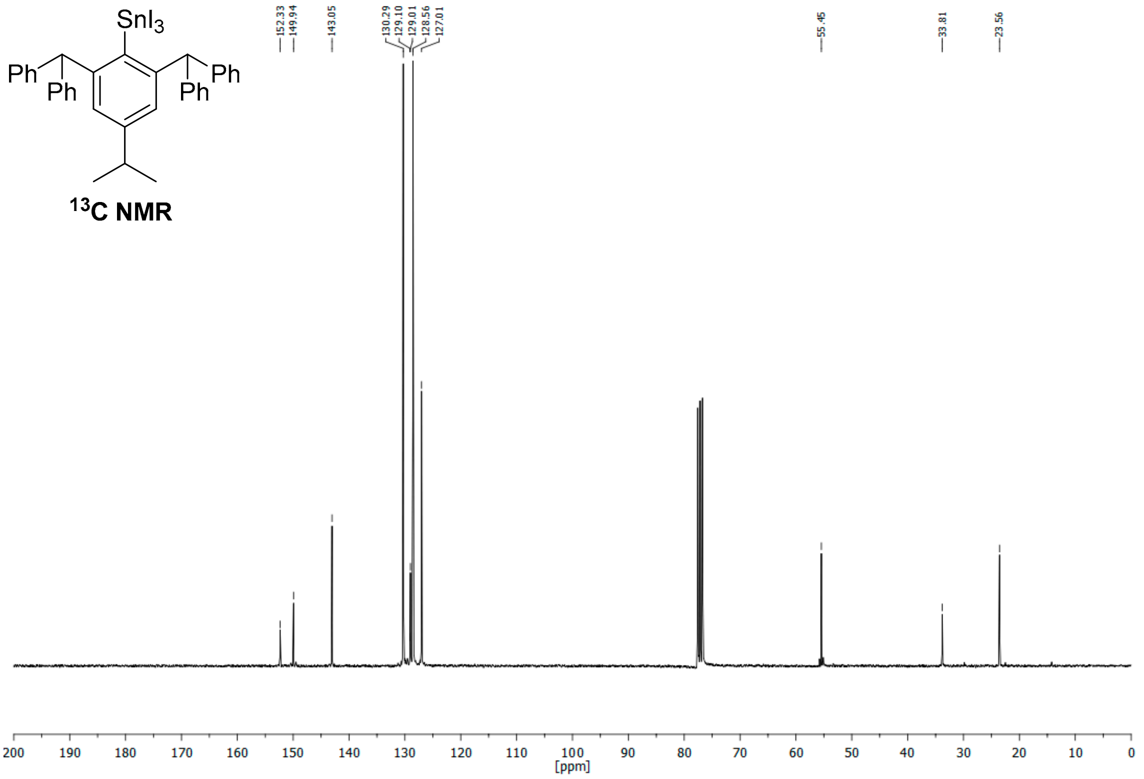

Figure S 27 ^{13}C NMR of ^{iPr}Ar*SnI$_3$ (**9**).

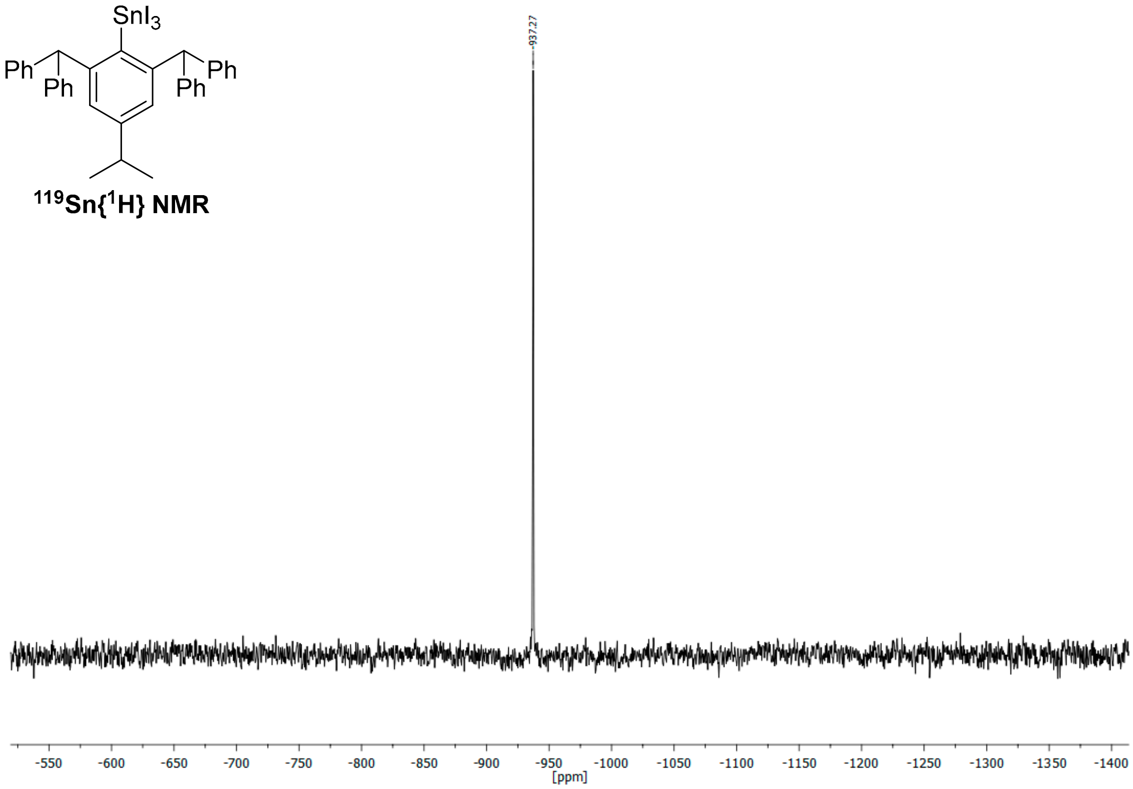

Figure S 28 ^{119}Sn NMR of ^{iPr}Ar*SnI$_3$ (**9**).

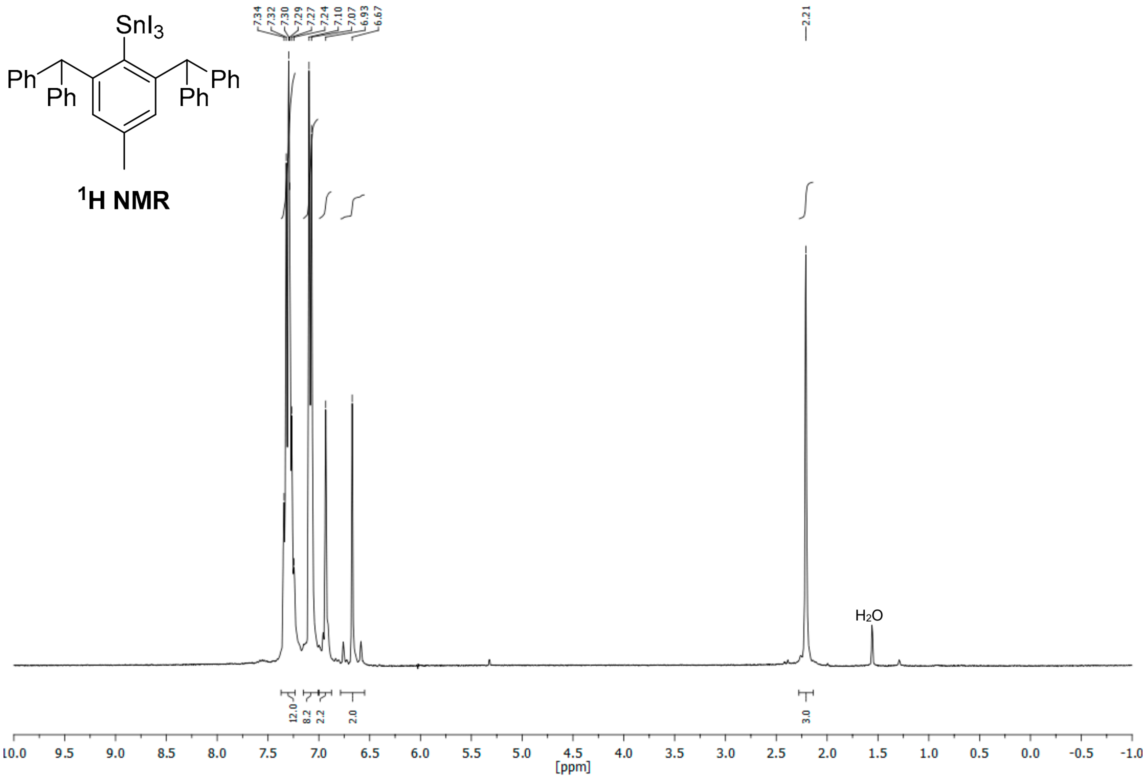

Figure S 29 ^{1}H NMR of ^{Me}Ar*SnI$_3$ (**10**).

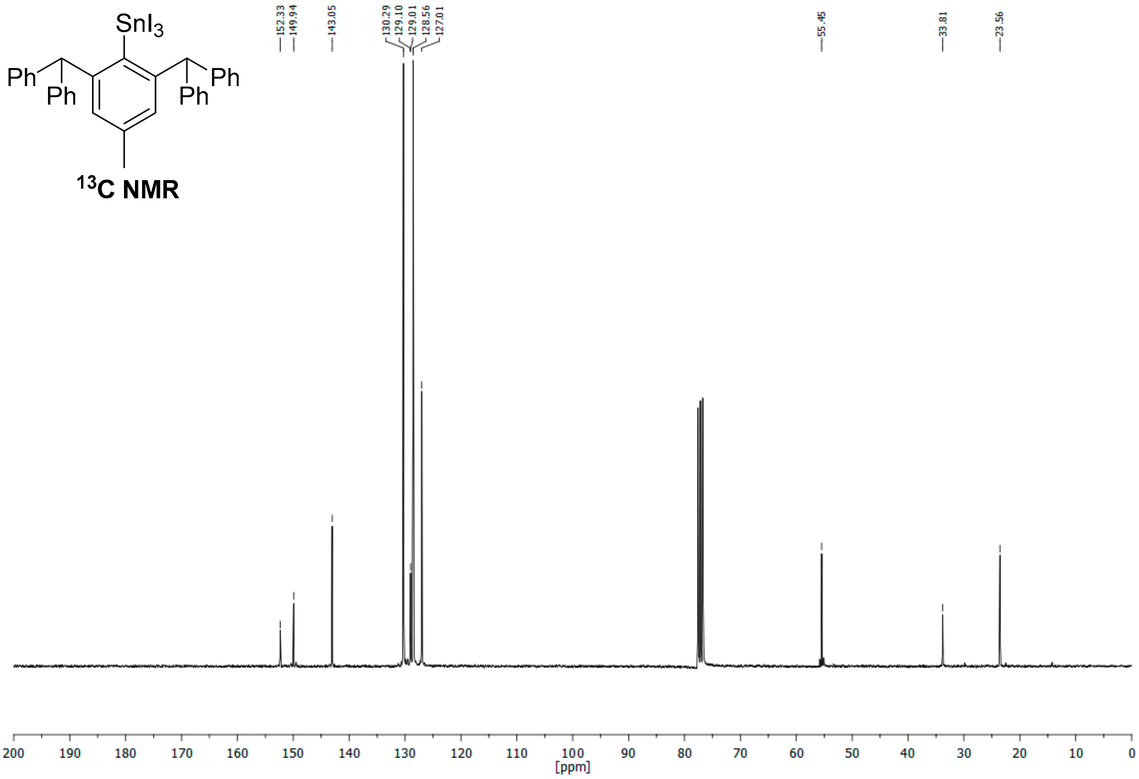

Figure S 30 ^{13}C NMR of ^{Me}Ar*SnI_3 (**10**).

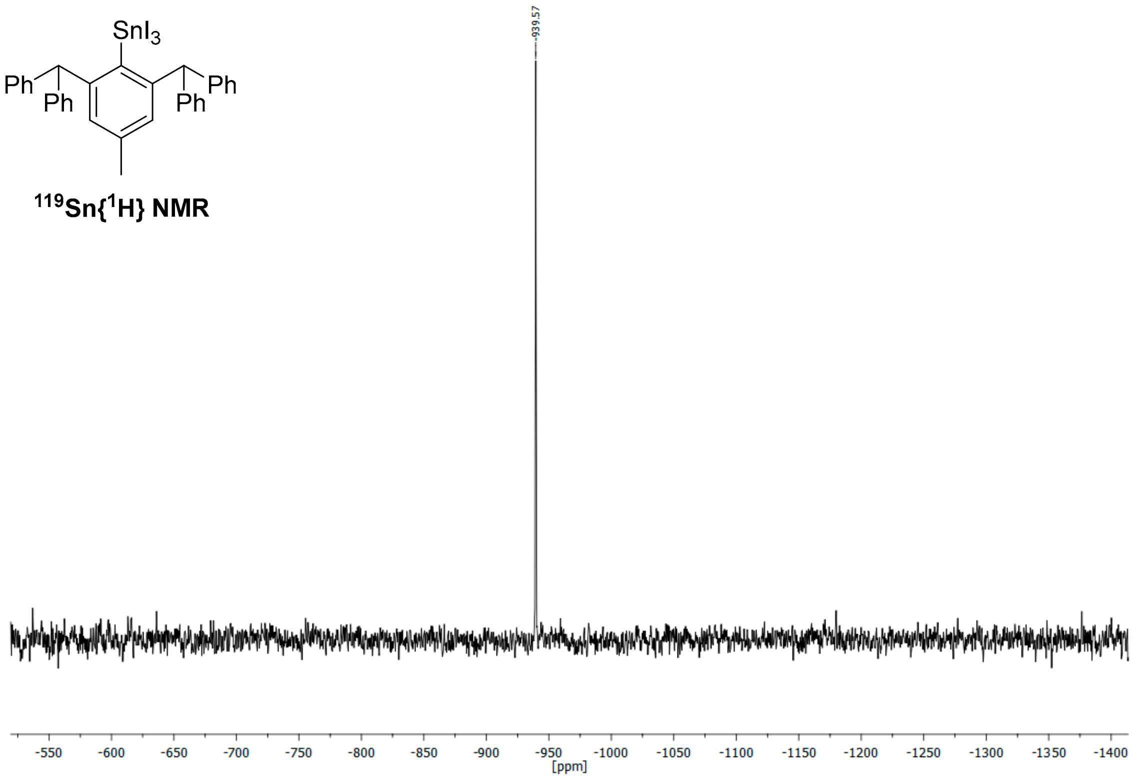

Figure S 31 ^{119}Sn NMR of ^{Me}Ar*SnI_3 (**10**).

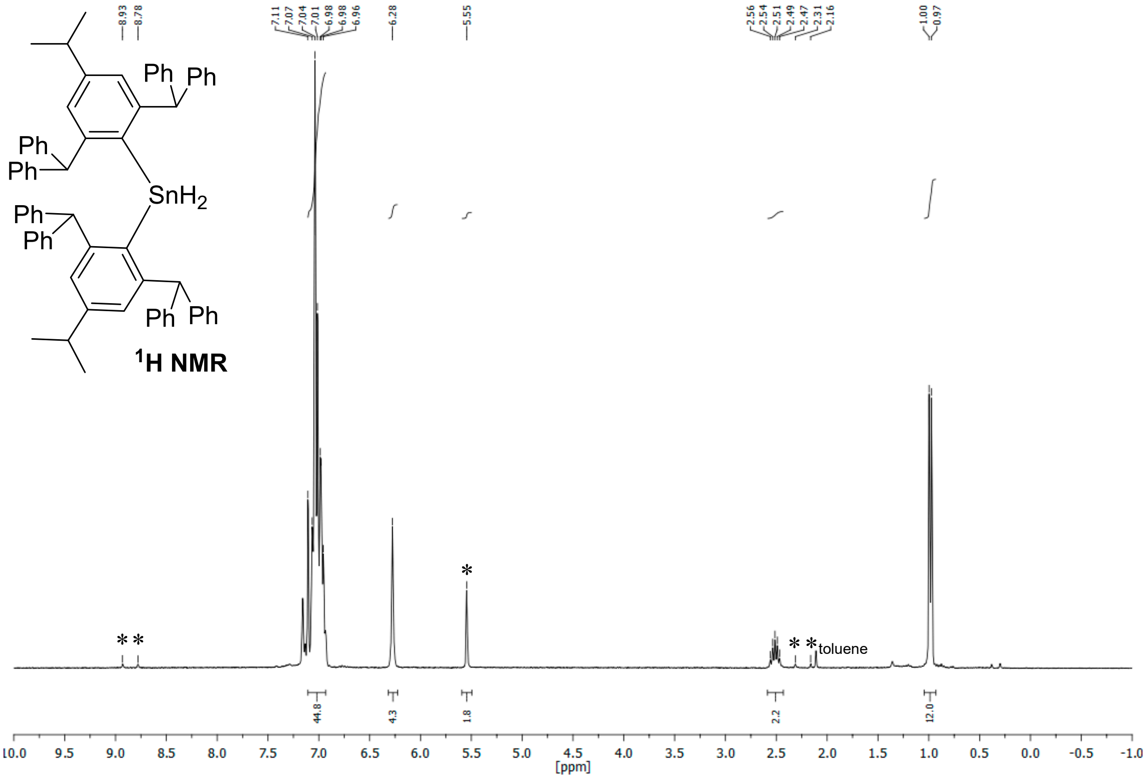

Figure S 32 ^{1}H NMR of ^{iPr}Ar*$_2$SnH$_2$ (**11**). The * indicates SnH and coupling satellites $^1J(^1H, ^{117/119}Sn)$ = 1942/2033 Hz.

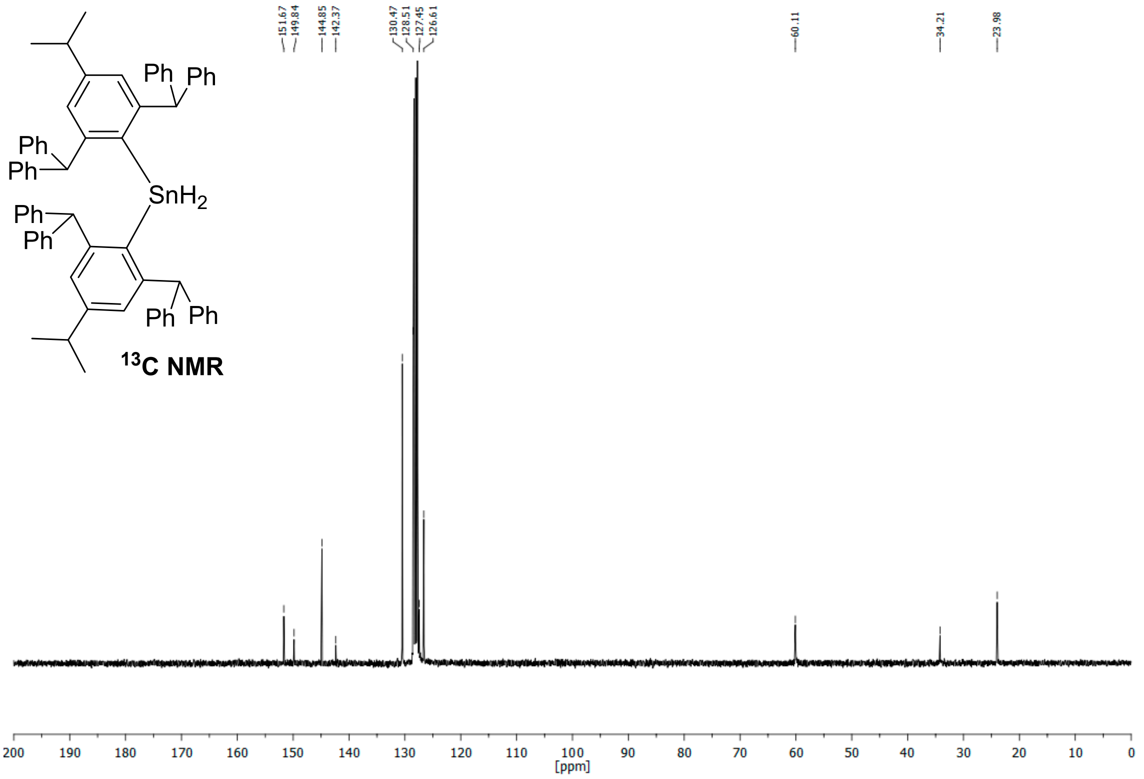

Figure S 33 ^{13}C NMR of ^{iPr}Ar*$_2$SnH$_2$ (**11**).

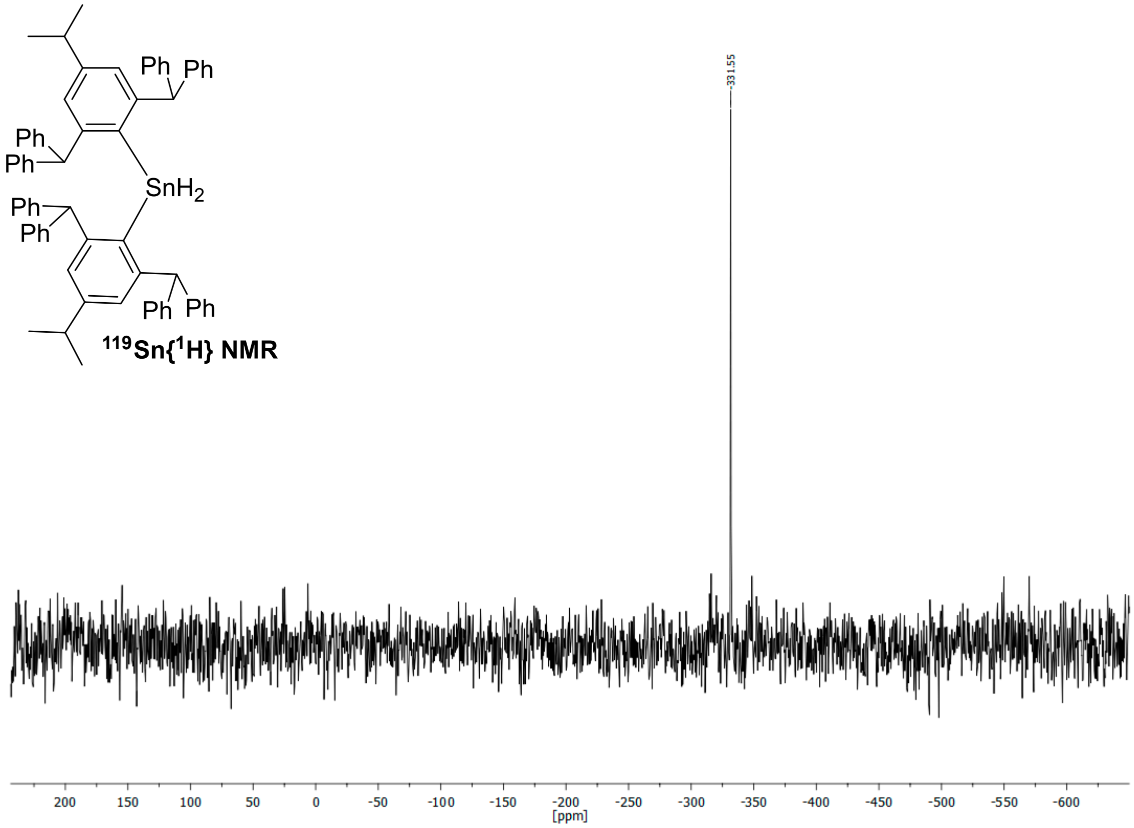

Figure S 34 $^{119}Sn\{^{1}H\}$ NMR of $^{iPr}Ar^{*}{}_{2}SnH_{2}$ (**11**).

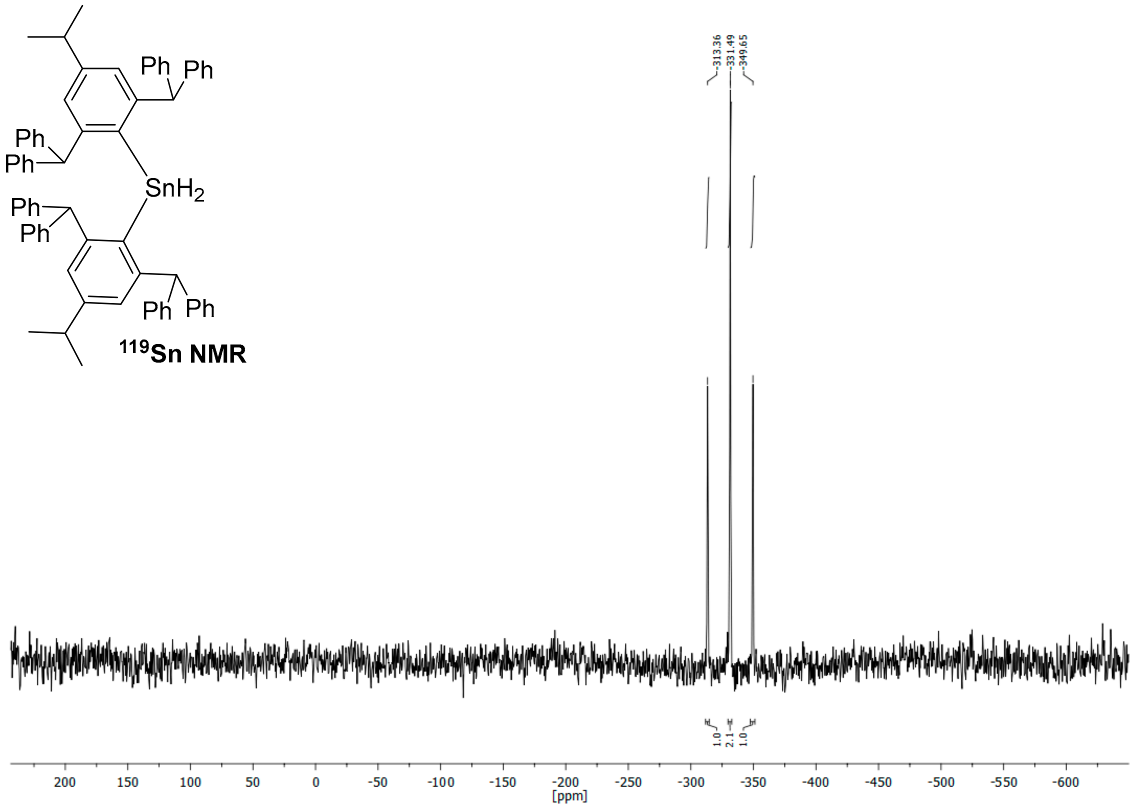

Figure S 35 ^{119}Sn NMR of $^{iPr}Ar^{*}{}_{2}SnH_{2}$ (**11**). $^{1}J(^{119}Sn, ^{1}H)$ = 2033 Hz.

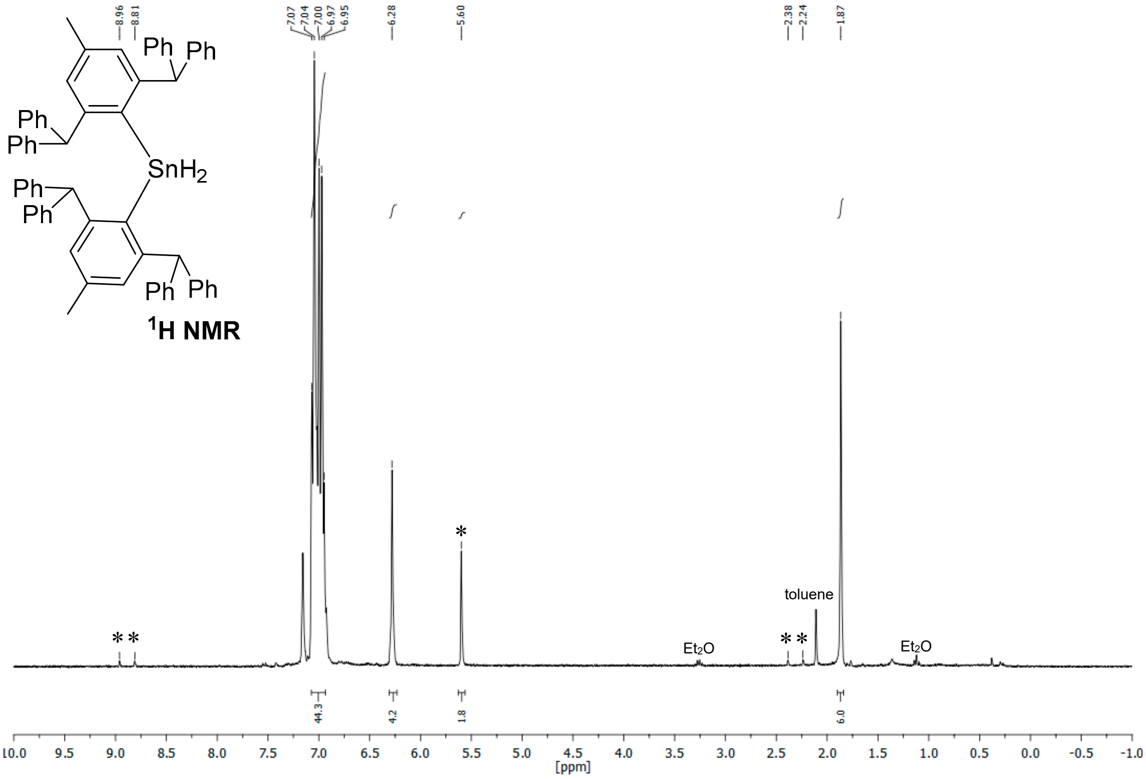

Figure S 36 ^{1}H NMR of ^{Me}Ar*$_2$SnH$_2$ (**12**). The * indicates SnH and coupling satellites $^{1}J(^{1}H, ^{117/119}Sn)$ = 1930/2019 Hz.

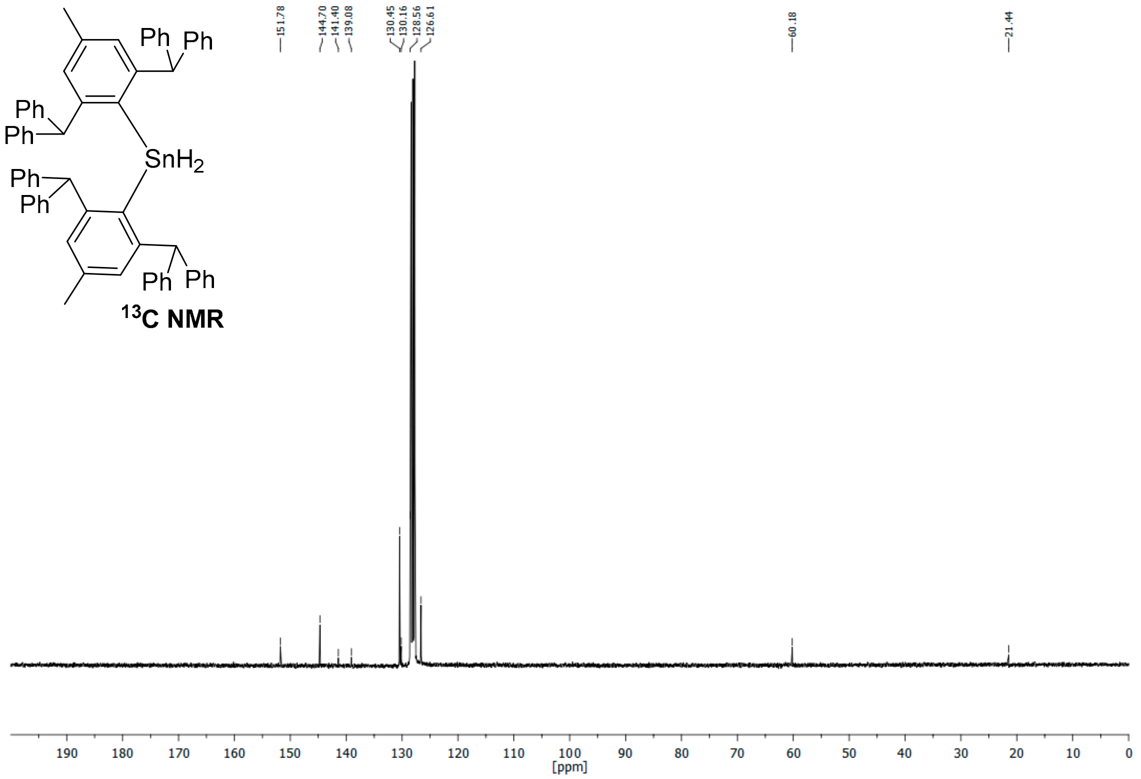

Figure S 37 ^{13}C NMR of ^{Me}Ar*$_2$SnH$_2$ (**12**).

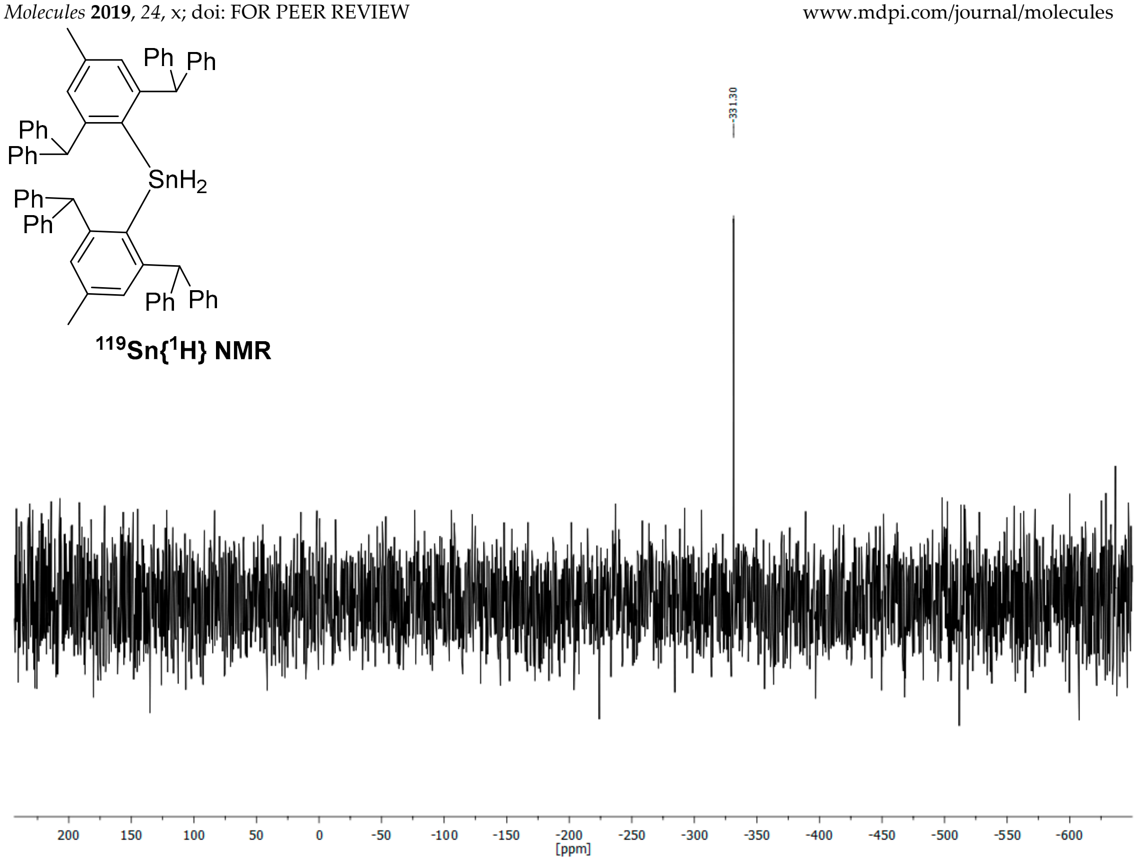

Figure S 38 $^{119}Sn\{^1H\}$ NMR of $^{Me}Ar^*{}_2SnH_2$ (**12**).

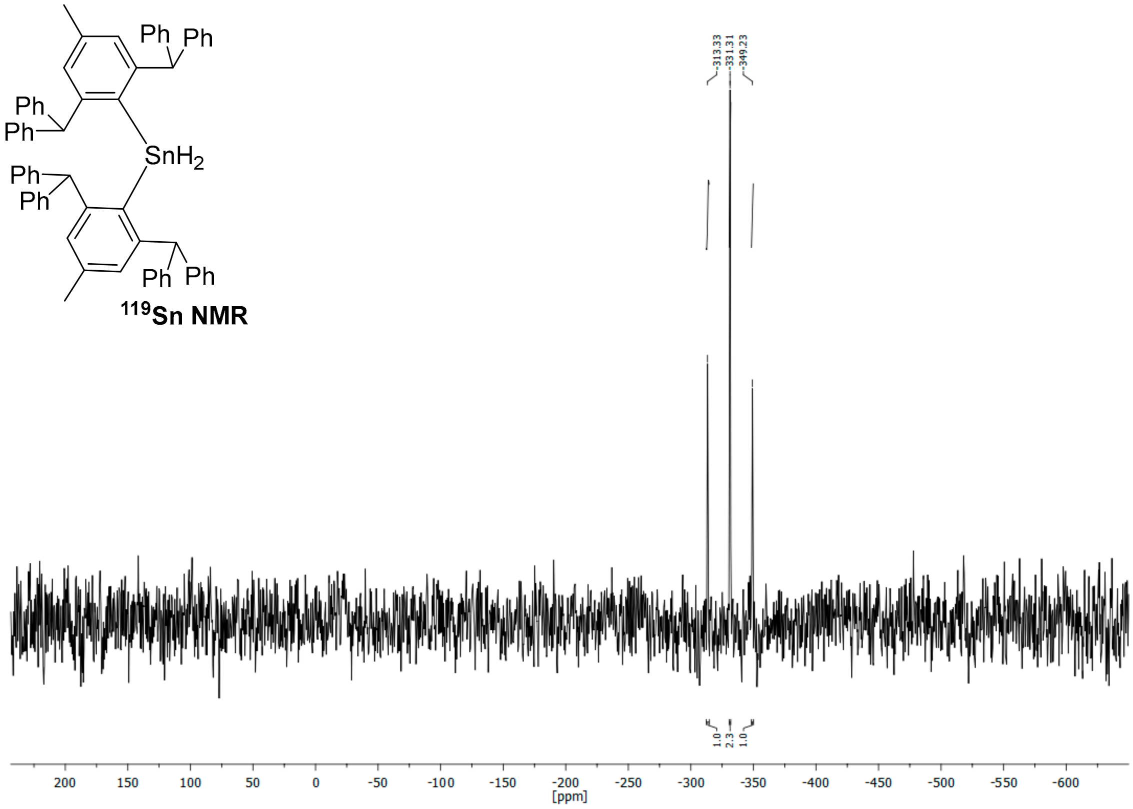

Figure S 39 ^{119}Sn NMR of $^{Me}Ar^*{}_2SnH_2$ (**12**). $^1J(^{119}Sn, ^1H) = 2019$ Hz.

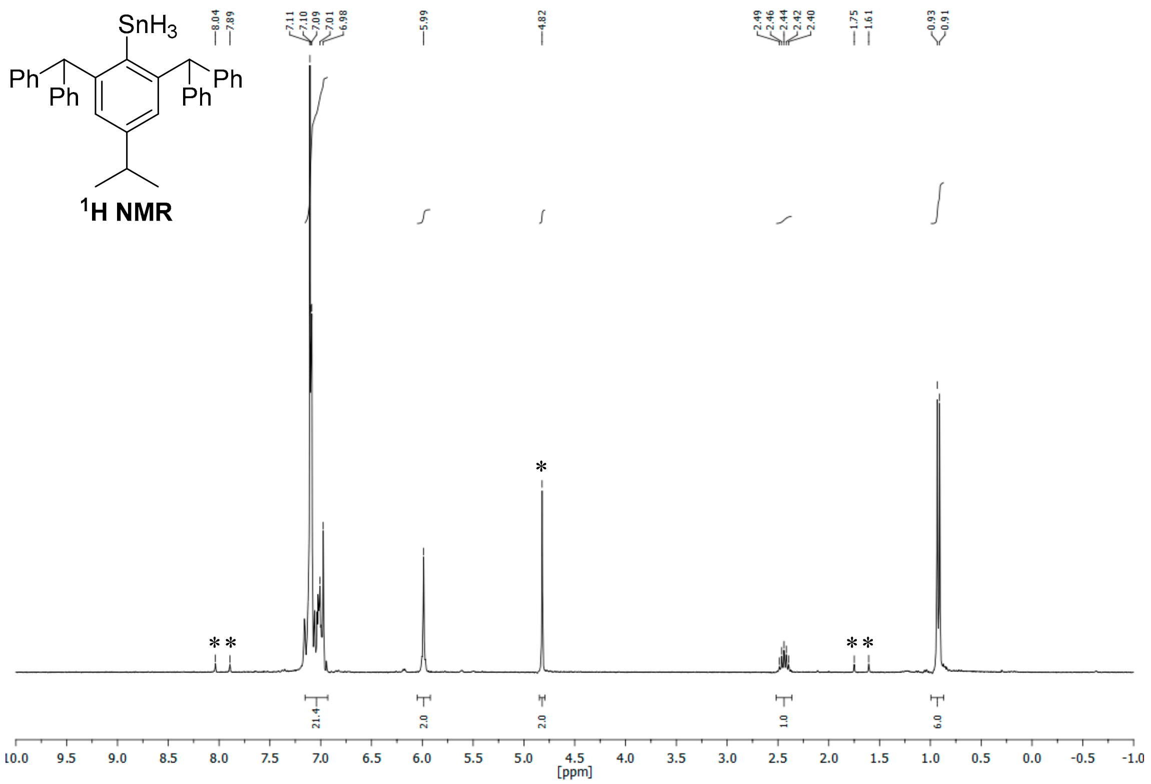

Figure S 40 ^{1}H NMR of ^{iPr}Ar*SnH$_3$ (**13**). The * indicates SnH and coupling satellites $^{1}J(^{1}H, ^{117/119}Sn)$ = 1843 Hz/1930 Hz.

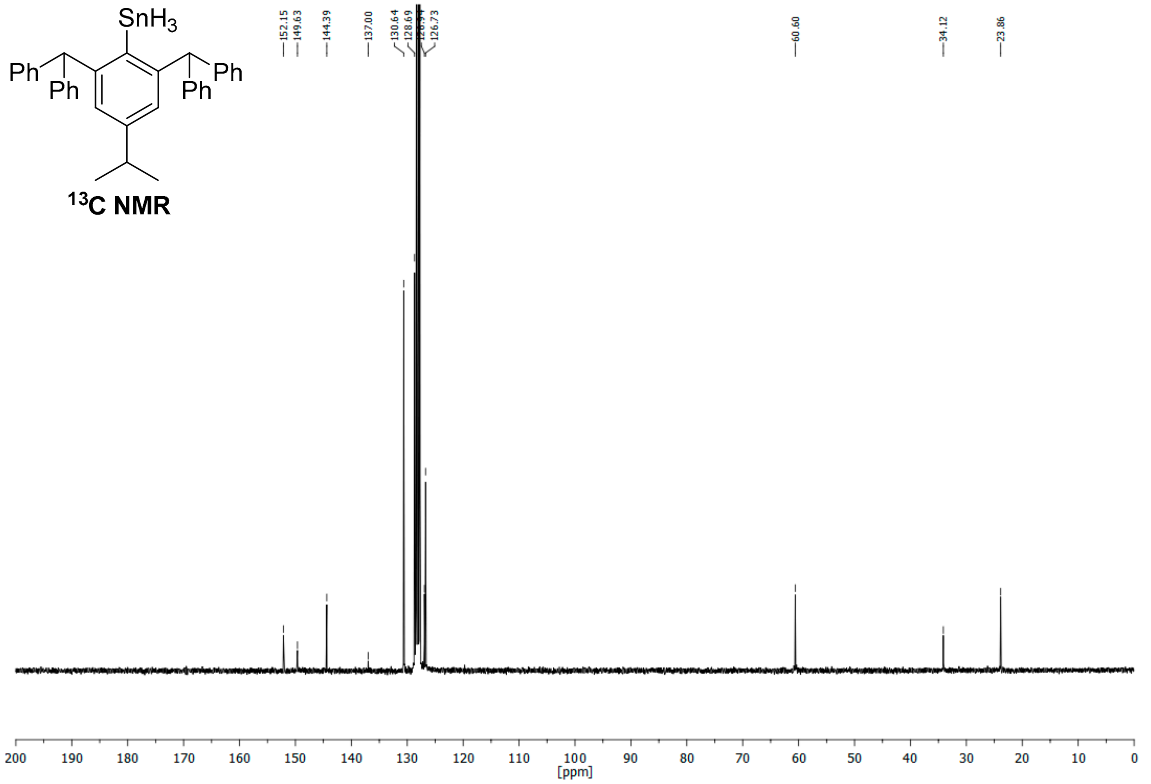

Figure S 41 ^{13}C NMR of ^{iPr}Ar*SnH$_3$ (**13**).

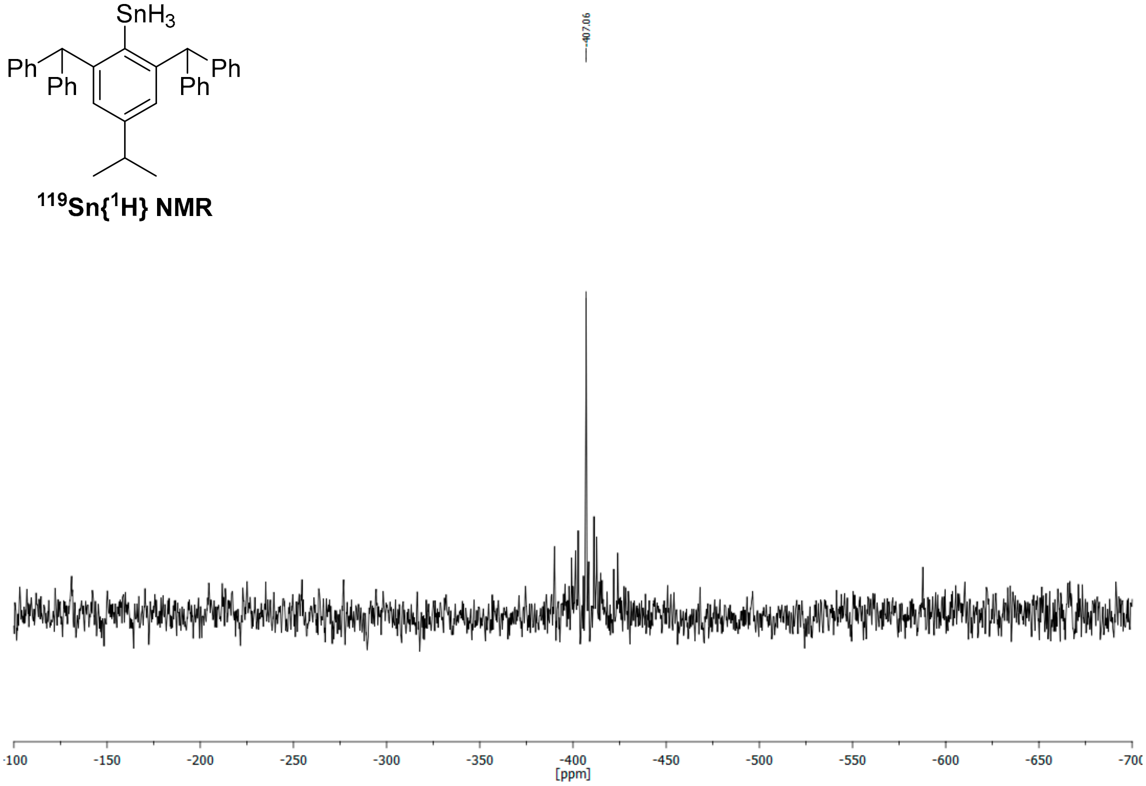

Figure S 42 ^{119}Sn{^{1}H} NMR of ^{iPr}Ar*SnH_3 (**13**).

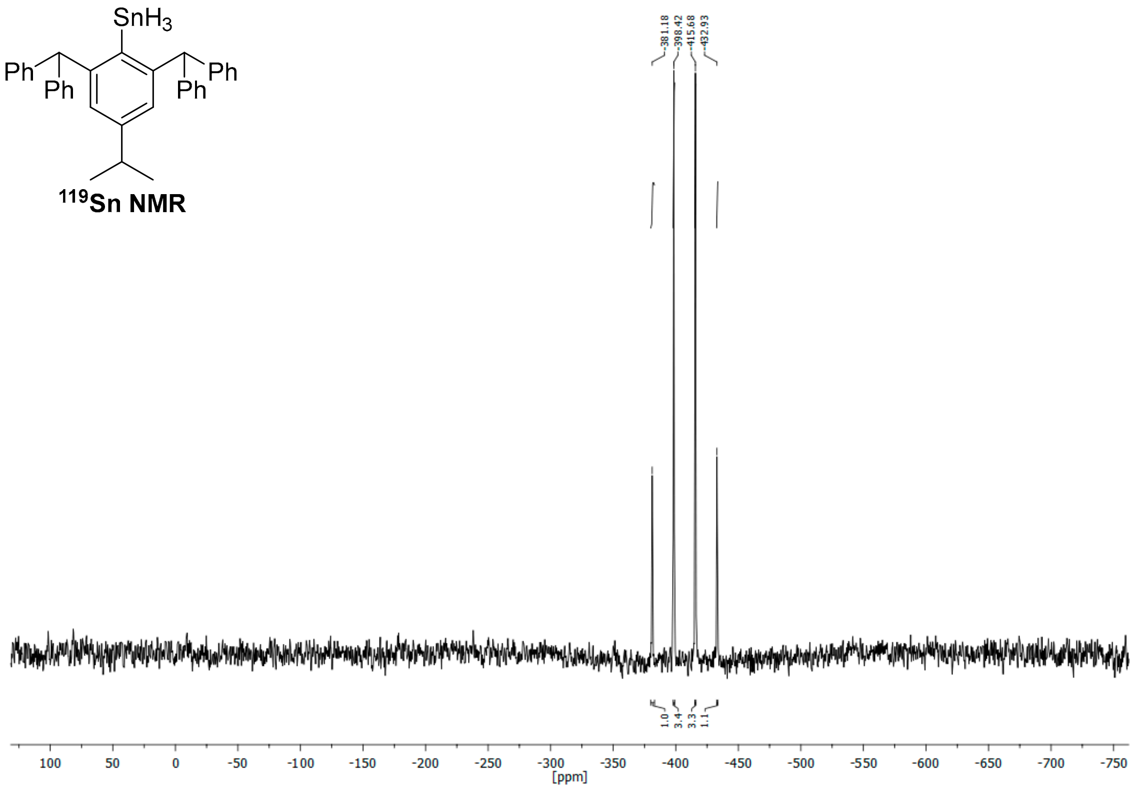

Figure S 43 ^{119}Sn NMR of ^{iPr}Ar*SnH_3 (**13**). $^{1}J(^{119}Sn, ^{1}H)$ = 1930 Hz.

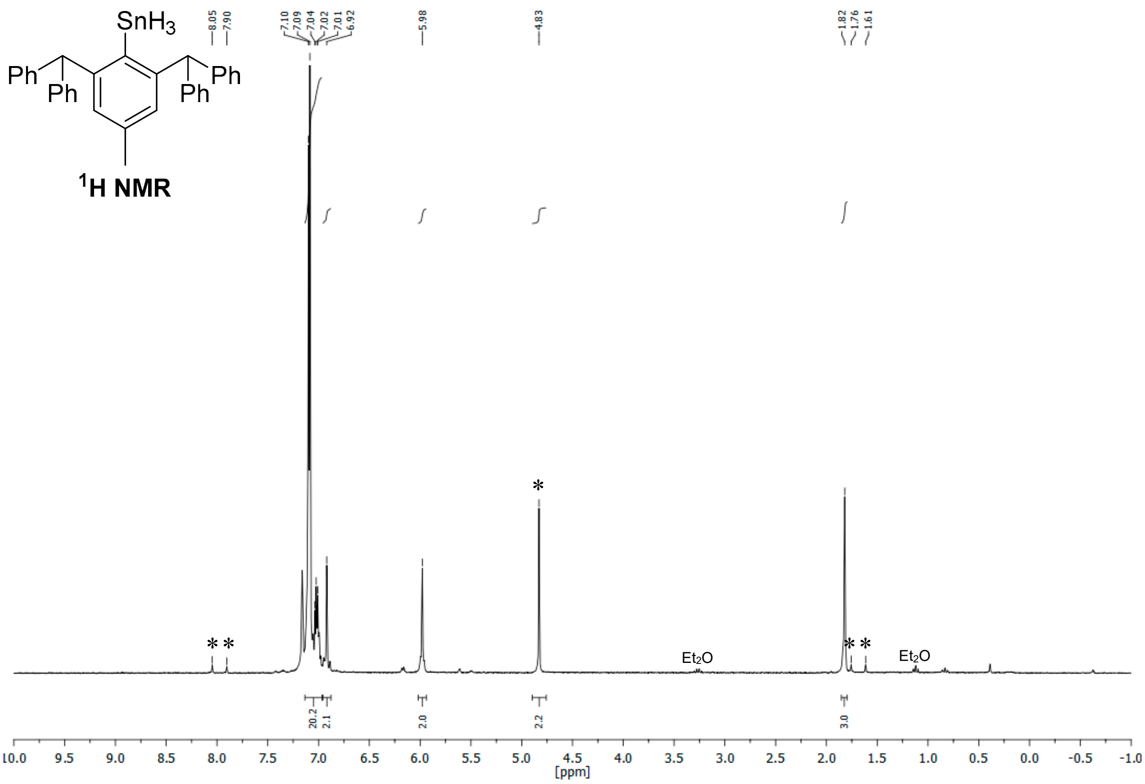

Figure S 44 ^{1}H NMR of ^{Me}Ar*SnH$_3$ (**14**). The * indicates SnH and coupling satellites $^1J(^1H, ^{117/119}Sn)$ = 1845/1931 Hz.

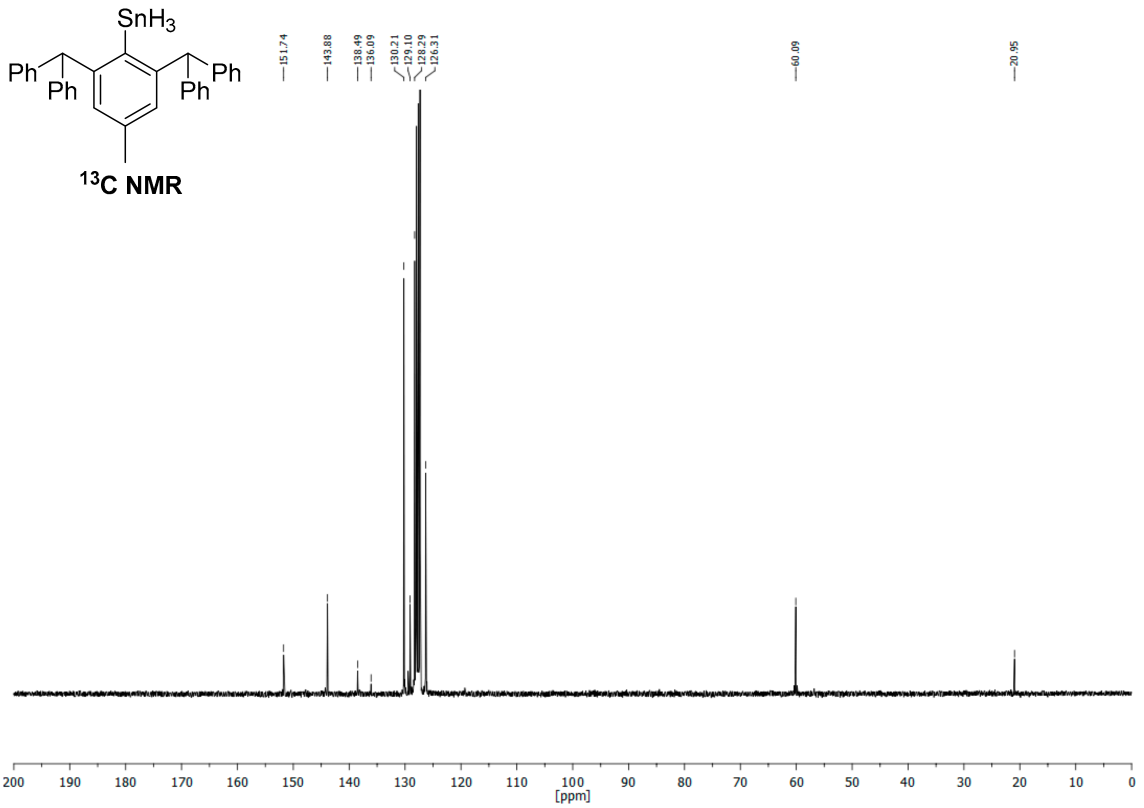

Figure S 45 ^{13}C NMR of ^{Me}Ar*SnH$_3$ (**14**).

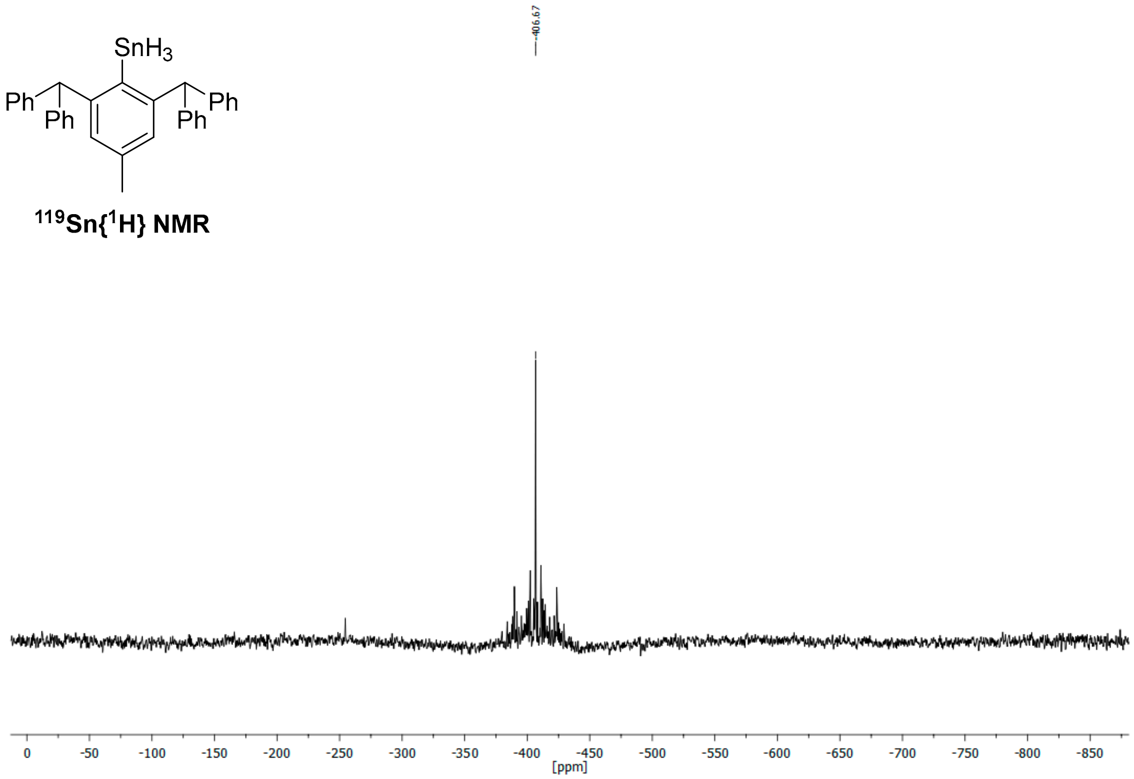

Figure S 46 $^{119}Sn\{^1H\}$ NMR of $^{iPr}Ar^*SnH_3$ (**14**).

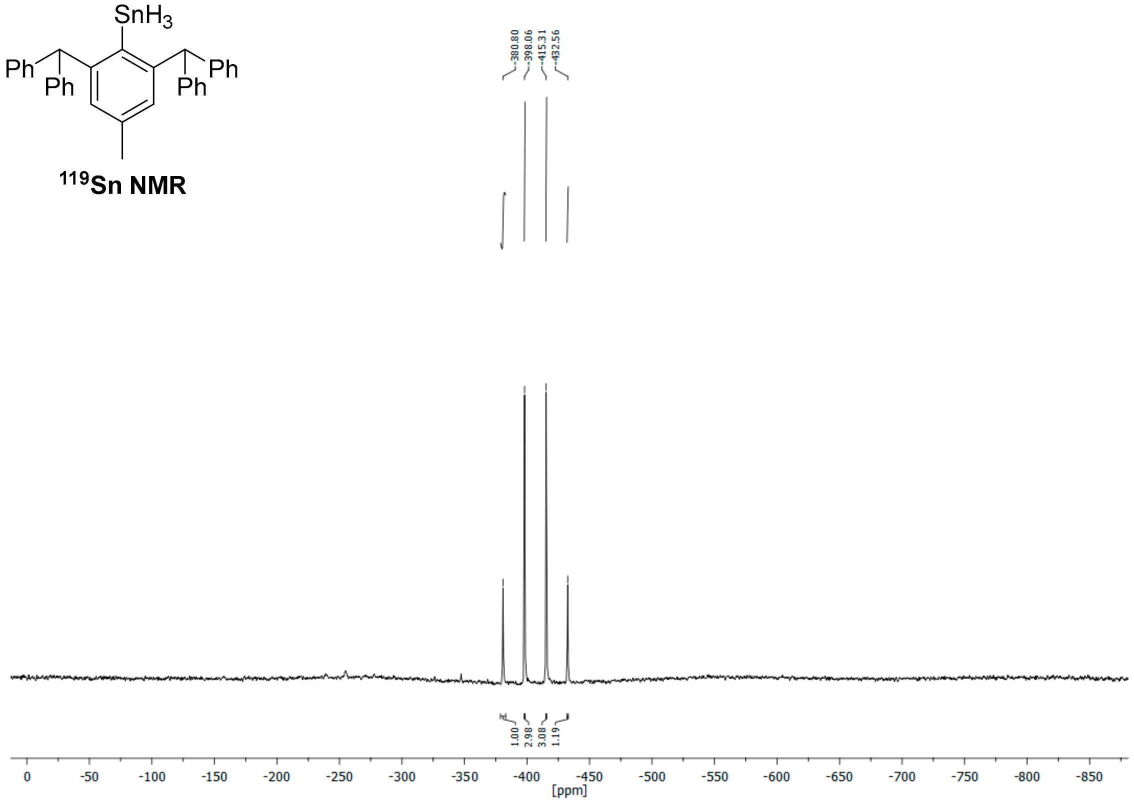

Figure S 47 ^{119}Sn NMR of $^{iPr}Ar^*SnH_3$ (**14**). $^1J(^{119}Sn, ^1H) = 1931$ Hz.

2 ATR-IR spectra

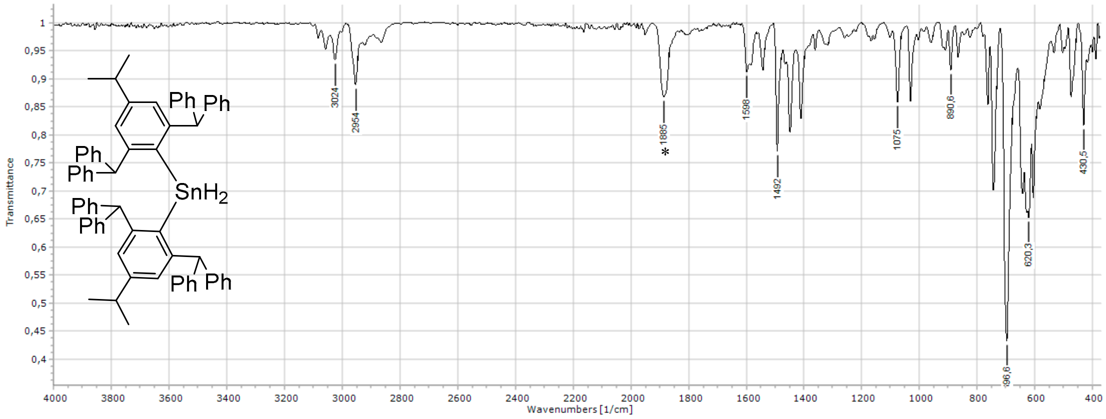

Figure S 48 IR spectra of $^{iPr}Ar^{*}{}_2SnH_2$ (**11**). The * indicates the SnH vibration at 1885 cm^{-1}.

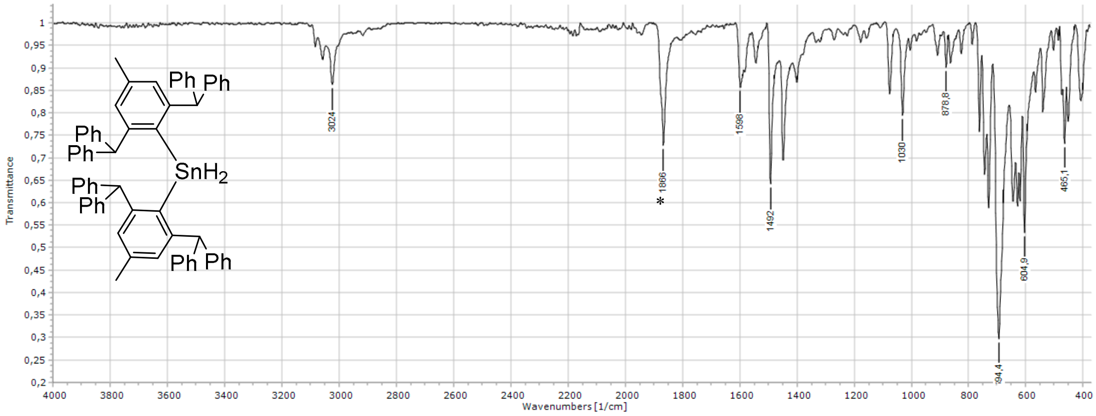

Figure S 49 IR spectra of $^{Me}Ar^{*}{}_2SnH_2$ (**12**). The * indicates the SnH vibration at 1886 cm^{-1}.

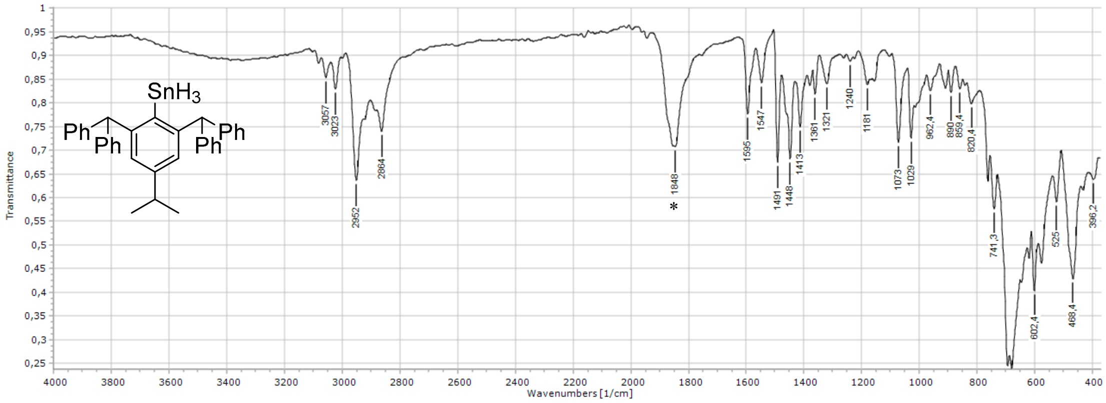

Figure S 50 IR spectra of $^{iPr}Ar^{*}SnH_3$ (**13**). The * indicates the SnH vibration at 1848 cm^{-1}.

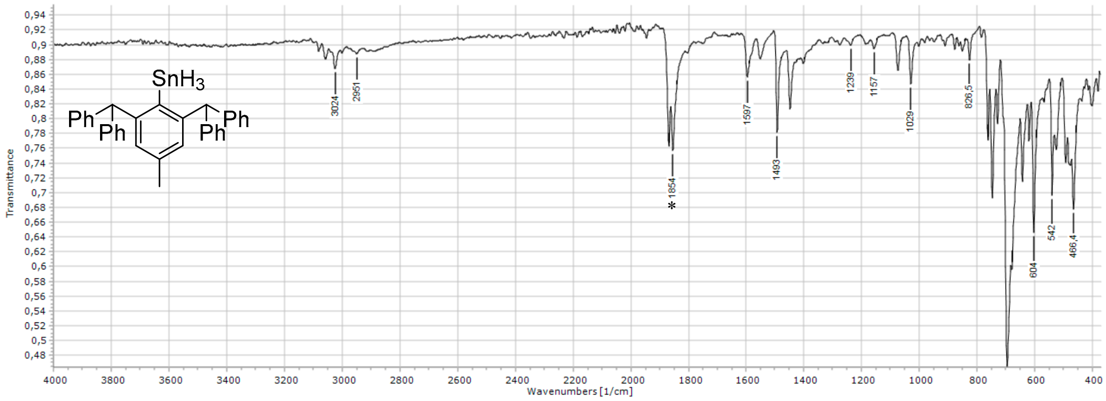

Figure S 51 IR spectra of $^{Me}Ar^{*}SnH_3$ (**14**). The * indicates the SnH vibration at 1854 cm^{-1}.

3 Raman Spectra

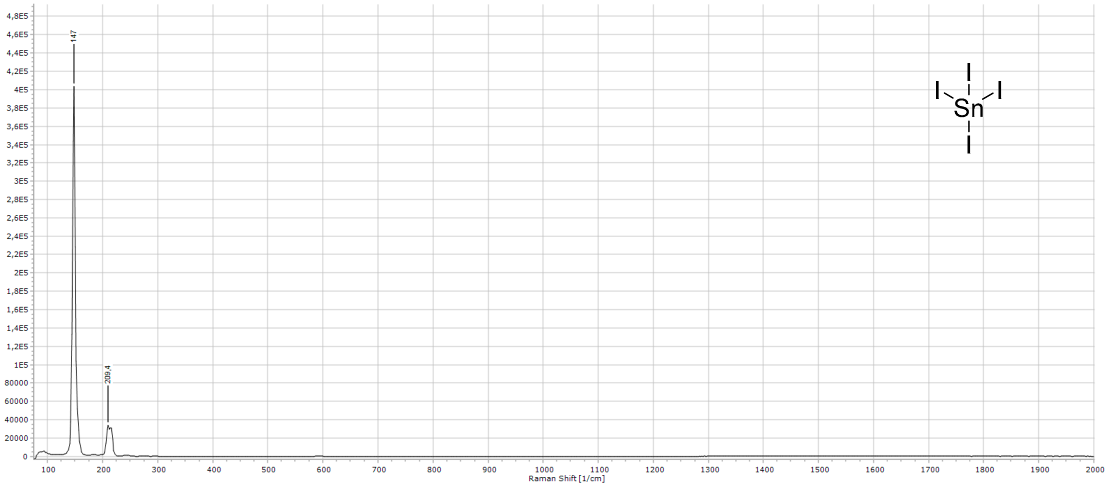

Figure S 52 Raman Spectra of SnI_4.

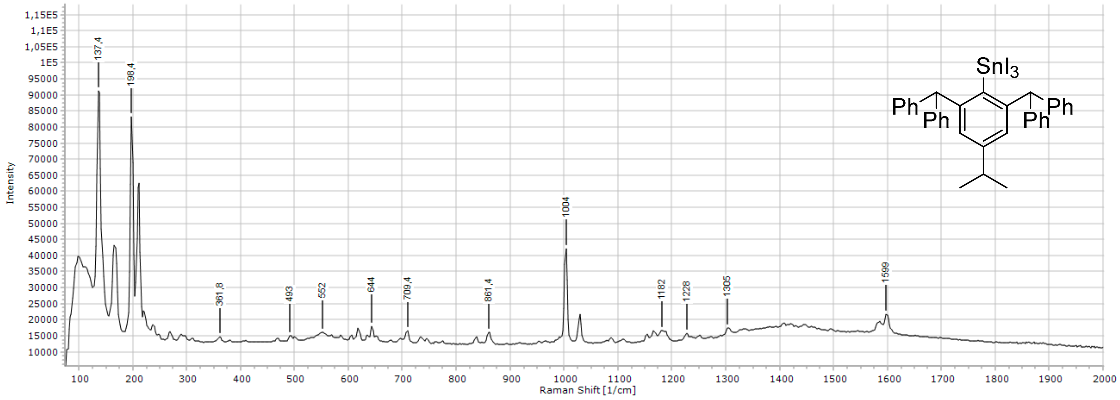

Figure S 53 Raman Spectra of $^{iPr}Ar*SnI_3$ (**9**).

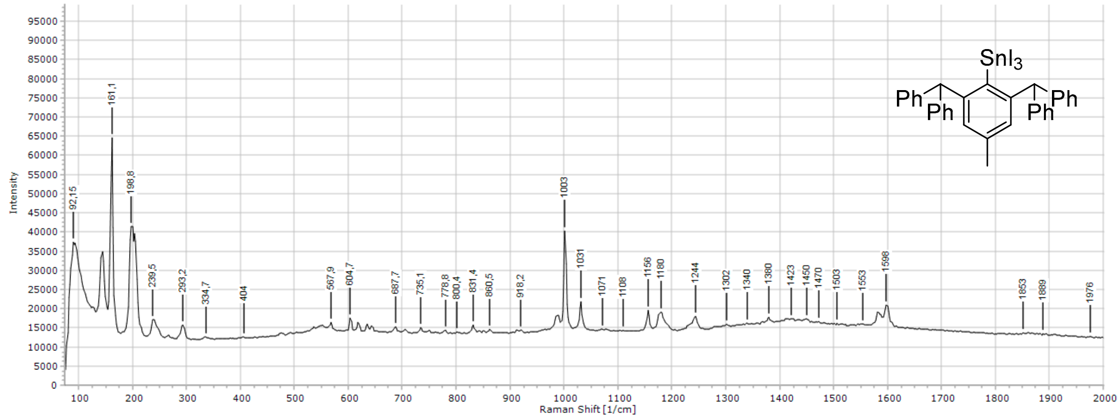

Figure S 54 Raman Spectra of $^{Me}Ar*SnI_3$ (**10**).

4 Crystal Structures and crystallographic tables

^{iPr}Ar*I (**1**)

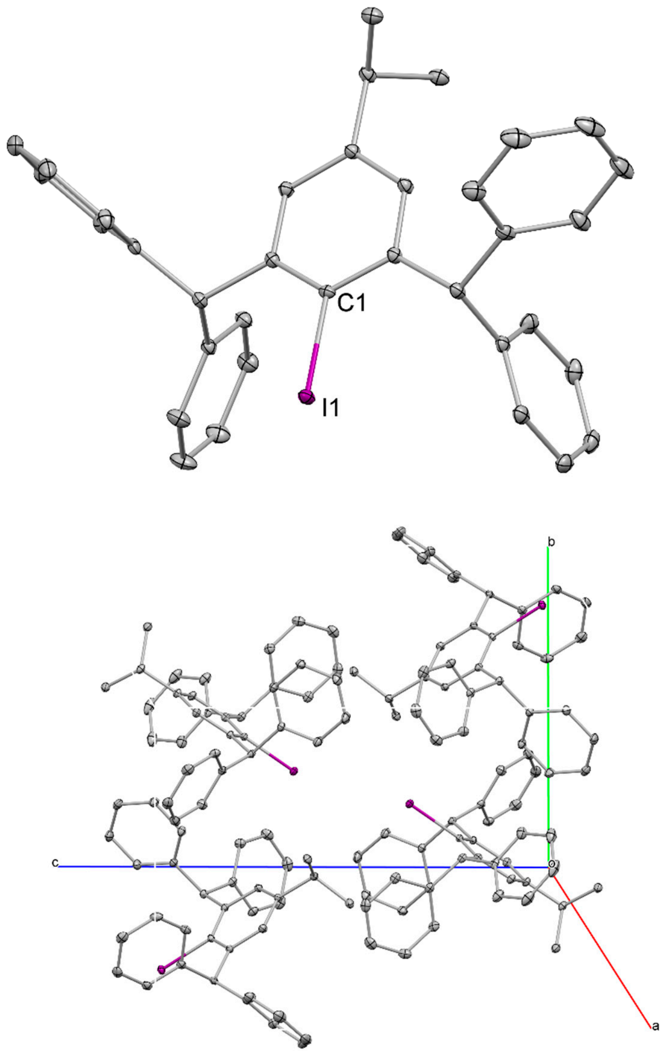

Figure S 55 Crystal structure and packing of ^{iPr}Ar*I (**1**). All non-hydrogen atoms shown as 30% shaded ellipsoids. Hydrogen atoms are omitted for clarity. Selected bond lengths [Å] : C1-I1 2.119(2).

Table S 1 Crystal data and structure refinement ^{iPr}Ar*I (**1**).

CCDC number	1983429
Empirical formula	$C_{35}H_{31}I$
Formula weight	578.50
Temperature/K	100.0
Crystal system	monoclinic
Space group	$P2_1/c$
a [Å]	13.7510(6)
b [Å]	12.1590(6)
c [Å]	17.0679(7)
a [°]	90
β [°]	105.283(2)
γ [°]	90
Volume [Å]	2752.8(2)
Z	4
ρ_{calc} [g/cm^3]	1.396
μ [mm^{-1}]	1.185
F(000)	1176.0
Crystal size [mm^3]	0.27 × 0.20 × 0.16
Radiation	MoKα (λ = 0.71073)
2Θ range for data collection [°]	4.164 to 59.996
Index ranges	-19 ≤ h ≤ 19, -17 ≤ k ≤ 16, -24 ≤ l ≤ 24
Reflections collected	149302
Independent reflections	8034 [R_{int} = 0.0620, R_{sigma} = 0.0241]
Data/restraints/parameters	8034/0/327
Goodness-of-fit on F^2	1.056
Final R indexes [I>=2σ (I)]	R_1 = 0.0240, wR_2 = 0.0570
Final R indexes [all data]	R_1 = 0.0316, wR_2 = 0.0624
Largest diff. peak/hole [e Å^{-3}]	0.86/-0.67

^{iPr}Ar*Li (**3**) x Et_2O

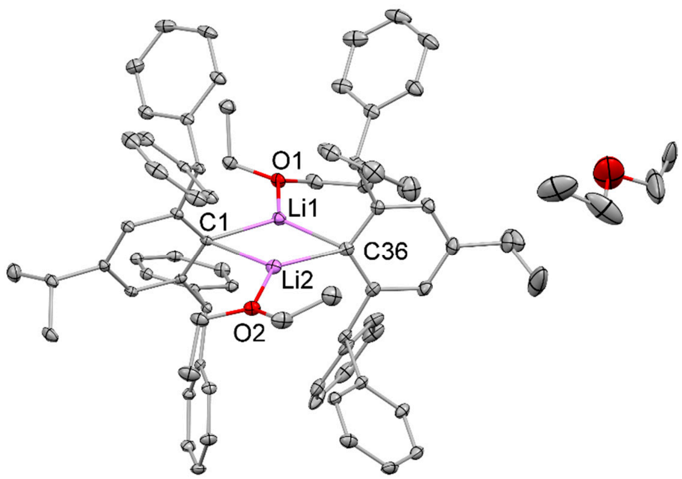

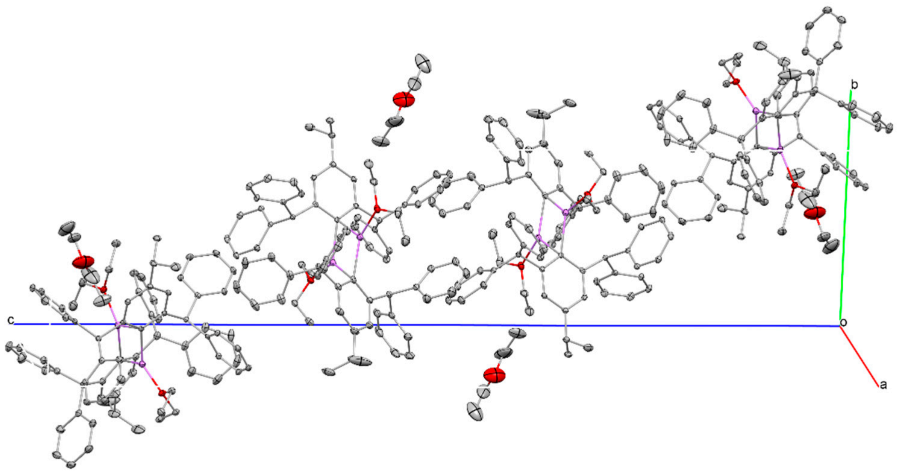

Figure S 56 Crystal structure and packing of ^{iPr}Ar*Li (**3**) x Et_2O. All non-hydrogen atoms shown as 30% shaded ellipsoids. Hydrogen atoms are omitted for clarity. Selected bond lengths [Å] and angels [°]: C1-Li1 2.14(5), C1-Li2 2.269(6), C36-Li1 2.274(6), C36-Li2 2.235(6), O1→Li1 1.921(5), O2→Li2 1.972(5), C1-Li1-C36 114.5(2), C1-Li2-C36 113.9(2), Li1-C2-Li2 65.9(2), Li1-C36-Li2 65.5(2).

Table S 2 Crystal data and structure refinement ^{iPr}Ar*Li (**3**) x Et_2O.

CCDC number	1983430
Empirical formula	$C_{40}H_{43.5}LiO_{1.25}$
Formula weight	551.19
Temperature/K	100.01
Crystal system	monoclinic
Space group	$P2_1/c$
a [Å]	12.5284(3)
b [Å]	12.6079(3)
c [Å]	43.3791(12)
a [°]	90
β [°]	90.2040(10)
γ [°]	90
Volume [Å]	6852.0(3)
Z	8
ρ_{calc} [g/cm^3]	1.069
μ [mm^{-1}]	0.062
F(000)	2372.0
Crystal size [mm^3]	0.24 × 0.15 × 0.11
Radiation	MoKα (λ = 0.71073)
2Θ range for data collection [°]	3.736 to 51.998
Index ranges	$-15 \leq h \leq 11$, $-15 \leq k \leq 15$, $-53 \leq l \leq 53$
Reflections collected	76053
Independent reflections	13351 [R_{int} = 0.0623, R_{sigma} = 0.0602]
Data/restraints/parameters	13351/0/794
Goodness-of-fit on F^2	1.075
Final R indexes [I>=2σ (I)]	R_1 = 0.0775, wR_2 = 0.1999
Final R indexes [all data]	R_1 = 0.1133, wR_2 = 0.2235
Largest diff. peak/hole [e Å^{-3}]	1.04/-0.31

^{Me}Ar*Li (**4**) x 2 Et_2O

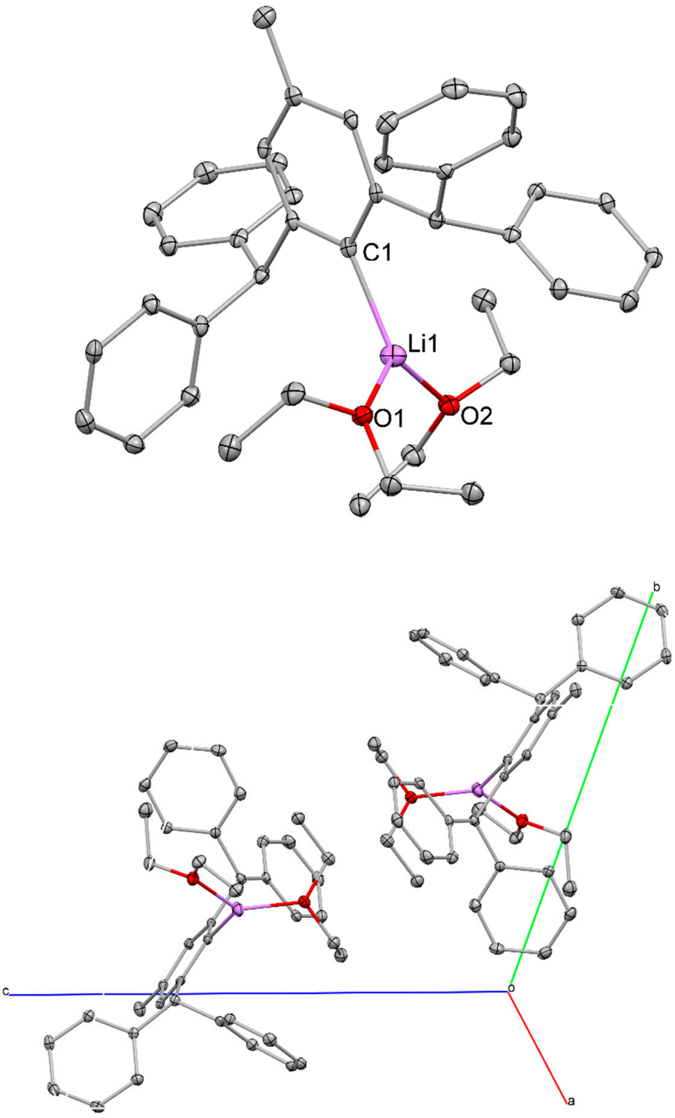

Figure S 57 Crystal structure and packing of ^{Me}Ar*Li x 2 Et_2O. All non-hydrogen atoms shown as 30% shaded ellipsoids. Hydrogen atoms are omitted for clarity. Selected bond lengths [Å] and angels [°]: C1-Li1 2.107(8), O1→Li1 1.960(6), O2→Li1 1.954 (9), O1-Li1-O2 118.2(4).

Table S 3 Crystal data and structure refinement ^{Me}Ar*Li (**4**) x 2 Et_2O.

CCDC number	1983431
Empirical formula	$C_{41}H_{47}LiO_2$
Formula weight	578.72
Temperature/K	100.0
Crystal system	triclinic
Space group	P-1
a [Å]	11.232(15)
b [Å]	12.182(17)
c [Å]	14.561(18)
a [°]	110.38(4)
β [°]	105.54(3)
γ [°]	100.73(3)
Volume [Å]	1711(4)
Z	2
ρ_{calc} [g/cm^3]	1.123
μ [mm^{-1}]	0.066
F(000)	624.0
Crystal size [mm^3]	0.26 × 0.18 × 0.16
Radiation	MoKα (λ = 0.71073)
2Θ range for data collection [°]	3.2 to 51.988
Index ranges	$-13 \leq h \leq 13$, $-15 \leq k \leq 15$, $-17 \leq l \leq 17$
Reflections collected	32609
Independent reflections	6696 [R_{int} = 0.1119, R_{sigma} = 0.1194]
Data/restraints/parameters	6696/0/402
Goodness-of-fit on F^2	1.015
Final R indexes [I>=2σ (I)]	R_1 = 0.0855, wR_2 = 0.2379
Final R indexes [all data]	R_1 = 0.1466, wR_2 = 0.2872
Largest diff. peak/hole [e Å^{-3}]	0.63/-0.37

^{iPr}Ar*$_2$SnCl$_2$ (**5**)

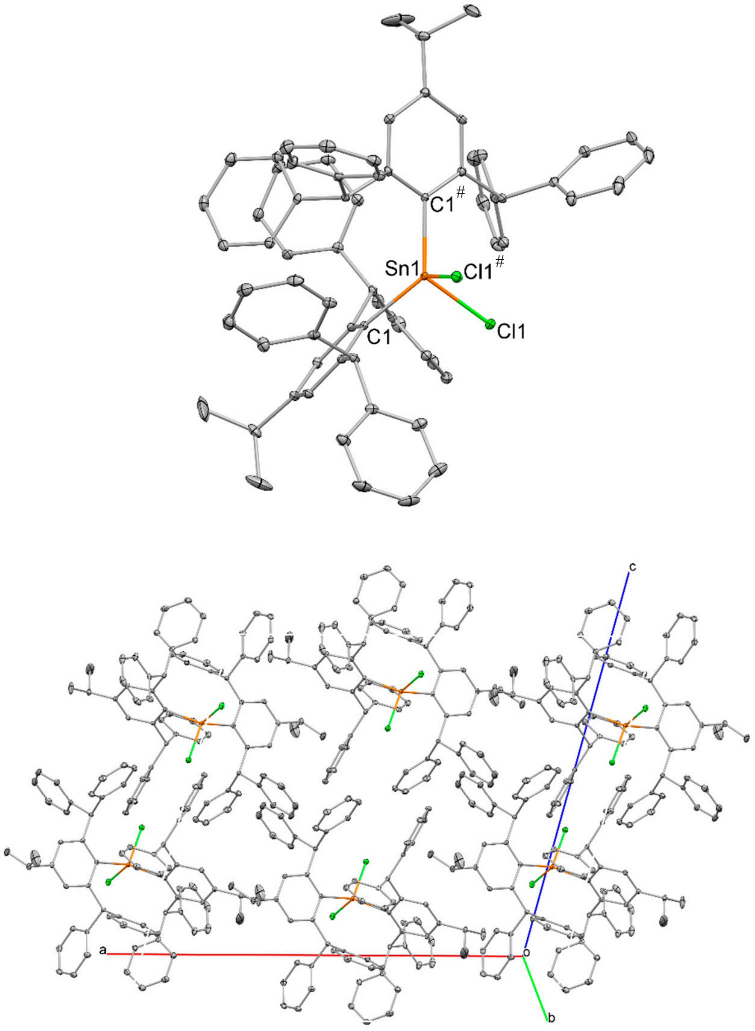

Figure S 58 Crystal structure and packing of ^{iPr}Ar*$_2$SnCl$_2$ (**5**). All non-hydrogen atoms shown as 30% shaded ellipsoids. Hydrogen atoms are omitted for clarity. Selected bond lengths [Å] and angels [°]: Sn1-C1 2.1501(15), Sn1-Cl1 2.3781(5), C1-Sn1-C1# 125.77(7), C1-Sn1-Cl1 118.56(4), Cl1-Sn1-Cl1 94.49(3).

Table S 4 Crystal data and structure refinement $^{iPr}Ar^{*}{}_{2}SnCl_{2}$ (**5**).

CCDC number	1983436
Empirical formula	$C_{35}H_{31}ClSn_{0.5}$
Formula weight	546.39
Temperature/K	99.99
Crystal system	monoclinic
Space group	C2/c
a [Å]	22.880(8)
b [Å]	11.076(3)
c [Å]	22.299(4)
a [°]	90
β [°]	102.263(12)
γ [°]	90
Volume [Å]	5522(3)
Z	8
ρ_{calc} [g/cm^3]	1.314
μ [mm^{-1}]	0.602
F(000)	2264.0
Crystal size [mm^3]	0.23 × 0.18 × 0.11
Radiation	MoKα (λ = 0.71073)
2Θ range for data collection [°]	4.346 to 59.986
Index ranges	-32 ≤ h ≤ 32, -15 ≤ k ≤ 15, -31 ≤ l ≤ 31
Reflections collected	182009
Independent reflections	8037 [R_{int} = 0.0955, R_{sigma} = 0.0370]
Data/restraints/parameters	8037/0/332
Goodness-of-fit on F^2	1.037
Final R indexes [I>=2σ (I)]	R_1 = 0.0292, wR_2 = 0.0589
Final R indexes [all data]	R_1 = 0.0375, wR_2 = 0.0628
Largest diff. peak/hole [e Å^{-3}]	0.52/-0.96

^{Me}Ar*$_2$SnCl$_2$ (**6**)

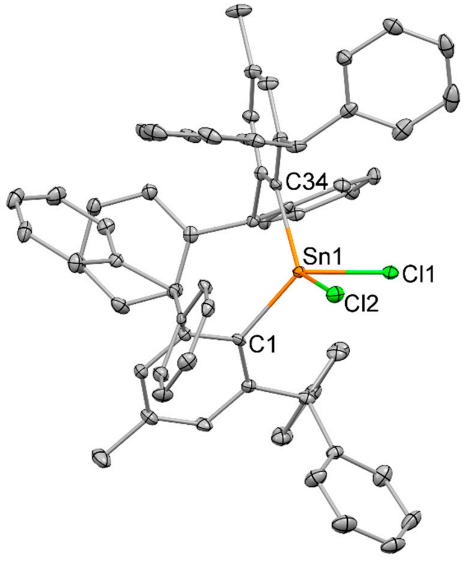

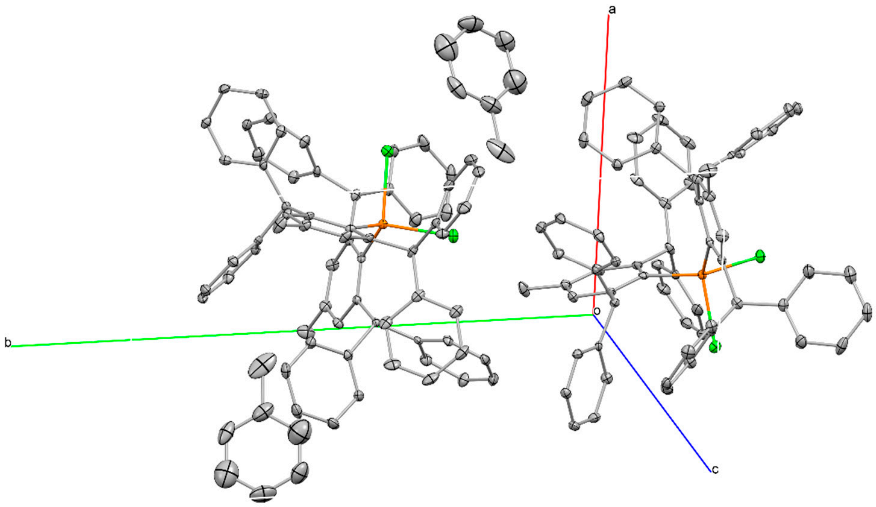

Figure S 59 Crystal structure and packing of ^{Me}Ar*$_2$SnCl$_2$ (**6**). All non-hydrogen atoms shown as 30% shaded ellipsoids. Hydrogen atoms are omitted for clarity. Selected bond lengths [Å] and angels [°]: Sn1-C1 2.155(5), Sn1-C34 2.159(6), Sn1-Cl1 2.402(2), Sn1-Cl2 2.344(2), C1-Sn1-C34 119.5(2), C1-Sn1-Cl2 103.0(2), C1-Sn1-Cl1 119.7(2), C34-Sn1-Cl1 97.6(2), C34-Sn1-Cl2 119.5(2); Cl1-Sn1-Cl2 95.82(5).

Table S 5 Crystal data and structure refinement $^{Me}Ar^{*}{}_2SnCl_2$ (**6**).

CCDC number	1983433
Empirical formula	$C_{69.5}H_{58}Cl_2Sn$
Formula weight	1082.75
Temperature/K	100.01
Crystal system	monoclinic
Space group	$P2_1$
a [Å]	10.5065(5)
b [Å]	20.2332(10)
c [Å]	13.5792(6)
a [°]	90
β [°]	93.415(2)
γ [°]	90
Volume [Å]	2881.5(2)
Z	2
ρ_{calc} [g/cm^3]	1.248
μ [mm^{-1}]	0.576
F(000)	1118.0
Crystal size [mm^3]	0.18 × 0.13 × 0.10
Radiation	MoKα (λ = 0.71073)
2Θ range for data collection [°]	3.616 to 51.998
Index ranges	$-12 \leq h \leq 12$, $-24 \leq k \leq 24$, $-16 \leq l \leq 16$
Reflections collected	44933
Independent reflections	11163 [R_{int} = 0.0690, R_{sigma} = 0.0681]
Data/restraints/parameters	11163/43/676
Goodness-of-fit on F^2	1.040
Final R indexes [I>=2σ (I)]	R_1 = 0.0431, wR_2 = 0.1115
Final R indexes [all data]	R_1 = 0.0484, wR_2 = 0.1151
Largest diff. peak/hole [e Å^{-3}]	1.31/-0.47

$^{i\text{Pr}}$Ar*$SnMe_3$ (**7**)

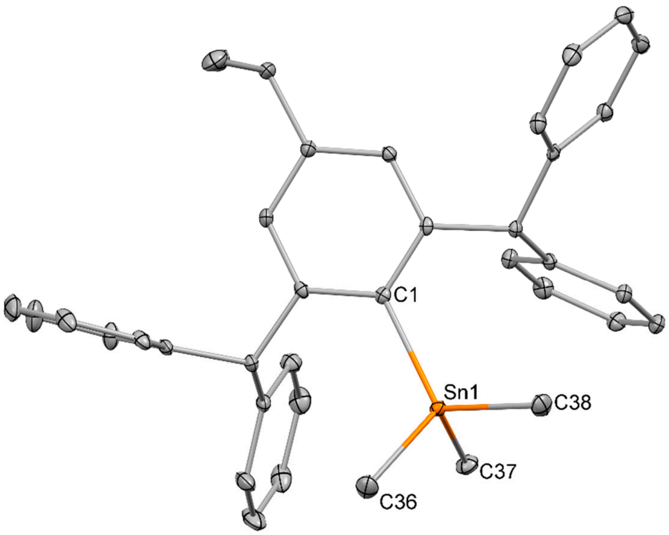

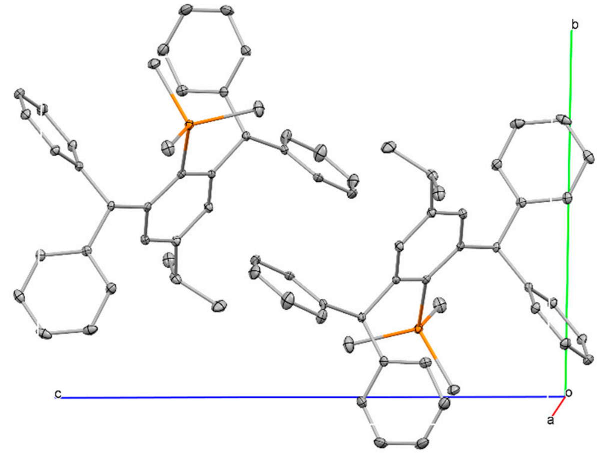

Figure S 60 Crystal structure and packing of $^{i\text{Pr}}$Ar*$SnMe_3$ (**7**). All non-hydrogen atoms shown as 30% shaded ellipsoids. Hydrogen atoms are omitted for clarity. Selected bond lengths [Å] and angels [°]: Sn1-C1 2.189(1), Sn1-C36 2.162(2), Sn1-C37 2.141(2), Sn1-C38 2.156(2), C1-Sn1-C36 113.04(6), C1-Sn1-C37 11.75(6), C1-Sn1-C38 114.8(6).

Table S 6 Crystal data and structure refinement ^{iPr}Ar*$SnMe_3$ (**7**).

CCDC number	1983432
Empirical formula	$C_{19}H_{20}Sn_{0.5}$
Formula weight	307.69
Temperature/K	100.0
Crystal system	triclinic
Space group	P-1
a [Å]	9.7217(4)
b [Å]	11.2216(5)
c [Å]	15.2377(8)
a [°]	90.092(2)
β [°]	90.965(2)
γ [°]	107.899(2)
Volume [Å]	1581.60(13)
Z	4
ρ_{calc} [g/cm^3]	1.292
μ [mm^{-1}]	0.831
F(000)	636.0
Crystal size [mm^3]	0.28 × 0.24 × 0.13
Radiation	MoKα (λ = 0.71073)
2Θ range for data collection [°]	3.814 to 58
Index ranges	$-13 \leq h \leq 13$, $-15 \leq k \leq 15$, $-20 \leq l \leq 20$
Reflections collected	63168
Independent reflections	8401 [R_{int} = 0.0415, R_{sigma} = 0.0276]
Data/restraints/parameters	8401/0/357
Goodness-of-fit on F^2	1.043
Final R indexes [I>=2σ (I)]	R_1 = 0.0234, wR_2 = 0.0529
Final R indexes [all data]	R_1 = 0.0268, wR_2 = 0.0543
Largest diff. peak/hole [e Å^{-3}]	0.84/-0.31

^{iPr}Ar*SnCl$_2$Me (**8**)

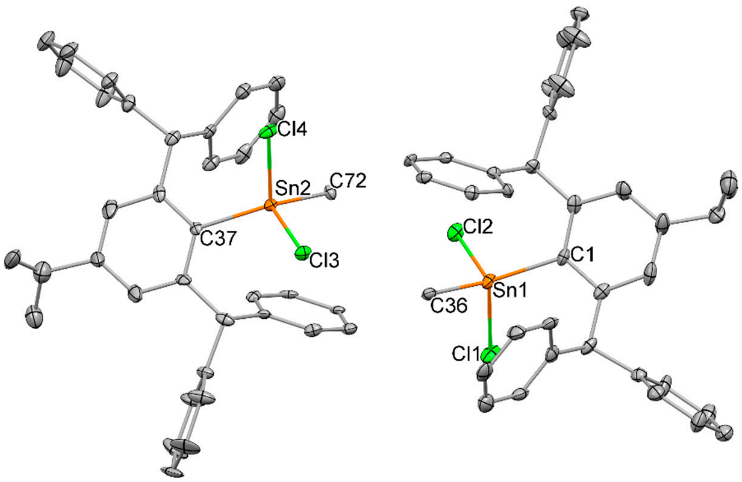

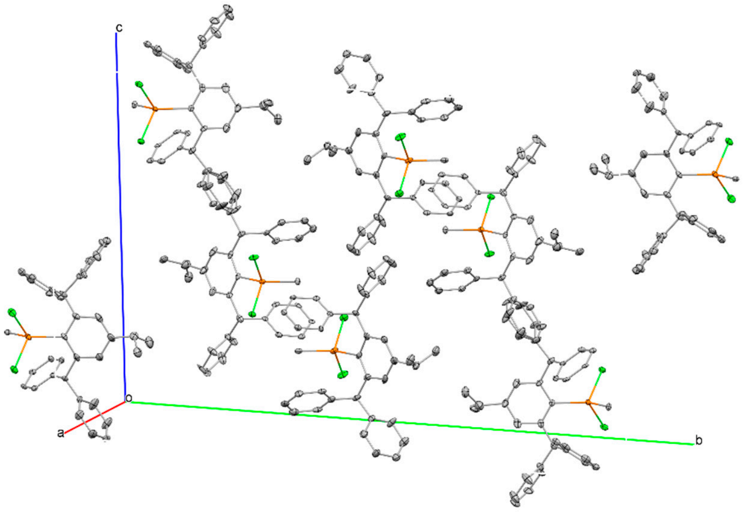

Figure S 61 Crystal structure and packing of ^{iPr}Ar*SnCl$_2$Me (**8**). All non-hydrogen atoms shown as 30% shaded ellipsoids. Hydrogen atoms are omitted for clarity. There are two independent molecules in the asymmetric unit. Selected bond lengths [Å] and angels [°]: Sn1-C1 2.141(7), Sn2-C37 2.140(6), Sn1-Cl1 2.373(2), Sn1-Cl2 2.375(2), Sn1-Cl3 2.369(2), Sn1-Cl4 2.381(2), C1-Sn1-C36 131.3(3), C1-Sn1-Cl1 109.3(2), C1-Sn1-Cl2 107.9(2), C37-Sn2-C72 130.0(3), C36-Sn1-Cl1 103.6(2), C36-Sn1-Cl2 105.6(2), C37-Sn1-Cl3 109.0(2), C37-Sn2-Cl4 109.2(2), C72-Sn2-Cl3 106.0(2), C72-Sn2-Cl4 103.6(2), Cl1-Sn1-Cl2 92.47(7), Cl3-Sn2-Cl4 92.47(7),

Table S 7 Crystal data and structure refinement ^{iPr}Ar*$SnCl_2Me$ (**8**).

CCDC number	1983435
Empirical formula	$C_{36}H_{33.5}Cl_2Sn$
Formula weight	655.72
Temperature/K	99.99
Crystal system	monoclinic
Space group	$P2_1/n$
a [Å]	9.1716(6)
b [Å]	37.619(3)
c [Å]	22.3889(17)
a [°]	90
β [°]	92.077(3)
γ [°]	90
Volume [Å]	7719.6(10)
Z	8
ρ_{calc} [g/cm^3]	1.128
μ [mm^{-1}]	0.819
F(000)	2668.0
Crystal size [mm^3]	0.23 × 0.17 × 0.12
Radiation	MoKα (λ = 0.71073)
2Θ range for data collection [°]	2.118 to 53.998
Index ranges	$-11 \leq h \leq 11, -48 \leq k \leq 47, -26 \leq l \leq 28$
Reflections collected	99078
Independent reflections	16647 [R_{int} = 0.1183, R_{sigma} = 0.0908]
Data/restraints/parameters	16647/152/788
Goodness-of-fit on F^2	1.092
Final R indexes [I>=2σ (I)]	R_1 = 0.0879, wR_2 = 0.1913
Final R indexes [all data]	R_1 = 0.1086, wR_2 = 0.2000
Largest diff. peak/hole [e Å^{-3}]	1.84/-1.77

$^{i\mathrm{Pr}}$Ar*SnI$_3$ (**9**)

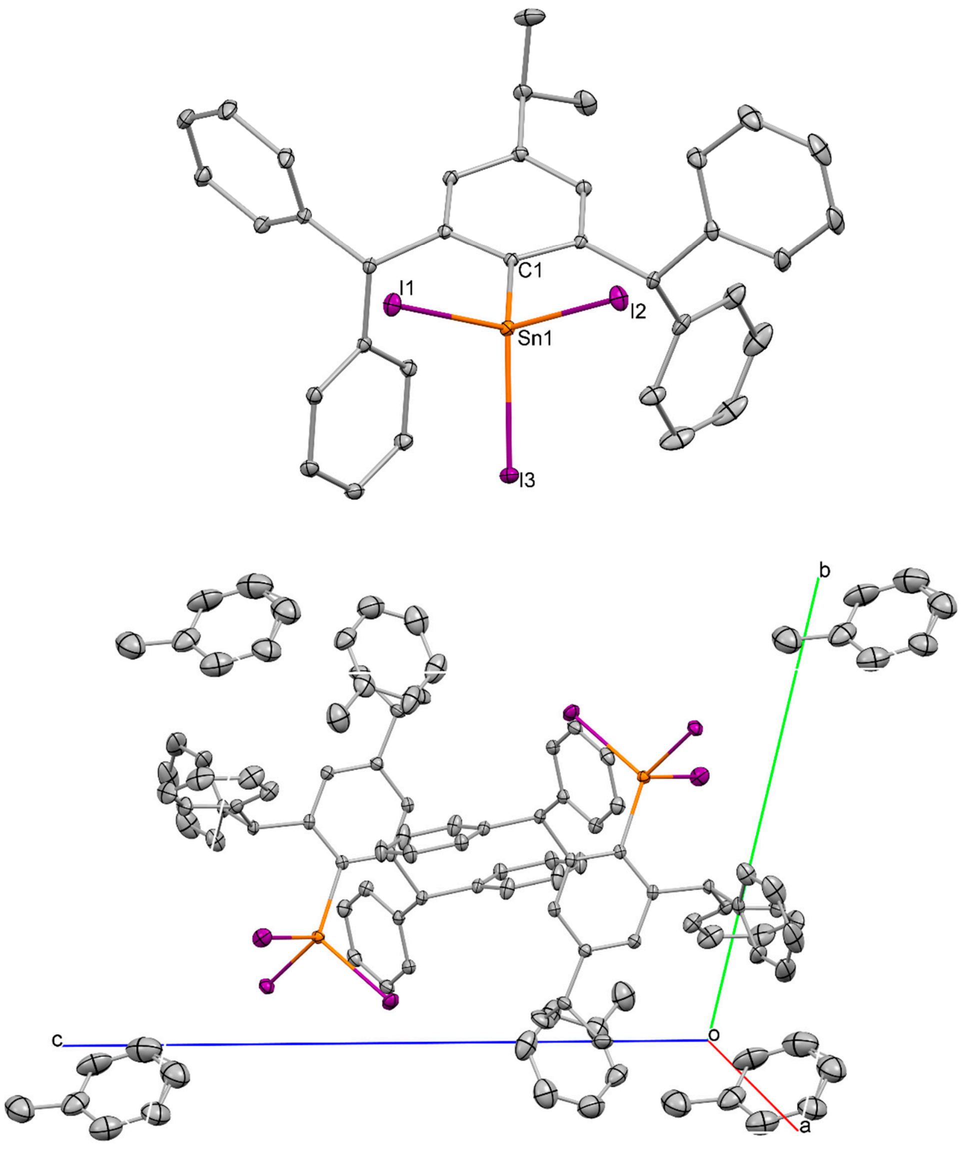

Figure S 62 Crystal structure and packing of $^{i\mathrm{Pr}}$Ar*SnI$_3$ (**9**). All non-hydrogen atoms shown as 30% shaded ellipsoids. Hydrogen atoms are omitted for clarity. Selected bond lengths [Å] and angels [°]: Sn1-C1 2.161(2), Sn1-I1 2.7130(4), Sn1-I2 2.6964(4), Sn2-I3 2.6721(4), C1-Sn-I1 116.94(6), C1-Sn1-I2 113.06(6), C1-Sn1-I3 114.20(6), I1-Sn1-I2 97.31(1), I1-Sn1-I3 106.04(1), I2-Sn1-I3 107.66(1).

Table S 8 Crystal data and structure refinement ^{iPr}Ar*SnI_3 (**9**).

CCDC number	1983441
Empirical formula	$C_{45.5}H_{43}I_3Sn$
Formula weight	1089.19
Temperature/K	99.99
Crystal system	triclinic
Space group	P-1
a [Å]	10.3638(4)
b [Å]	12.6960(5)
c [Å]	17.4605(7)
a [°]	103.977(2)
β [°]	102.034(2)
γ [°]	103.476(2)
Volume [Å]	2080.94(14)
Z	2
ρ_{calc} [g/cm^3]	1.738
μ [mm^{-1}]	2.869
F(000)	1050.0
Crystal size [mm^3]	0.51 × 0.27 × 0.26
Radiation	MoKα (λ = 0.71073)
2Θ range for data collection [°]	3.464 to 60
Index ranges	$-14 \leq h \leq 14$, $-17 \leq k \leq 17$, $-24 \leq l \leq 24$
Reflections collected	156214
Independent reflections	12132 [R_{int} = 0.0411, R_{sigma} = 0.0172]
Data/restraints/parameters	12132/42/470
Goodness-of-fit on F^2	1.112
Final R indexes [I>=2σ (I)]	R_1 = 0.0223, wR_2 = 0.0454
Final R indexes [all data]	R_1 = 0.0274, wR_2 = 0.0497
Largest diff. peak/hole [e Å^{-3}]	1.36/-0.81

^{Me}Ar*SnI$_3$ (**10**)

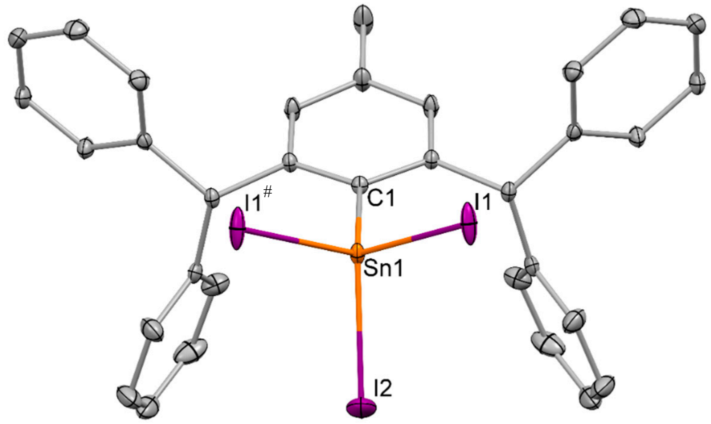

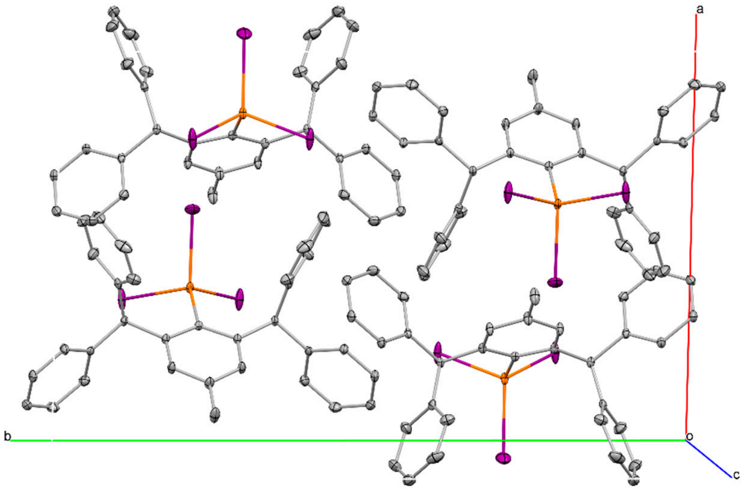

Figure S 63 Crystal structure and packing of ^{Me}Ar*SnI$_3$ (**10**). All non-hydrogen atoms shown as 30% shaded ellipsoids. Hydrogen atoms are omitted for clarity. Selected bond lengths [Å] and angels [°]: Sn1-C1 2.158(3), Sn1-I1 2.6994(3), Sn1-I2 2.6752(4), C1-Sn1-I1 116.16(6), C1-Sn1-I2 115.11(10), C1-Sn1-I1# 116.16(5), I1-Sn1-I2 105.743(11), I-Sn1-I1# 95.665(12).

Table S 9 Crystal data and structure refinement $^{Me}Ar^{*}SnI_3$ (**10**).

CCDC number	1983440
Empirical formula	$C_{33}H_{27}I_3Sn$
Formula weight	922.93
Temperature/K	99.99
Crystal system	orthorhombic
Space group	Pnma
a [Å]	14.4770(5)
b [Å]	23.2264(8)
c [Å]	9.1969(3)
a [°]	90
β [°]	90
γ [°]	90
Volume [Å]	3092.44(18)
Z	4
ρ_{calc} [g/cm^3]	1.982
μ [mm^{-1}]	3.841
F(000)	1736.0
Crystal size [mm^3]	0:33 × 0:26 × 0:19
Radiation	MoKα (λ = 0.71073)
2Θ range for data collection [°]	4.764 to 55.994
Index ranges	$-19 \leq h \leq 19$, $-30 \leq k \leq 30$, $-12 \leq l \leq 12$
Reflections collected	104660
Independent reflections	3823 [R_{int} = 0.0464, R_{sigma} = 0.0129]
Data/restraints/parameters	3823/0/175
Goodness-of-fit on F^2	1.061
Final R indexes [I>=2σ (I)]	R_1 = 0.0242, wR_2 = 0.0489
Final R indexes [all data]	R_1 = 0.0280, wR_2 = 0.0519
Largest diff. peak/hole [e Å^{-3}]	3.19/-3.12

^{iPr}Ar*$_2$SnH$_2$ (**11**)

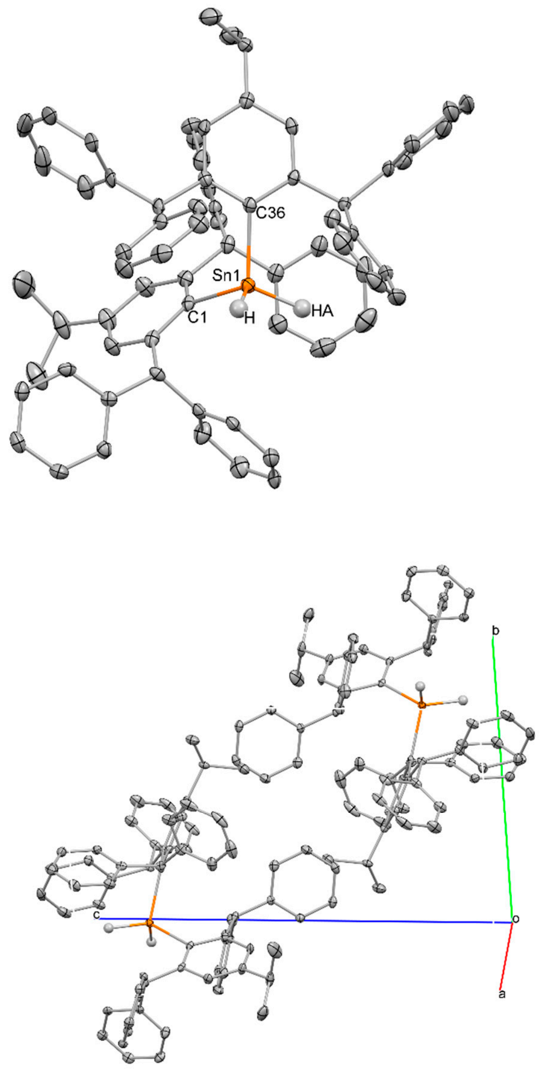

Figure S 64 Crystal structure and packing of ^{iPr}Ar*$_2$SnH$_2$ (**11**). All non-hydrogen atoms shown as 30% shaded ellipsoids. Hydrogen atoms except Sn-H atoms are omitted for clarity. Selected bond lengths [Å] and angels [°]: Sn1-C1 2.187(3), Sn1-C36 2.171(3), Sn1-H 1.71(3), Sn1-HA 1.70(4), C1-Sn1-C36 105.9(1), C1-Sn-H 114(1), C1-Sn1-HA 109(1), C36-Sn1-H 108(1), C36-Sn1-HA 111(1), H-Sn1-HA 109(2).

Table S 10 Crystal data and structure refinement $^{iPr}Ar^*{}_2SnH_2$ (**11**).

CCDC number	1983434
Empirical formula	$C_{70}H_{64}Sn$
Formula weight	1023.90
Temperature/K	100.03
Crystal system	triclinic
Space group	P-1
a [Å]	13.5263(12)
b [Å]	13.8425(11)
c [Å]	17.1579(14)
a [°]	75.399(3)
β [°]	69.368(3)
γ [°]	66.266(3)
Volume [Å]	2729.4(4)
Z	2
ρ_{calc} [g/cm^3]	1.246
μ [mm^{-1}]	0.510
F(000)	1068.0
Crystal size [mm^3]	0.14 × 0.11 × 0.06
Radiation	MoKα (λ = 0.71073)
2Θ range for data collection [°]	3.43 to 50.996
Index ranges	$-16 \leq h \leq 16$, $-16 \leq k \leq 16$, $-20 \leq l \leq 20$
Reflections collected	36230
Independent reflections	10101 [R_{int} = 0.1892, R_{sigma} = 0.2109]
Data/restraints/parameters	10101/1/650
Goodness-of-fit on F^2	0.830
Final R indexes [I>=2σ (I)]	R_1 = 0.0575, wR_2 = 0.0872
Final R indexes [all data]	R_1 = 0.1179, wR_2 = 0.1020
Largest diff. peak/hole [e Å^{-3}]	0.63/-0.80

$^{Me}Ar*_2SnH_2$ (**12**)

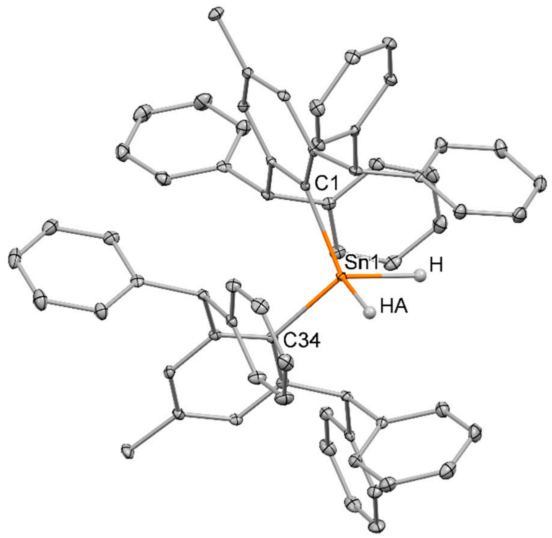

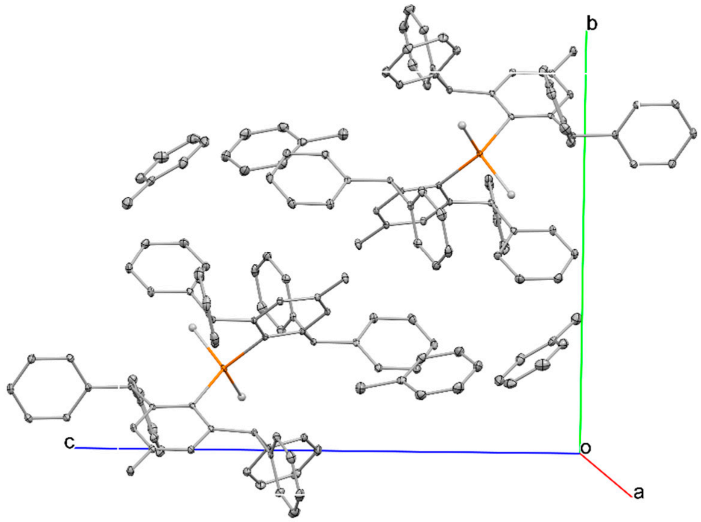

Figure S 65 Crystal structure and packing of $^{Me}Ar*_2SnH_2$ (**12**). All non-hydrogen atoms shown as 30% shaded ellipsoids. Hydrogen atoms except Sn-H atoms are omitted for clarity. Selected bond lengths [Å] and angels [°]: Sn1-C1 2.188(2), Sn1-C34 2.186(2), Sn1-H 1.79(2), Sn1-HA 1.80(2), C1-Sn1-C34 109.49(8), C1-Sn1-H 110.1(6), C1-Sn1-HA 114.1(7), C34-Sn1-H 117.3(6), C34-Sn1-HA 105.0(7), H-Sn1-HA 100.7(9).

Table S 11 Crystal data and structure refinement $^{Me}Ar^{*}_{2}SnH_{2}$ (**12**).

CCDC number	1983439
Empirical formula	$C_{80}H_{72}Sn$
Formula weight	1152.06
Temperature/K	99.96
Crystal system	triclinic
Space group	P-1
a [Å]	11.1312(6)
b [Å]	15.2126(7)
c [Å]	18.4189(10)
a [°]	90.282(3)
β [°]	99.601(3)
γ [°]	104.135(3)
Volume [Å]	2978.8(3)
Z	2
ρ_{calc} [g/cm^3]	1.284
μ [mm^{-1}]	0.475
F(000)	1204.0
Crystal size [mm^3]	0.23 × 0.16 × 0.1
Radiation	MoKα (λ = 0.71073)
2Θ range for data collection [°]	2.244 to 56
Index ranges	$-14 \leq h \leq 14$, $-20 \leq k \leq 20$, $-24 \leq l \leq 24$
Reflections collected	114184
Independent reflections	14391 [R_{int} = 0.0918, R_{sigma} = 0.0729]
Data/restraints/parameters	14391/3/740
Goodness-of-fit on F^2	1.068
Final R indexes [I>=2σ (I)]	R_1 = 0.0423, wR_2 = 0.0873
Final R indexes [all data]	R_1 = 0.0570, wR_2 = 0.0935
Largest diff. peak/hole [e Å^{-3}]	2.43/-1.31

^{iPr}Ar*SnH$_3$ (**13**)

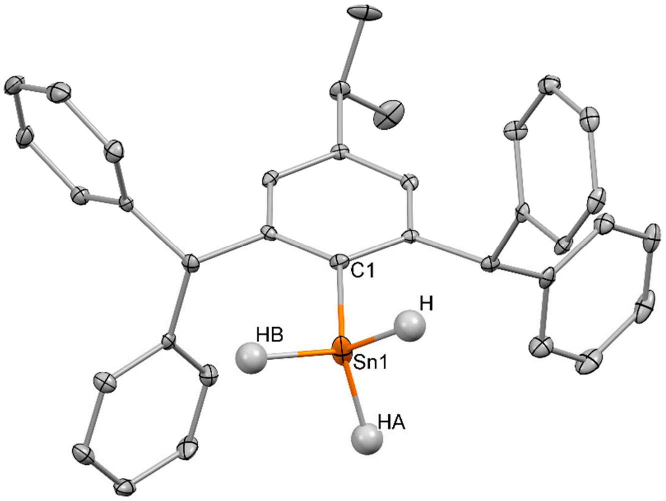

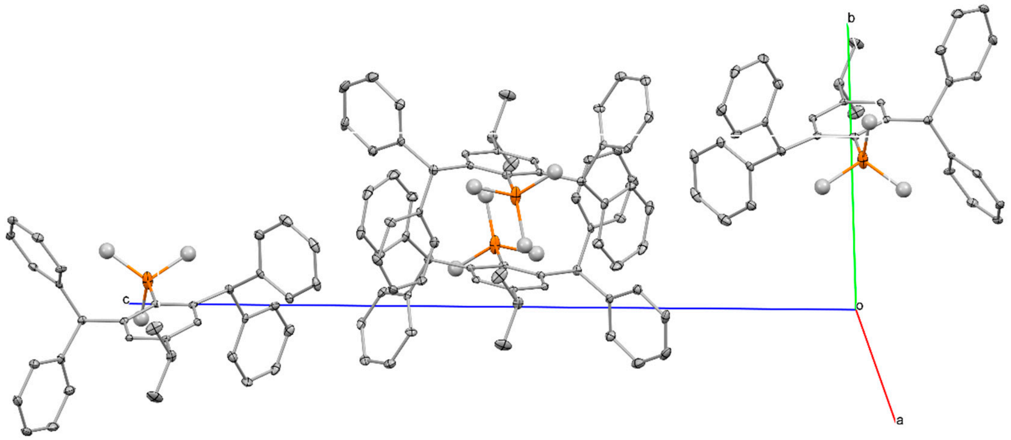

Figure S 66 Crystal structure and packing of ^{iPr}Ar*SnH$_3$ (**13**). All non-hydrogen atoms shown as 30% shaded ellipsoids. Hydrogen atoms except Sn-H atoms are omitted for clarity. Selected bond lengths [Å] and angels [°]: Sn1-C1 2.153(2).

Table S 12 Crystal data and structure refinement ^{iPr}Ar*SnH_3 (**13**).

CCDC number	1983438
Empirical formula	$C_{35}H_{34}Sn$
Formula weight	573.31
Temperature/K	99.99
Crystal system	monoclinic
Space group	$P2_1/n$
a [Å]	11.0869(5)
b [Å]	10.5075(5)
c [Å]	25.1315(11)
a [°]	90
β [°]	102.437(2)
γ [°]	90
Volume [Å]	2859.0(2)
Z	4
ρ_{calc} [g/cm^3]	1.332
μ [mm^{-1}]	0.914
F(000)	1176.0
Crystal size [mm^3]	0.28 × 0.14 × 0.09
Radiation	MoKα (λ = 0.71073)
2Θ range for data collection [°]	4.216 to 56
Index ranges	-14 ≤ h ≤ 14, -13 ≤ k ≤ 13, -33 ≤ l ≤ 32
Reflections collected	57736
Independent reflections	6898 [R_{int} = 0.0621, R_{sigma} = 0.0376]
Data/restraints/parameters	6898/0/333
Goodness-of-fit on F^2	1.103
Final R indexes [I>=2σ (I)]	R_1 = 0.0446, wR_2 = 0.0957
Final R indexes [all data]	R_1 = 0.0640, wR_2 = 0.1038
Largest diff. peak/hole [e Å^{-3}]	0.95/-0.58

^{Me}Ar*SnH$_3$ (**14**)

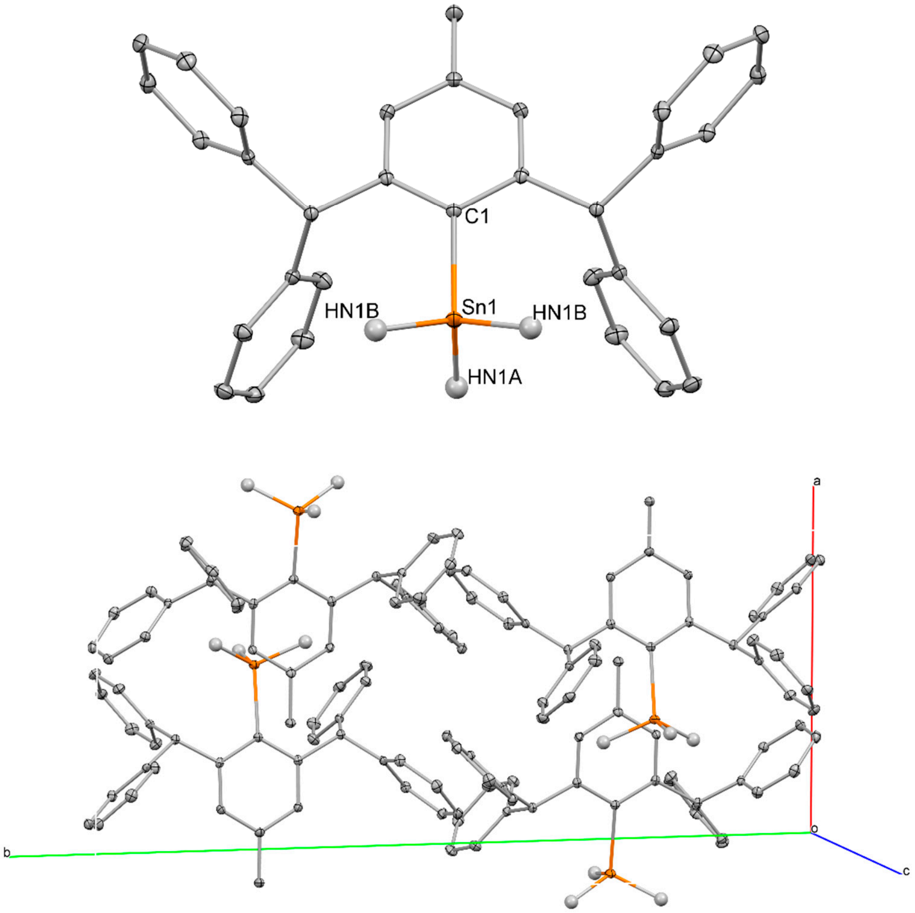

Figure S 67 Crystal structure and packing of ^{Me}Ar*SnH$_3$ (**14**). All non-hydrogen atoms shown as 30% shaded ellipsoids. Hydrogen atoms except Sn-H atoms are omitted for clarity. Selected bond lengths [Å] and angels [°]: Sn1-C1 2.167(3).

Table S 13 Crystal data and structure refinement $^{Me}Ar^{*}SnH_3$ (**14**).

CCDC number	1983437
Empirical formula	$C_{33}H_{30}Sn$
Formula weight	545.26
Temperature/K	99.98
Crystal system	orthorhombic
Space group	Pnma
a [Å]	10.4192(4)
b [Å]	24.9793(8)
c [Å]	9.9915(4)
a [°]	90
β [°]	90
γ [°]	90
Volume [Å]	2600.43(17)
Z	4
ρ_{calc} [g/cm^3]	1.393
μ [mm^{-1}]	1.001
F(000)	1112.0
Crystal size [mm^3]	0.26 × 0.17 × 0.09
Radiation	MoKα (λ = 0.71073)
2Θ range for data collection [°]	3.26 to 56
Index ranges	$-13 \leq h \leq 13$, $-33 \leq k \leq 33$, $-13 \leq l \leq 13$
Reflections collected	93692
Independent reflections	3201 [R_{int} = 0.0604, R_{sigma} = 0.0178]
Data/restraints/parameters	3201/2/162
Goodness-of-fit on F^2	1.006
Final R indexes [I>=2σ (I)]	R_1 = 0.0306, wR_2 = 0.0779
Final R indexes [all data]	R_1 = 0.0331, wR_2 = 0.0801
Largest diff. peak/hole [e Å^{-3}]	2.39/-1.39
